# A comparison of the associations between bone health and three different intensities of accelerometer-derived habitual physical activity in children and adolescents: a systematic review

**DOI:** 10.1007/s00198-021-06218-5

**Published:** 2022-01-28

**Authors:** Gemma Brailey, Brad Metcalf, Rebecca Lear, Lisa Price, Sean Cumming, Victoria Stiles

**Affiliations:** 1 Sport and Health Sciences, College of Life and Environmental Sciences, University of Exeter, Exeter, UK; 2 Department for Health, University of Bath, Bath, UK

**Keywords:** Accelerometry, Bone, Children, Physical activity

## Abstract

Positive associations have been identified between bone outcomes and accelerometer-derived moderate (MPA) and vigorous (VPA) physical activity (PA) in youth; however, it remains unclear which intensity is most beneficial. This systematic review aimed to summarise accelerometer-derived methods used to estimate habitual PA in children and adolescents and determine whether the magnitude of association was consistently stronger for a particular intensity (MPA/MVPA/VPA). Observational studies assessing associations between accelerometer-derived MPA and/or MVPA *and* VPA with bone outcomes in children and adolescents (≤ 18 years) were identified in MEDLINE, EMBASE, Web of Science, SPORTDiscus and the Cochrane Central Register of Controlled Trials. Thirty articles were included (total *n* = 20,613 (10,077 males), 4–18 years). Chi-square tests determined whether the proportion of significant associations and strongest within-study associations differed significantly between intensities. Results demonstrated that accelerometer methods were highly variable between studies. Of the 570 associations analysed, 186 were significant (*p* < 0.05). The proportion of within-study strongest associations differed by PA intensity (3 × 2 *χ*^2^ = 86.6, *p* < 0.001) and was significantly higher for VPA (39%) compared to MVPA (5%; 2 × 2 *χ*^2^ = 55.3, *p* < 0.001) and MPA (9%, 2 × 2 *χ*^2^ = 49.1, *p* < 0.001). Results indicated a greater benefit of VPA over MPA/MVPA; however, variability in accelerometer-derived methods used prevents the precise bone-benefitting amount of VPA from being identified. Long epochs and numerous intensity cut-point definitions mean that bone-relevant PA has likely been missed or misclassified in this population. Future research should explore the use of shorter epochs (1 s) and identify bone-specific activity intensities, rather than using pre-defined activity classifications more relevant to cardiovascular health.

**Supplementary Information:**

The online version contains supplementary material available at 10.1007/s00198-021-06218-5.

## Introduction

Osteoporosis, a disease characterised by low bone mass and increased fracture risk [1], is associated with greater disability than that caused by many cancers and chronic non-communicable diseases [2]. The condition affects 3.2 million people aged over 50 in the UK alone and conveys a substantial economic burden, estimated to cost the UK around £5.5 billion by 2025 [3]. Despite being considered primarily a condition of old age [4], around 60% of osteoporosis risk can be attributed to the amount of bone that is acquired by attainment of the peak bone mass (PBM) in early adulthood [5]. Whilst genetics is suggested to account for 60–80% of the variability in PBM, physical activity (PA) during growth is one of the most important factors influencing the remaining modifiable component [6]. However, despite many observational [7–9] and intervention studies [10–12] demonstrating an osteogenic effect from PA, the recommended dose of PA (frequency, intensity, duration and type) that benefits bone health in children and adolescents remains unclear [6].

Summarising the precise dose–response effect that PA has on bone health may be hampered by the study designs reviewed and the variety of methods used to estimate PA. Many previous reviews summarising the relationship between PA and bone health have focused on PA or exercise interventions [13–17], which only epitomise a small subset of activity behaviour (acute changes over a short duration) and do not represent everyday habitual activity in the general population. When summarising the effect of habitual PA on bone health, the majority of observational studies included in previous reviews [18, 19] have mostly used self-reported methods to obtain information about activity behaviour (8/9 studies in [19] and 7/10 in [18]). A problem with self-reported methods of estimating PA is that they provide imprecise information regarding the intensity, duration, frequency and pattern of accumulating activity, especially in children, due to their lower cognitive function and inability to accurately recall information and estimate time [20]. In recent years, the use of accelerometers to monitor habitual PA in relation to health outcomes in children has become commonplace. Accelerometers are small, lightweight and unobtrusive and allow several days or weeks of PA to be assessed over short sampling intervals of minutes or seconds [21]. Whilst accelerometers provide an objective measure of PA free from the random and systematic errors associated with self-report [22], there still remain several methodological challenges related to the collection, processing and interpretation of the acceleration data [23].

As minimal attention has been directed towards standardising methodological approaches [21], researchers are often required to make decisions regarding the accelerometer model (which may output raw and/or proprietary count-based data), wear criteria (definition of a valid day, non-wear time within a day, number of valid days required for inclusion) and whether to analyse a raw acceleration output directly, average outputs (raw/counts) over a certain length of epoch, classify the magnitude of the output into categories in an attempt to reflect different physiological intensities and if so, what cut-points to apply to facilitate this [23]. All of these may have a bearing on the quantity and quality of accelerometer data obtained [23]. Many studies evaluating relationships between accelerometer-derived estimates of habitual PA and health outcomes also only report activity as moderate-to-vigorous PA (MVPA)—a metric that is included in PA guidelines and is proposed to reflect an intensity of activity that places a moderate-vigorous cardiovascular (aerobic) demand on the body. Activities upwards from and including brisk walking are suggested to illicit this cardiovascular demand. Whilst significant, positive associations between MVPA and bone health outcomes have been observed [8, 9], it is likely that these associations are driven by activities of a more vigorous intensity, rather than those at the lower end of moderate intensity, such as walking, as walking has been shown to be of little or no benefit to bone health [24]. A broad MVPA classification may therefore make it difficult to discern the precise threshold of intensity driving an association between PA and bone and could also risk a non-osteogenic type of activity (e.g. walking) being recommended to promote bone health.

A recently published systematic review [25] examining the associations between bone health outcomes and objectively measured PA intensities (sedentary, light (LPA), moderate (MPA), MVPA, vigorous (VPA) and total PA) in children and adolescents demonstrated that both MPA and VPA positively predicted bone development in this population. However, the magnitude of associations between these intensities and bone outcomes within studies was not compared. It is therefore not clear whether there is a consistently greater benefit of VPA over and above MPA in relation to bone health outcomes. The independent associations and greater benefits of objectively measured VPA over other PA intensities such as MPA or MVPA have been recently recognised for several other health outcomes in youth [26]. When looking at the magnitude of the relationships between MPA and/or MVPA and VPA, Gralla et al. [26] found that VPA was consistently a stronger predictor of improved body composition and fitness in comparison to MPA and/or MVPA. These findings emphasise the importance of stratifying for higher intensity activity and assessing the strength of associations between outcomes and independent activity intensities when trying to identify more precise dose–response relationships. With particular reference to bone health, adaptations in bone are threshold driven and bought about by activities that create dynamic, rapidly applied loads with a high magnitude of impact. Activities that elicit higher impacts provide a larger osteogenic effect [27]. Therefore, when bone outcomes are of interest, it would be particularly important to summarise findings from studies that have objectively and independently assessed the association of higher intensity activity over and above other intensities of habitual activity.

An assessment of the independent contributions that both moderate- (commonly referred to as MPA/MVPA) and high-intensity (commonly referred to as VPA) activity have on bone health will likely be influenced by the different accelerometer methods used to obtain PA data between studies. A summary of the range of methods employed will provide essential information to help identify potential methodological issues in the objective measurement of PA in relation to bone health. It remains important, however, to establish whether a particular intensity appears to be consistently more beneficial to the bone. This combined level of information will help to inform the direction that future research in the objective assessment of habitual PA in relation to bone health must take to improve the precision of measuring bone-specific PA and will also facilitate the identification of more specific dose–response relationships between PA and bone health in children and adolescents.

The review will therefore (1) summarise the accelerometry data collection and processing methods used in studies to estimate habitual PA when relating it to bone outcomes in children and adolescents; (2) determine whether habitual PA of at least moderate intensity (MPA/MVPA and VPA) is related to bone health in children and adolescents (independently of activities at a lower intensity); and (3) despite variations in accelerometer methods used to capture the data, determine whether the magnitude of association between PA and bone outcome measures is consistently stronger for a particular intensity of habitual PA (MPA/MVPA or VPA).

## Methods

This review was guided by the Centre for Reviews and Dissemination’s guidance for undertaking reviews [28] and the COSMOS-E guidelines on conducting systematic reviews and meta-analyses of observational studies of aetiology [29] and is reported in accordance with the Preferred Reporting Items for Systematic reviews and Meta-Analyses (PRISMA) guidelines [30]. The review protocol is registered on the PROSPERO International Prospective Register of Systematic Reviews (https://www.crd.york.ac.uk/prospero/) under the registration number CRD42018106493.

### Search strategy

A detailed systematic electronic search combining free text and Medical Subject Headings (MesH) was conducted in several electronic databases (MEDLINE (Ovid) 1946–present with MEDLINE (OVID) in process and other non-indexed citations, EMBASE, Web of Science, SPORTDiscus and Cochrane Central Register of Controlled Trials) from their commencement up until May 4, 2020. Search terms relevant to physical activity (e.g. physical activity, habitual activity, MVPA, accelerometer, activity monitor, motion sensor) AND bone health (e.g. bone health, bone density, bone strength, bone structure) OR bone imaging methods (e.g. DXA or DEXA, quantitative ultrasound, quantitative tomography) AND children/adolescents (e.g. child, adolescent, paediatric or pediatric, youth) were used. An example of a full search conducted in the MEDLINE database is given in the Online Resource 1. The Yale MeSH analyser [31] was used on a selection of potentially relevant studies to identify important MeSH terms to include in the search and ensure vital terms had not been missed. There were no limits placed upon the search; however, only articles published in the English language were considered for inclusion. Review articles, editorials, conference abstracts or proceedings, unpublished articles or dissertations were not considered for inclusion. Manual searches of the reference lists of included papers and relevant review articles were conducted to identify any additional articles.

### Study selection and inclusion criteria

The review inclusion criteria were guided by the Population, Exposure, Control and Outcome(s) format outlined in the COSMOS-E guidelines for systematic reviews of epidemiological studies, which are in line with the Population, Exposure, Comparator, Outcome(s) and Study characteristics (PECOS) framework in the PRISMA guidelines. Two reviewers independently screened the titles and abstracts of all results from the electronic database search according to the pre-defined inclusion criteria. Any discrepancies were resolved through discussion and abstracts that were not eligible were discarded. Full-text articles for potentially relevant studies were obtained and screened by the same two reviewers. Studies that met the inclusion criteria were selected and included in the review. A third investigator was consulted if the reviewers were unable to reach a consensus on discrepancies.

For inclusion in the review, studies were required to have included generally healthy children and adolescents aged ≤ 18 years (including those who were overweight/obese), objectively measured habitual PA using an accelerometer, and to have reported VPA (or high-intensity activity) and MPA and/or MVPA (or moderate-intensity activity) on a continuous scale (e.g. minutes per day, proportion of total time, number of peaks per day) and measured their respective associations with at least one measure of bone health (e.g. strength, mass, structure). Studies reporting associations between bone outcomes and activity that were of a moderate (jogging/slow running) and high-intensity (e.g. faster running/jumping) but were not defined in terms of VPA and MPA and/or MVPA were included as a comparison of activity intensity could still be made and descriptions of activities within bands of intensity allowed respective bands to be included in MPA and VPA categories for the purposes of this review. Since habitual PA includes all types of bodily movement that result in energy expenditure [32], studies focusing solely on a particular subset of PA (e.g. exercise, sport, leisure time PA, school-time PA) were excluded as this does not portray habitual PA in its entirety and therefore does not concur with the aims of the review. Studies were required to be observational in design (cross-sectional and prospective), but intervention studies were considered for inclusion if associations between VPA and MPA and/or MVPA (or other intensity definitions) and bone outcomes had been conducted at baseline (cross-sectional analyses) or if there was a separate control group that could be considered as a cross-sectional or prospective analysis. If a number of studies drawing from the same cohort were identified, all were considered for inclusion in the review. Those measuring participants at different time points or that reported on different outcomes obtained through a separate imaging method or at additional anatomical sites were included. When multiple studies from the same cohort had reported on the same or similar outcomes with comparable analyses, the study that had the most complete descriptive information on the sample, activity intensities, bone outcomes and their respective associations that most closely coincided with the aims of the review was kept for inclusion. Studies were not excluded based on the imaging tool used to assess bone outcomes.

### Quality assessment

Following exclusion of studies that did not meet the inclusion criteria, the quality and risk of bias of included studies were assessed by two independent investigators using the National Institute of Health ‘Quality Assessment Tool for Observational Cohort and Cross-Sectional Studies’ [33]. This consists of 14 components that relate to the design of the study, selection bias, bias in both the exposure and outcome (habitual PA and bone health outcomes), follow-up and whether statistical analyses adjusted for key confounders. Characteristics including age, sex, ethnicity, maturational stage and skeletal or body size should all be considered for statistical adjustment to reduce residual variability in regression models and improve statistical power, since they are associated with bone measures during growth [6]. Studies were given an overall rating of ‘poor’, ‘fair’ or ‘good’ based on the responses to the 14 items (response can be yes, no, cannot determine, not reported or not applicable for each of the 14 items). Item 9, which assesses whether the exposure measure was clearly defined, valid and reliable, was modified to account for the accelerometer data inclusion criteria. Amongst children and adolescents, the minimum number of days needed to achieve a reliable depiction of habitual PA ranges from 4 to 9 days [34]. It is also accepted that 10 h of wear time is sufficient to qualify as a valid day [22], so studies including participants that had PA data for ≥ 4 days with ≥ 10 h of wear were given a ‘yes’ response to item 9, and those with fewer than this received a ‘no’ response to this item. Studies were not excluded based on the results of the quality assessment; however, study quality was taken into account when interpreting findings.

### Data extraction

A structured form was developed to extract the following data: authors, title, study design, participants (sample size, age, sex, maturity status), accelerometer measurement procedures (make and model, epoch length, wear location, number of days wear, valid days for inclusion, definition of non-wear time, MPA, MVPA and VPA cut-points used (or other intensity categories presented and how they were defined)), amount of activity for each intensity (e.g. minutes of MPA, MVPA and VPA), bone imaging tools, site(s) assessed and outcomes reported, statistical analyses and covariates, and observed associations (*R*^2^, *R*^2^ change, *r*, *β*, Std. *β*) between MPA, MVPA and VPA (or other moderate-/high-intensity PA classifications) and bone outcomes and their level of significance (*p*-value). When more than one regression model was presented, data were extracted for the most adjusted model. If the required information was not presented in the article, an email was sent to the corresponding author requesting it. If there was no response, a reminder email was sent, and if no reply was received, only the information provided in the paper was presented. Data extraction was cross-checked by reviewer 2.

### Data synthesis and analysis

Due to the large variability in the methods used to assess bone outcomes (e.g. DXA, pQCT, QUS), the anatomical sites assessed (e.g. total body, femoral neck, tibia, radius, calcaneus) and numerous outcomes reported (e.g. bone mineral content, bone mineral density, bone stiffness, cortical density, polar strength-strain index), the heterogeneity between studies meant that the results of many of the studies were not directly comparable and therefore it was considered inappropriate to conduct a meta-analysis. In the absence of a fully quantitative meta-analysis, a semi-quantitative approach was employed, using chi-square tests to determine which PA intensity (MPA, MVPA or VPA) had the greatest proportion of ‘statistically significant associations’ with a bone outcome and which intensity had the greatest proportion of ‘strongest within-study associations’. These two ‘proportions’ were derived from a two-stage ‘vote’ counting procedure: Stage 1 involved awarding a ‘vote’ to any ‘PA intensity vs bone outcome’ association that was statistically significant (*p* < 0.05). All activity intensities within a study could potentially receive a vote at this stage. Stage 2 compared the magnitude of the statistically significant ‘PA intensity vs bone outcome’ associations within a study and only the intensity—MPA and/or MVPA and VPA—with the strongest association with the bone outcome received a vote (total count per analysis at this stage could only be 0 or 1). Only positive associations could be deemed as the ‘strongest association’ as they are consistent with a greater benefit to bone outcomes. When the association was statistically significant and of the same magnitude for two PA intensities, each intensity was counted as a vote in stage 1, but no vote was cast at stage 2. When negative associations were observed, a vote was counted in stage 1, but no vote was cast at stage 2. To present the results for vote-counting, studies were grouped based on the method used to assess bone outcomes and were further organised by anatomical site. The results for the significant counts or most strongly associated counts are expressed as a percentage of the total number of counts available (%(*n*/*N*); total counts are the number of counts regardless of statistical significance) for each intensity as studies differed in the combination of intensities reported and therefore the total number of counts available was different for MPA, MVPA and VPA, respectively. A 3 × 2 chi-square (*χ*^2^) test was used to determine whether the proportions of ‘statistically significant associations’ and ‘strongest within-study associations’ vote counts differed between the three PA intensities. When this omnibus test determined that there were statistically significant differences (*p* < 0.05) between at least two of the PA intensities, a priori follow-up analyses were carried out in the form of two 2 × 2 chi-square tests—‘MPA vs VPA’ and ‘MVPA vs VPA’. The observed *p*-values of these 2 × 2 tests were multiplied by ‘2’ in order to adjust for multiple testing, creating a new Bonferroni-adjusted *p*-value. Fisher’s exact tests were used where the data violated the assumptions of a chi-square test. Table 1 includes all reported associations between PA and bone outcomes, regardless of statistical significance.

**Table 1:** Study characteristics, accelerometry methods used and the relationships reported between MPA and/or MVPA and VPA with measures of bone health in children and adolescents for the 30 reviewed studies. Studies are grouped and presented by imaging method and study type (cross-sectional or longitudinal/prospective) then by epoch length (≥ 60 s, ≤ 15 s, raw data) and alphabetically within each epoch group

Reference Study design Quality	Sample size (m/f) Age (mean ± SD) Maturity	PA measurement methods	Intensity thresholds used PA outcomes reported	Bone imaging method Anatomical site Outcomes	Statistical analyses Main results
Cardadeiro et al. 2010 [43] Cross-sectional ◆	*n* = 88 (48/40) m, 8.6 ± 0.4 years f, 8.5 ± 0.4 years Bone age: m, 9.0 ± 1.1 years; f, 8.6 ± 1.2 years	Actigraph GT1M **60-s epoch** Location: right hip Worn for: 7 days (except WB activities) For inclusion: ≥ 3 days with ≥ 600 min/day Non-wear: NR	MPA, 1952–5724 cpm MVPA, ≥ 1952 cpm VPA, ≥ 5724 cpm (min/day) **MPA**: m, 78 ± 3; f, 55 ± 3* **MVPA**: m, 88 ± 26; f, 62 ± 24* **VPA**: m, 10 ± 1; f, 7 ± 1*	DXA (QDR 1500, Hologic Inc.) Ward’s area BMD (g/cm^2^)	Linear regression analysis was used to assess the relationships between the quantified deviation from consensus shape image of the proximal femur and PA variables Regression was performed separately for boys and girls and for each independent variable Boys: **MPA**: sum *d*^2^ of predicted = 0.0382, *R*^2^ (%) = 7.1, *F* = 3.542, *df* = 32.14, *p* < 0.001; **MVPA**: sum *d*^2^ of predicted = 0.0342, *R*^2^ (%) = 6.3, *F* = 3.144, *df* = 32.15, *p* < 0.001; **VPA**: sum *d*^2^ of predicted = 0.0049, *R*^2^ (%) = 0.9, *F* = 0.422, *df* = 32.14, *p* = 0.998 Girls: **MPA**: sum *d*^2^ of predicted = 0.0066, *R*^2^ (%) = 1.2, *F* = 0.461, *df* = 32.12, *p* = 0.995; **MVPA**: sum *d*^2^ of predicted = 0.0079, *R*^2^ (%) = 1.4, *F* = 0.549, *df* = 32.12, *p* = 0.981 **VPA**: sum *d*^2^ of predicted = 0.0149, *R*^2^ (%) = 2.7, *F* = 1.055, *df* = 32.12, *p* = 0.384
Cardadeiro et al. 2012 [42] Cross-sectional ◆◆	*n* = 325 (164/161) 9.7 ± 0.3 years **European Youth Heart Study** Pre- and early pubertal (Tanner stages 1 and 2)	WAM 6471 **60-s epoch** (from methods ref) Location: right hip Worn for: 2 WK and 2 WE days (except WB activities) For inclusion: ≥ 3 days with ≥ 600 min/day Non-wear: ≥ 10 consecutive 0 counts	MPA, 2000–2999 cpm MVPA, ≥ 2000 cpm VPA, ≥ 2999 cpm (min/day) **MPA**: m, 169 ± 55; f, 142 ± 47* **MVPA**: m, 198 ± 70; f, 159 ± 56* **VPA**: m, 30 ± 21; f, 18 ± 14.3*	DXA (QDR 1500, software 4.76) PF subregions: FN, TR, IT BMD (g/cm^2^)	Stepwise regression was used to analyse associations between PA and BMD of PF subregions. Analysed with boys and girls pooled together, then separately Adjusted for sex (pooled analyses only), Tanner stage, body height and TB lean mass Femoral neck BMD: Boys: **MPA**: Std *β* = − 0.073, *p* = 0.440; **MVPA**: Std *β* = − 0.092, *p* = 0.440; **VPA**: Std *β* = 0.225, *R*^2^ = 0.051, *p* = 0.003 Girls: **MPA**: Std *β* = 0.109, *p* = 0.116; **MVPA**: Std *β* = 0.125, *p* = 0.072; **VPA**: Std *β* = 0.135, *p* = 0.06 All: **MPA**: Std *β* = − 0.003, *p* = 0.955; **MVPA**: Std *β* = − 0.004, *p* = 0.955; **VPA**: Std *β* = 0.191, *R*^2^ = 0.033, *p* < 0.001 Trochanter BMD: Boys: **MPA**: Std *β* = − 0.013, *p* = 0.893; **MVPA**: Std *β* = − 0.016, *p* = 0.893; **VPA**: Std *β* = 0.214, *R*^2^ = 0.046, *p* = 0.005 Girls: **MPA**: Std *β* = 0.064, *p* = 0.400; **MVPA**: Std *β* = 0.076, *p* = 0.400; **VPA**: Std *β* = 0.241, *R*^2^ = 0.056, *p* < 0.001 All: **MPA**: Std *β* = 0.031, *p* = 0.604; **MVPA**: Std *β* = 0.039, *p* = 0.604; **VPA**: Std *β* = 0.227, *R*^2^ = 0.073, *p* = 0.001 Intertrochanter BMD: Boys: **MPA**: Std *β* = − 0.081, *p* = 0.499; **MVPA**: Std *β* = − 0.063, *p* = 0.499; **VPA**: Std *β* = 0.159, *R*^2^ = 0.025, *p* = 0.033 Girls: **MPA**: Std *β* = 0.086, *p* = 0.263; **MVPA**: Std *β* = 0.102, *p* = 0.263; **VPA**: Std *β* = 0.213, *R*^2^ = 0.044, *p* = 0.001 All: **MPA**: Std *β* = 0.015, *p* = 0.798; **MVPA**: Std *β* = 0.019, *p* = 0.798; **VPA**: Std *β* = 0.182, *R*^2^ = 0.046, *p* < 0.001
Janz et al. 2004 [50] Cross-sectional ◆◆	*n* = 436 (204/232) m: 5.2 ± 0.4 years f: 5.3 ± 0.4 years NR	MTI 7164 **60-s epoch** Location: waist midaxillary line Worn for: 4 days (including 1 WE day) For inclusion: ≥ 8 h per day for 3 days Non-wear: NR	MPA, 527–2818 counts VPA, ≥ 2818 counts (min/day): **MPA**: m, 267 ± 44; f, 262 ± 44 **VPA**: m, 38 ± 19; f, 28 ± 14*	DXA (Hologic 2000) with HSA program (version 2.1) Hip regions (narrow neck, intertrochantic, femoral shaft) CSA (cm^2^), *Z* (cm^3^)	Gender-specific partial correlation coefficients were calculated between MPA and VPA and bone structural properties. Adjusted for age, mass and height Narrow neck: CSA: Boys: **MPA**: *r* = 0.09, *p* > 0.05; **VPA**: *r* = 0.32, *p* < 0.01; girls: **MPA**: *r* = 0.25, *p* < 0.01; **VPA**: *r* = 0.29, *p* < 0.01 Narrow neck: section modulus (*Z*): Boys: **MPA**: *r* = 0.06, *p* > 0.05; **VPA**: *r* = 0.28, *p* < 0.01; girls: **MPA**: *r* = 0.19, *p* < 0.01; **VPA**: *r* = 0.19, *p* < 0.01 Intertrochantic CSA: Boys: **MPA**: *r* = 0.02, *p* > 0.05; **VPA**: *r* = 0.32, *p* < 0.01; girls: **MPA**: *r* = 0.24, *p* < 0.01; **VPA**: *r* = 0.28, *p* < 0.01 Intertrochantic: section modulus (*Z*): Boys: **MPA**: *r* = 0.02, *p* > 0.05; **VPA**: *r* = 0.30, *p* < 0.01; girls: **MPA**: *r* = 0.24, *p* < 0.01; **VPA**: *r* = 0.27, *p* < 0.01 Femoral shaft CSA: Boys: **MPA**: *r* = 0.23, *p* < 0.01; **VPA**: *r* = 0.31, *p* < 0.01; girls: **MPA**: *r* = 0.18, *p* < 0.05; **VPA**: *r* = 0.24, *p* < 0.01 Femoral shaft: section modulus (*Z*): Boys: **MPA:** *r* = − 0.02, *p* > 0.05; **VPA**: *r* = 0.22, *p* < 0.01; girls: **MPA**: *r* = 0.22, *p* < 0.01; **VPA**: *r* = 0.24, *p* < 0.01
Sardinha et al. 2008 [55] Cross-sectional ◆◆	*n* = 293 (150/143) 9.7 ± 0.3 years **European Youth Heart Study** Pre- or early pubertal (Tanner stages 1 and 2)	WAM 6471 **60-s epoch** Location: right hip Worn for: 2 WK and 2 WE days (during day time, except WB activities) For inclusion: ≥ 3 days with ≥ 600 min Non-wear: NR	MPA, 2001–3999 cpm VPA, ≥ 4000 cpm (min/day) **MPA**: m, 169.4 ± 55.4; f, 141.7 ± 47.3* **VPA**: m, 29.4 ± 21.2; f, 18.0 ± 14.4*	DXA (QDR-1500, software 5.73 for TB, 4.76 for LS and FN) TB, LS, FN BMC (g), compressive strength (g/kg), bending strength (g/kg), impact strength (g/kg)	Partial correlation to assess the linear association between PA and bone variables (adjusted for age, body weight, FFM, bone area and body height for BMC variables, adjusted for age and FFM for FN strength variables) Stepwise multiple regression was used to determine the independent contribution of PA variables on the variance of BMC and FN structural variables (same adjustments as above) *B* = partial regression coefficient, *t* = student’s *t* test, *SR*^2^ = squared semipartial correlation TB BMC: Boys: **MPA**: *r* = 0.08, *p* > 0.05; **VPA**: *r* = 0.17, *p* < 0.05; *B* = 0.627, *t* = 1.994, *SR*^2^ (%) = 0.5, *p* = 0.048 Girls: **MPA**: *r* = 0.04, *p* > 0.05; **VPA**: *r* = 0.20, *p* < 0.05; *B* = 1.102, *t* = 2.360, *SR*^2^ (%) = 0.6, *p* = 0.020 LS BMC: Boys: **MPA**: *r* = 0.05, *p* > 0.05; **VPA**: *r* = 0.11, *p* > 0.05; girls: **MPA**: *r* = 0.03, *p* > 0.05; **VPA**: *r* = 0.05, *p* > 0.05 FN BMC: Boys: **MPA**: *r* = 0.11, *p* > 0.05; **VPA**: *r* = 0.23, *p* < 0.05; *B* = 0.003, *t* = 2.876, *SR*^2^ (%) = 2.8, *p* = 0.005 Girls: **MPA**: *r* = 0.14, *p* > 0.05; **VPA**: *r* = 0.20, *p* < 0.05; *B* = 0.003, *t* = 2.453, *SR*^2^ (%) = 1.3, *p* = 0.015 FN compressive strength: Boys: **MPA**: *r* = 0.22, *p* < 0.01; **VPA**: *r* = 0.29, *p* < 0.01; *B* = 0.012, *t* = 3.663, *SR*^2^ (%) = 6.3, *p* < 0.001 Girls: **MPA**: *r* = 0.12, *p* > 0.05; **VPA**: *r* = 0.25, *p* < 0.01; *B* = 0.015, *t* = 3.040, *SR*^2^ (%) = 5.7, *p* = 0.003 FN bending strength: Boys: **MPA**: *r* = 0.20, *p* < 0.05; *B* = 0.001, *t* = 2.418, *SR*^2^ (%) = 3.2, *p* = 0.017; **VPA**: *r* = 0.19, *p* < 0.05 Girls: **MPA**: *r* = 0.14, *p* > 0.05; **VPA**: *r* = 0.26, *p* < 0.01; *B* = 0.006, *t* = 3.252, *SR*^2^ (%) = 6.8, *p* = 0.001 FN impact strength: Boys: **MPA**: *r* = 0.23, *p* < 0.01; **VPA**: *r* = 0.34, *p* < 0.01; *B* = 0.001, *t* = 4.350, *SR*^2^ (%) = 9.3, *p* < 0.001 Girls: **MPA**: *r* = 0.10, *p* > 0.05; **VPA**: *r* = 0.22, *p* < 0.05; *B* = 0.001, *t* = 2.683, *SR*^2^ (%) = 4.7, *p* = 0.008
Specker et al. 2001^†^ [57] Cross-sectional ◆	*n* = 239 (124/115) m, 4.0 ± 0.6 years f, 3.9 ± 0.6 years NR	Actiwatch motion sensor **Epoch NR** (PA described in cpm in methods, **60 s** assumed) Location: lumbar spine Worn for: 2 days For inclusion: 2 days Non-wear: NR	MVPA, > 500 cpm VPA, > 1000 cpm Average % time: **MVPA**: m, 13.5 ± 3.9; f, 12.1 ± 4.2* **VPA**: m, 5.2 ± 2.2; f, 4.5 ± 2.2*	DXA (Hologic 4500A) TB BMC (g), BA (cm^2^)	To identify significant predictors of TB BA and TB BMC, a forward–backward stepwise regression was performed including variables that were significant (*p* ≤ 0.05) after including height or TB BA in models predicting TB BA or TB BMC. After obtaining final models, PA measures were added to assess whether they explained a significant amount of remaining error TB BA: **MVPA**: NS; **VPA**: NS TB BMC: **MVPA**: NS; **VPA**: NS
Tobias et al. 2007 [60] Cross-sectional ◆◆	*n* = 4457 (2121/2336) 11.8 ± 0.3 years **ALSPAC** Tanner stages (%) 1/2/3/4/5: m, 40/ 40/15/5/0 f, 10/30/35/20/5	MTI WAM 7164 **Epoch NR** (PA described in cpm in methods, so **60 s** assumed) Location: right hip Worn for: 7 days during waking (except WB activities) For inclusion: ≥ 3 days with ≥ 600 min Non-wear: NR	MPA, 3600–6199 cpm MVPA, ≥ 3600 cpm VPA, ≥ 6200 cpm (min/day): **MPA**: m, 24.6 ± 14.1; f, 15.5 ± 9.6* **MVPA**: m, 28.6 ± 17.0; f, 18.3 ± 11.6* **VPA**: m, 4.0 ± 4.3; f, 2.8 ± 3.7*	DXA (Lunar Prodigy, paediatric scanning software) TB, upper limbs, lower limbs BMC (g), BA (cm^2^), BMD (g/cm^2^), aBMC (g)	Multivariable regression analyses were used to examine the influence of different activity levels on bone outcomes. Fully adjusted models controlled for age of DXA scan, sex, socio-economic factors, height (both linear and quadratic terms), lean mass and fat mass. Light PA, MPA and VPA were included simultaneously. *β* represents the change in bone outcome per 100 cpm increase in activity levels Total body BMC: **MPA**: *β* = 18.83, *95% CI* 13.30, 24.36, *p* < 0.001; **VPA**: *β* = 6.59, *95% CI* 0.58, 24.12.60, *p* = 0.03 Area: **MPA**: *β* = 8.73, *95% CI* 4.90, 12.56, *p* < 0.001; **VPA**: *β* = 4.59, *95% CI* 0.42, 8.75, *p* = 0.03 BMD: **MPA**: *β* = 7.48, *95% CI* 5.36, 9.61, *p* < 0.001; **VPA**: *β* = 2.44, *95% CI* 0.13, 4.75, *p* = 0.04 aBMC: **MPA**: *β* = 8.09, *95% CI* 5.33, 10.84, *p* < 0.001; **VPA**: *β* = 0.94, *95% CI* − 2.05, 3.94, *p* = 0.5 Upper limb: BMC: **MPA**: *β* = 0.11, *95% CI* − 0.80, 1.02, *p* = 0.8; **VPA**: *β* = 1.34, *95% CI* 0.35, 2.34, *p* = 0.008 Area: **MPA**: *β* = − 0.06, *95% CI* − 1.01, 0.88, *p* = 0.9; **VPA**: *β* = 1.12, *95% CI* 0.09, 2.15, *p* = 0.003 BMD: **MPA**: *β* = 0.17, *95% CI* − 1.59, 1.93, *p* = 0.9; **VPA**: *β* = 2.83, *95% CI* 0.92, 4.75, *p* = 0.004 aBMC: **MPA**: *β* = 0.17, *95% CI* − 0.23, 0.57, *p* = 0.4; **VPA**: *β* = 0.36, *95% CI* − 0.08, 0.79, *p* = 0.1 Lower limb: BMC: **MPA**: *β* = 13.07, *95% CI* 10.60, 15.53, *p* < 0.001; **VPA**: *β* = 2.25, *95% CI* − 0.43, 4.94, *p* = 0.1 Area: **MPA**: *β* = 5.07, *95% CI* 3.72, 6.41, *p* < 0.001; **VPA**: *β* = 0.76, *95% CI* − 0.70, 2.23, *p* = 0.3 BMD: **MPA**: *β* = 12.61, *95% CI* 9.46, 15.77, *p* < 0.001; **VPA**: *β* = 2.91, *95% CI* − 0.52, 6.34, *p* = 0.1 aBMC: **MPA**: *β* = 5.3, *95% CI* 3.30, 7.30, *p* < 0.001; **VPA**: *β* = 1.08, *95% CI* − 1.08, 3.25, *p* = 0.3 Also did linear regression analysis between MVPA and bone outcomes. Same adjustments as above. *β* represents the change in bone outcome per 100 cpm increase in activity level TB: BMC: **MVPA**: *β* = 14.92, *95% CI* 11.80,18.05, *p* < 0.001; area: **MVPA**: *β* = 7.82, *95% CI* 5.65,9.98, *p* < 0.001; BMD: **MVPA**: *β* = 5.90, *95% CI* 4.70,7.11, *p* < 0.001; **MVPA**: aBMC: *β* = 5.30, *95% CI* 3.75, 6.86, *p* < 0.001 Upper limb: BMC: **MVPA**: *β* = 1.05, *95% CI* 0.53, 1.57, *p* < 0.001; area: **MVPA**: *β* = 0.81, *95% CI* 0.27, 1.34, *p* = 0.003 BMD: **MVPA**: *β* = 2.10, *95% CI* 1.11, 3.10, *p* < 0.001; aBMC: **MVPA**: *β* = 0.34, *95% CI* 0.11, 0.56, *p* = 0.003 Lower limb: BMC: **MVPA**: *β* = 9.44, *95% CI* 8.04, 10.84, *p* < 0.001; area: **MVPA**: *β* = 3.72, *95% CI* 2.95,4.48, *p* < 0.001; BMD: **MVPA**: *β* = 9.42, *95% CI* 7.63, 11.20, *p* < 0.001; aBMC: **MVPA**: *β* = 3.74, *95% CI* 2.62, 4.87, *p* < 0.001 Interactions: The association between MVPA and TB bone area was stronger in boys than girls (*β* = 9.62 and 5.21, respectively, *p* for interaction = 0.04, adjusted for height, lean mass, fat mass). The + ve association between total MVPA and UL aBMC was attenuated by puberty (subgroup of 2589 children with Tanner stage data; *β* = 0.66, 0.62, − 0.39, 0.42 and − 2.13 for Tanner stages 1, 2, 3, 4 and 5. *p* for interaction = 0.01, adjusted for height, LM, FM)
Torres-Costoso et al. 2015 [61] Cross-sectional ◆	*n* = 132 (62/70) 9.43 ± 0.72 years NR	MTI 7164 **60-s epoch** Location: right hip Worn for: 7 days (except during WB activity) For inclusion: ≥ 4 days (1 WE) with ≥ 10 h Non-wear: ≥ 10 consecutive 0 counts	MVPA, ≥ 2296 cpm VPA, ≥ 4012 cpm (min/day): **MVPA**: m, 55.75 ± 25.27; f, 39.61 ± 19.44* ALL, 46.88 ± 23.57 **VPA**: m, 10.65 ± 8.52; f, 8.15 ± 6.12 ALL, 9.28 ± 7.37	DXA (LunariDXA) TB BMC (g)	Partial correlation coefficients (controlling for age) were used to assess the relationships between PA variables (MVPA and VPA) and total body BMC **MVPA**: *r* = − 0.110, *p* > 0.05; **VPA**: *r* = − 0.208, *p* < 0.05
Cardadeiro et al. 2014a [41] Cross-sectional ◆◆	*n* = 294 (139/146) 10.3 ± 0.5 years Maturity offset: m, − 2.87 ± 0.5 years; f, − 1.19 ± 0.6 years	Actigraph GT1M **15-s epoch** Location: right hip Worn for: 2 WK and 2 WE days (except WB activities) For inclusion: ≥ 4 days with ≥ 600 min/day Non-wear: NR	MPA, 2296–4011 cpm MVPA, ≥ 2296 cpm VPA, ≥ 4012 cpm (min/day) **MPA**: m, 34.9 ± 13; f, 27.9 ± 9.6* **MVPA**: m, 50.3 ± 21; f, 39.1 ± 14* **VPA**: m, 15.5 ± 9; f, 11.2 ± 7.0*	DXA (QDR Explorer, Hologic Inc.) PF subregions: FN, TR, IT BMD (g/cm^2^)	Stepwise linear regression was used to analyse the associations between PA and PF (Neck, TR, IT) BMD. Adjusted for maturity, body height and lean body mass Femoral neck BMD: Boys: **MPA**: Std *β* = − 0.116, *p* = 0.668; **MVPA**: Std *β* = 0.393, *R*^2^ = 0.155, *p* < 0.001; **VPA**: Std *β* = 0.085, *p* = 0.430 Girls: **MPA**: Std *β* = 0.050, *p* = 0.753; **MVPA**: Std *β* = 0.211, *R*^2^ = 0.044, *p* = 0.001; **VPA**: Std *β* = − 0.037, *p* = 0.753 Trochanter BMD: Boys: **MPA**: Std *β* = − 0.257, *p* = 0.369; **MVPA**: Std *β* = 0.387, *R*^2^ = 0.087, *p* < 0.001; **VPA**: Std *β* = 0.187, *p* = 0.369 Girls: **MPA**: Std *β* = 0.056, *p* = 0.479; **MVPA**: Std *β* = 0.088, *p* = 0.479; **VPA**: Std *β* = 0.161, *R*^2^ = 0.021, *p* = 0.011 Intertrochanter BMD: Boys: **MPA**: Std *β* = − 0.129, *p* = 0.616; **MVPA**: Std *β* = 0.374, *R*^2^ = 0.136, *p* < 0.001; **VPA**: Std *β* = 0.094, *p* = 0.616 Girls: **MPA**: Std *β* = 0.142, *R*^2^ = 0.020, *p* = 0.035; **MVPA**: Std *β* = 0.020, *p* = 0.909; **VPA**: Std *β* = 0.010, *p* = 0.909
Gracia-Marco et al. 2011 [47] Cross-sectional ◆◆	*n* = 380 (189/191) Subjects classified as non-active (< 60 min/day MVPA) and active (≥ 60 min/day) m < 60 min, 14.9 ± 1.2 years; ≥ 60 min, 14.6 ± 1.3 years f < 60 min, 14.7 ± 1.1 years; ≥ 60 min, 15.1 ± 1.2 years **HELENA study** Tanner stage 1/2/3/4/5 (%): m, < 60 min: 0/4/8/18/70 m, ≥ 60 min: 0/3/12/22/63 f, < 60 min: 0/1/6/4/89 f, ≥ 60 min: 0/0/3/12/85	Actigraph GT1M **15-s epoch** Location: lower back Worn for: 7 days (except WB activities) For inclusion: ≥ 3 days with ≥ 8 h/day Non-wear: NR	MVPA, ≥ 2000 cpm VPA, ≥ 4000 cpm (min/day): < 60 min MVPA/day: **MVPA**: m, 45 ± 11; f, 40 ± 11* **VPA**: m, 14 ± 7; f, 10 ± 6* ≥ 60 min MVPA/day: **MVPA**: m, 82 ± 20; f, 77 ± 15 **VPA**: m, 33 ± 13; f, 27 ± 12	DXA (Hologic explorer, paediatric version of QDR-explorer software v 12.4) TB, hip, LS, TR, IT, FN BMC (g), BMD (g/cm^2^)	Receiver operating characteristic (ROC) curve analysis was used to assess relationships between PA and bone mass. BMD and BMC z-scores were calculated and participants were grouped based on this. Results from the high group (+ 2 SD BMC and BMD) are presented. AUC, area under the curve; cut-off points were selected for scores optimising sensitivity–specificity relationship. ROC curve indexes of each cut-off point were calculated through determination of + ve (*PPV*) and –ve (*NPV*) predictive values, overall misclassification rate (OMR), + ve (PLR) and –ve (NLR) likelihood ratios and Youden index (A) TB BMC: **MVPA**: min/day = 41; *AUC* (*CI*) = 0.562 (0.419, 0.705), sensitivity = 0.909, specificity = 0.270, *p* > 0.05; **VPA**: min/day = 23; *AUC* (*CI*) = 0.609 (0.447, 0.771), sensitivity = 0.545, specificity = 0.668, *p* > 0.05 TB BMD: **MVPA**: min/day = 44; *AUC* (*CI*) = 0.566 (0.373, 0.76), sensitivity = 0.889, specificity = 0.331, *p* > 0.05; **VPA**: min/day = 38; *AUC* (*CI*) = 0.567 (0.349, 0.786), sensitivity = 0.333, specificity = 0.898, *p* > 0.05 Hip BMC: **MVPA**: min/day = 57; *AUC* (*CI*) = 0.643 (0.501, 0.786), sensitivity = 0.778, specificity = 0.537, *p* > 0.05; **VPA**: min/day = 19; *AUC* (*CI*) = 0.692 (0.557, 0.828), sensitivity = 0.583, specificity = 0.608, *OMR* (%) = 60.77, *PPV* (%) = 4.52, *NPV* (%) = 97.87, *PLR* = 1.49, *NLR* = 0.68, *A* = 0.19, *p* < 0.05 Hip BMD: **MVPA**: min/day = 78; *AUC* (*CI*) = 0.673 (0.497, 0.848), sensitivity = 0.500, specificity = 0.824, *p* > 0.05; **VPA**: min/day = 28; *AUC* (*CI*) = 0.802 (0.666, 0.937), sensitivity = 0.667, specificity = 0.794, *OMR* (%) = 79.23, *PPV* (%) = 4.82, *NPV* (%) = 99.35, *PLR* = 3.24, *NLR* = 0.42, *A* = 0.46, *p* ≤ 0.01 LS BMC: **MVPA**: min/day = 73; *AUC* (*CI*) = 0.581 (0.392, 0.770), sensitivity = 0.545, specificity = 0.759, *p* > 0.05; **VPA**: min/day = 22; *AUC* (*CI*) = 0.632 (0.435, 0.829), sensitivity = 0.727, specificity = 0.648, *p* > 0.05 LS BMD: **MVPA**: min/day = 82; *AUC* (*CI*) = 0.442 (0.168, 0.717), sensitivity = 0.333, specificity = 0.849, *p* > 0.05;**VPA**: min/day = 38; *AUC* (*CI*) = 0.477 (0.188, 0.765), sensitivity = 0.333, specificity = 0.896, *p* > 0.05 Trochanter BMC: **MVPA**: min/day = 41; *AUC* (*CI*) = 0.591 (0.45, 0.732), sensitivity = 0.909, specificity = 0.290, *p* > 0.05;**VPA**: min/day = 28; *AUC* (*CI*) = 0.665 (0.513, 0.818), sensitivity = 0.545, specificity = 0.778, *p* > 0.05 Trochanter BMD: **MVPA**: min/day = 53; *AUC* (*C*I) = 0.475 (0.293, 0.657), sensitivity = 0.600, specificity = 0.479, *p* > 0.05; **VPA**: min/day = 24; *AUC* (*CI*) = 0.514 (0.309, 0.719), sensitivity = 0.500, specificity = 0.683, *p* > 0.05 Intertrochanter BMC: **MVPA**: min/day = 57; *AUC* (*CI*) = 0.541 (0.369, 0.714), sensitivity = 0.667, specificity = 0.534, *p* > 0.05; **VPA**: min/day = 19; *AUC* (*CI*) = 0.576 (0.411, 0.741), sensitivity = 0.777, specificity = 0.574, *p* > 0.05 Intertrochanter BMD: **MVPA**: min/day = 46; *AUC* (*CI*) = 0.664 (0.509, 0.818), sensitivity = 0.889, specificity = 0.263, *p* > 0.05; **VPA**: min/day = 28; *AUC* (*CI*) = 0.741 (0.578, 0.908), sensitivity = 0.556, specificity = 0.795, *OMR* (%) = 78.97, *PPV* (%) = 6.02, *NPV* (%) = 98.70, *PLR* = 2.71, *NLR* = 0.56, *A* = 0.35, *p* ≤ 0.01 Femoral neck BMC: **MVPA**: min/day = 78; *AUC* (*CI*) = 0.544 (0.340, 0.747), sensitivity = 0.444, specificity = 0.825, *p* > 0.05; **VPA**: min/day = 27; *AUC* (*CI*) = 0.608 (0.408, 0.809), sensitivity = 0.555, specificity = 0.757, *p* > 0.05 Femoral neck BMD: **MVPA**: min/day = 78; *AUC* (*CI*) = 0.835 (0.735, 0.936), sensitivity = 0.643, specificity = 0.586, *OMR* (%) = 63.33, *PPV* (%) = 87.66, *NPV* (%) = 26.45, *PLR* = 1.55, *NLR* = 0.61, *A* = 0.23, *p* ≤ 0.01 **VPA**: min/day = 32; *AUC* (*CI*) = 0.889 (0.813, 0.964), sensitivity = 0.667, specificity = 0.846, *OMR* (%) = 84.36, *PPV* (%) = 6.35, *NPV* (%) = 99.39, *PLR* = 4.34, *NLR* = 0.39, *A* = 0.51, *p* ≤ 0.01
Marin-Puyalto et al. 2019 [52] Cross-sectional ◆◆	*n* = 180 (boys only) 12.07 ± 0.69 years Bone age 11.87 ± 1.06 years	Actigraph GT1M **15-s epoch** Location: right hip Worn for: 7 days For inclusion: ≥ 3 days (≥ 1WE) with ≥ 10-h recording Non-wear: ≥ 20 min consecutive 0 counts	MPA, ≥ 2296 cpm VPA, ≥ 4012 cpm (min/day): **MPA,** 40.3 ± 14.2 **VPA,** 19.5 ± 13.6	DXA (DPX-IQ, Lunar Corporation) TB, LS, FN BMC (g), BMD (g/cm^2^)	Partial Pearson’s correlation coefficients (adjusted for body mass and skeletal age) were used to assess the relationships amongst bone and PA parameters TB BMC: **MPA**: *r* = 0.094, *p* > 0.05; **VPA**: *r* = 0.234, *p* < 0.05 TB BMD: **MPA**: *r* = 0.087, *p* > 0.05; **VPA**: *r* = 0.141, *p* > 0.05 FN BMC: **MPA**: *r* = 0.254, *p* < 0.05; **VPA**: *r* = 0.364, *p* < 0.05 FN BMD: **MPA**: *r* = 0.238, *p* < 0.05; **VPA**: *r* = 0.317, *p* < 0.05 LS BMC: **MPA**: *r* = 0.065, *p* > 0.05; **VPA**: *r* = 0.201, *p* < 0.05 LS BMD: **MPA**: *r* = 0.000, *p* > 0.05; **VPA**: *r* = 0.096, *p* > 0.05
Meiring et al. 2014^†^ [39] Cross-sectional ◆◆	*n* = 38 (12/26) 9.9 ± 1.3 years Tanner stages 1/2 (*n*): 22/16	Actical accelerometer **15-s epoch** Location: right hip Worn for: 7 days (except WB activity) For inclusion: ≥ 4 days with ≥ 10 h consecutive counts Non-wear: only full days of non-wear removed (full days consecutive 0 counts or SR removal)	MPA, 1500–6500 cpm MVPA, > 1500 cpm VPA, > 6501 cpm (min/day): **MPA**, 55.6 ± 23.5 **MVPA**, NR **VPA**, 2.1 ± 3.0	DXA (Hologic, QDR, Discovery) WB, LS, hip, FN, forearm (ulna and radius) BMC (g)	Pearson’s correlation was performed between MPA, MVPA and VPA. DXA measurements adjusted for bone area, body mass and sex BMC: Ulna: **MPA**: *r* = 0.07, *p* > 0.05; **MVPA**: *r* = 0.03, *p* > 0.05; **VPA**: *r* = 0.15, *p* > 0.05 Radius: **MPA**: *r* = − 0.03, *p* > 0.05; **MVPA**: *r* = − 0.05, *p* > 0.05; **VPA**: *r* = 0.08, *p* > 0.05 Spine: **MPA**: *r* = 0.17, *p* > 0.05; **MVPA**: *r* = 0.15, *p* > 0.05; **VPA**: *r* = 0.30, *p* > 0.05 Whole body: **MPA**: *r* = 0.10, *p* > 0.05; **MVPA**: *r* = 0.09, *p* > 0.05; **VPA**: *r* = 0.25, *p* > 0.05 Hip: **MPA**: *r* = 0.26, *p* > 0.05; **MVPA**: *r* = 0.26, *p* > 0.05; **VPA**: *r* = 0.36, *p* < 0.05 Femoral neck: **MPA**: *r* = 0.34, *p* < 0.05; **MVPA**: *r* = 0.32, *p* < 0.05; **VPA**: *r* = 0.30, *p* > 0.05
Sioen et al. 2015 [56] Cross-sectional ◆◆	*n* = 210 (105/105) 9.8 ± 1.4 years **ChiBS study** Based on Tanner score: No sign of puberty, 71% Signs of puberty, 29%	Actigraph GT3X, GT1M or Actitrainer **15-s epoch** Location: right hip Worn for: 5 days (WK and WE) during waking hours (except WB activities or high-risk sports) For inclusion: ≥ 3 days with ≥ 8 but ≤ 18 h Non-wear: ≥ 20 min consecutive 0 counts	MPA, 2296–4011 cpm MVPA, ≥ 2296 cpm VPA, ≥ 4012 cpm % of recorded time: **MPA**: m, 5.7 ± 2.0; f, 4.4 ± 1.5* ALL, 5.0 ± 1.9 **MVPA**: m, 8.7 ± 3.5; f, 6.7 ± 2.9* ALL, 7.7 ± 3.3 **VPA**: m, 2.9 ± 1.8; f, 2.4 ± 1.7* ALL, 2.7 ± 1.8	DXA (Hologic Discovery-W apparatus, software version 12.8.0) TB BMC (g), aBMD (g/cm^2^)	Multiple linear regression was used to examine associations between PA and bone outcomes (adjusted for age, gender, Tanner stage, height, fat mass index and fat-free mass index) TB BMC: **MPA**: std *β* = − 0.006, *p* = 0.838, eta^2^ = 0.000; **MVPA**: std *β* = − 0.009, *p* = 0.755, eta^2^ = 0.0001; **VPA**: std *β* = − 0.008, *p* = 0.752, eta^2^ = 0.0001 TB aBMD: **MPA**: std *β* = 0.018, *p* = 0.607, eta^2^ = 0.002; **MVPA**: std *β* = 0.033, *p* = 0.328, eta^2^ = 0.001; **VPA**: std *β* = 0.041, *p* = 0.196, eta^2^ = 0.002
Hasselstrøm et al. 2007 [48] Cross-sectional ◆◆	*n* = 562 (297/265) m, 6.81 ± 0.37 years f, 6.66 ± 0.35 years **CoSCIS** NR	MTI 7164 **10-s epoch** Location: lower back Worn for: 2 WK and 2 WE days (except WB activities) For inclusion: ≥ 3 days with > 8 h per day Non-wear: ≥ 10 min consecutive 0 counts	Computed threshold counts for different intervals: ≥ 3000, ≥ 4000, ≥ 5200, ≥ 6500, ≥ 7000, ≥ 8200 cpm % of total time spent in: ** ≥ 3000 cpm**: m, 6.98 ± 2.74; f, 5.81 ± 1.98* ** ≥ 4000 cpm**: m, 3.90 ± 1.85; f, 3.16 ± 1.30* ** ≥ 5200 cpm**: m, 2.04 ± 1.19; f, 1.66 ± 0.80* ** ≥ 6500 cpm**: m, 1.09 ± 0.84; f, 0.92 ± 0.53* ** ≥ 7000 cpm**: m, 0.89 ± 0.76; f, 0.76 ± 0.47* ** ≥ 8200 cpm**: m, 0.61 ± 0.63; f, 0.52 ± 0.371*	Peripheral DXA (Lunar PIXI, software v. 1.4 CDMDD) Calcaneus, distal forearm BMC (g), BMD (g/cm^2^)	Univariate linear regression was conducted between calcaneal and distal forearm BMD and PA intensities. Analyses were adjusted for age, weight and height^2^ (present partial correlation coefficients) Calcaneal BMD: ** ≥ 3000 cpm**: *β* = 0.003, *95% CI* for *β* = 0.0016–0.0042, *p* < 0.001; ≥ **4000 cpm**: *β* = 0.005, *95% CI* for *β* = 0.0031–0.0070, *p* < 0.001; **5200 cpm**: *β* = 0.008, *95% CI* for *β* = 0.0051–0.0113, *p* < 0.001; ≥ **6500 cpm**: *β* = 0.009, *95% CI* for *β* = 0.0045–0.0137, *p* < 0.001; ≥ **7000 cpm**: *β* = 0.009, *95% CI* for *β* = 0.0036–0.0139, *p* < 0.05; ≥ **8200 cpm**: *β* = 0.007, *95% CI* for *β* = 0.0011–0.0137, *p* < 0.05 Distal forearm BMD: ** ≥ 3000 cpm**: *β* = 0.002, *95% CI* for *β* = 0.0013–0.0033, *p* < 0.001; ≥ **4000 cpm**: *β* = 0.003, *95% CI* for *β* = 0.0018–0.0048, *p* < 0.001; **5200 cpm**: *β* = 0.005, *95% CI* for *β* = 0.0022–0.0069, *p* < 0.001; ≥ **6500 cpm**: *β* = 0.005, *95% CI* for *β* = 0.0018–0.0087, *p* < 0.05; ≥ **7000 cpm**: *β* = 0.005, *95% CI* for *β* = 0.0014–0.0091, *p* < 0.05; ≥ **8200 cpm**: *β* = 0.005, *95% CI* for *β* = 0.0004–0.0098, *p* < 0.05
McCormack et al. 2016 [53] Cross-sectional ◆◆	*n* = 87 (44/43) m, 10.1 ± 0.9 years f, 10.0 ± 0.8 years NR	Actigraph GT3X + **10-s epoch** (from raw) Location: right hip Worn for: 7 days (during 07:30–21:30, except WB activities) For inclusion: ≥ 10 h per day for ≥ 3WK and 1 WE day Non-wear: ≥ 60 min consecutive 0 counts	Age-appropriate Evenson cut-points were linearly scaled to accommodate 10-s epochs (min/day): **MPA**: m, 44 ± 13; f, 32 ± 10* **MVPA**: m, 71 ± 25; f, 51 ± 22* **VPA**: m, 28 ± 15; f, 19 ± 14*	DXA (Discovery, Hologic Inc., Pediatric Whole Body Analysis Method, software Apex 3.0) TB BMC (g)	Multiple regression was used to test the effect of each activity level on TB BMC. Adjusted for age, sex and height TB BMC: **MPA**: *β* = 1.8, *95% CI* (0.2 to 3.4), *p* < 0.05; **MVPA**: *β* = 0.70, *95% C* (− 0.10 to 1.49), *p* > 0.05; **VPA**: *β* = 0.70, *95% C* (− 0.62 to 2.02), *p* > 0.05
Bielemann et al. 2019 [40] Cross-sectional^††^ ◆◆	*n* = 3556 (1744/1812) 18 years **1993 Pelotas (Brazil) Birth Cohort** NR	GeneActiv **5-s epoch** (from raw) Location: non-dominant wrist Worn for: all day and night, period of use was 4–7 days (1 WE) For inclusion: ≥ 4 days (1 WE). Valid day NR Non-wear: NR	MPA, 100 mg VPA, 400 mg (min/day): **MPA**: Tertile 1: m, 94.1 ± 25.3; f, 84.5 ± 19.1 Tertile 2: m, 157.8 ± 17.0; f, 128.8 ± 11.5 Tertile 3: m, 241.0 ± 44.8; f, 191.6 ± 40.7 **VPA**: Tertile 1: m, 4.4 ± 2.1; f, 2.0 ± 1.0 Tertile 2: m, 12.3 ± 2.6; f, 5.7 ± 1.2 Tertile 3: m, 29.6 ± 12.7; f, 15.7 ± 8.4	DXA (Lunar Prodigy Advance) FN, LS aBMD (g/cm^2^)	Linear regression was used to assess associations between MPA and VPA (stratified into 1st, 2nd and 3rd tertiles) and bone outcomes (only at 18 years as this was the only time point with accelerometer PA data). Analyses were adjusted for skin colour, asset index at 11 years and current height Lumbar spine aBMD: Boys: **MPA**: *p* = 0.181, 1st = Ref.; 2nd: *β* coef. (*95% CI*) = 0.01 (0.00; 0.03); 3rd: *β* coef. (*95% CI*) = 0.01 (0.00; 0.03); **VPA**: *p* < 0.001, 1st = Ref.; 2nd: *β* coef. (*95% CI*) = 0.03 (0.01; 0.04); 3rd: *β* coef. (*95% CI*) = 0.04 (0.02; 0.05) Girls: **MPA**: *p* = 0.020, 1st = Ref.; 2nd: *β* coef. (*95% CI*) = 0.00 (− 0.02; 0.01); 3rd: *β* coef. (*95% CI*) = 0.02 (0.00; 0.03); **VPA**: *p* = 0.089, 1st = Ref.; 2nd: *β* coef. (*95% CI*) = 0.00 (− 0.01; 0.02); 3rd: *β* coef. (*95% CI*) = 0.02 (0.00; 0.03) Femoral neck aBMD: Boys: **MPA**: *p* = 0.001, 1st = Ref.; 2nd: *β* coef. (*95% CI*) = 0.03 (0.01; 0.05); 3rd: *β* coef.(*95% CI*) = 0.03 (0.01; 0.05);**VPA**: *p* < 0.001, 1st = Ref.; 2nd: *β* coef. (*95% CI*) = 0.04 (0.02; 0.06); 3rd: *β* coef. (*95% CI*) = 0.06 (0.05; 0.08) Girls: **MPA**: *p* < 0.001, 1st = Ref.; 2nd: *β* coef. (*95% CI*) = 0.01 (− 0.01; 0.02); 3rd: *β* coef. (*95% CI*) = 0.03 (0.02; 0.05);**VPA**: *p* = 0.150, 1st = Ref.; 2nd: *β* coef. (*95% CI*) = 0.00 (− 0.01; 0.02); 3rd: *β* coef. (*95% CI*) = 0.01 (0.00; 0.03)
Deere et al. 2012a [45] Cross-sectional ◆◆	*n* = 724 (295/429) m, 17.7 ± 0.27 years f, 17.7 ± 0.30 years **ALSPAC** NR	Newtest monitor **Raw data** Location: NR Worn for: 7 days (during waking hours, except WB activity) For inclusion: ≥ 2 days with ≥ 8-h recording Non-wear: kept diary of monitor wear	6 impact bands: (1) 0.5–1.1 g, (2) 1.1–2.1 g, (3) 2.1–3.1 g, (4) 3.1–4.2 g, (5) 4.2–5.1 g and (6) > 5.1 g median no. of impacts/day: **0.5 to 1.1 g**: m, 3600; f, 3121 **1.1 to 2.1 g**: m, 755; f, 596.6 **2.1 to 3.1 g**: m, 107; f, 83.6 **3.1 to 4.2 g**: m, 33.2; f, 25.0 **4.2 to 5.1 g**: m, 10.1; f, 7.8 ** > 5.1 g**: m, 13.3; f, 8.6	DXA (GE Lunar Prodigy; manufacturers automated advance hip analysis software) Total hip, FN BMD (g/cm^2^), FN width (mm), cortical CSA (mm^2^), cortical thickness (mm), buckling ratio, CSMI (cm^4^), section modulus (mm^3^)	Regression analysis assessed associations between the no of counts/day within each impact band and bone outcomes. A nonparametric bootstrap sampled with replacement based on 1000 replicates was used to generate *β* coefficients with *95% CI*’s. Activity data was log transformed. FN BMD fully adjusted models adjusted for age, height, gender, fat mass, lean mass and social position; fully adjusted models for FN width, CT, CSMI and hip BMD adjusted for age, height, gender, fat mass and lean mas. *β* represents change in SD per doubling in activity FN: BMD: **Band 1**: *β* = − 0.009, *95% CI* (− 0.075, 0.057), *p* = 0.781; **band 2**: *β* = 0.000, *95% CI* (− 0.055, 0.054), *p* = 0.996; **Band 3**: *β* = 0.031, *95% CI* (− 0.018, 0.080), *p* = 0.208; **band 4**: *β* = 0.043, *95% CI* (− 0.006, 0.091), *p* = 0.073; **Band 5**: *β* = 0.047, *95% CI* (0.001, 0.092), *p* = 0.039; **band 6**: *β* = 0.060, *95% CI* (0.010, 0.112), *p* = 0.017 Width: **Band 1:** *β* = 0.024, *95% CI* (− 0.020, 0.064), *p* = 0.261; **band 2**: *β* = 0.008, *95% CI* (− 0.024, 0.037), *p* = 0.593; **Band 3**: *β* = 0.024, *95% CI* (− 0.008, 0.056), *p* = 0.148; **band 4**: *β* = 0.031, *95% C*I (− 0.003, 0.068), *p* = 0.072; **Band 5**: *β* = 0.031, *95% CI* (0.002, 0.064), *p* = 0.052; **band 6**: *β* = 0.039, *95% CI* (0.004, 0.081), *p* = 0.047 Cortical thickness: **Band 1:** *β* = − 0.001, *95% CI* (− 0.066, 0.065), *p* = 0.968; **band 2**: *β* = 0.007, *95% CI* (− 0.048, 0.063), *p* = 0.812; **Band 3**: *β* = 0.027, *95% CI* (− 0.019, 0.071), *p* = 0.257; **band 4**: *β* = 0.035, *95% CI* (− 0.009, 0.076), *p* = 0.123; **Band 5**: *β* = 0.041, *95% CI* (− 0.001, 0.082), *p* = 0.056; **band 6**: *β* = 0.042, *95% CI* (0.003, 0.084), *p* = 0.062 CSMI: **Band 1:** *β* = 0.029, *95% CI* (− 0.011, 0.071), *p* = 0.160; **band 2**: *β* = 0.014, *95% CI* (− 0.019, 0.047), *p* = 0.398; **Band 3**: *β* = 0.022, *95% CI* (− 0.009, 0.052), *p* = 0.141; **band 4**: *β* = 0.030, *95% CI* (0.002, 0.060), *p* = 0.035; **Band 5**: *β* = 0.034, *95% CI* (0.009, 0.060), *p* = 0.009; **band 6**: *β* = 0.036, *95% CI* (0.008, 0.064), *p* = 0.014 Total hip: BMD: **Band 1**: *β* = − 0.009, *95% CI* (− 0.071, 0.053), *p* = 0.775; **band 2**: *β* = − 0.005, *95% CI* (− 0.057, 0.047), *p* = 0.856; **Band 3**: *β* = 0.023, *95% CI* (− 0.018, 0.069), *p* = 0.304; **band 4**: *β* = 0.031, *95% CI* (− 0.009, 0.071), *p* = 0.138 **Band 5**: *β* = 0.038, *95% CI* (0.000, 0.075), *p* = 0.048; **band 6**: *β* = 0.044, *95% CI* (0.003, 0.086), *p* = 0.034
Munoz-Hernandez et al. 2018 [54] Cross-sectional ◆◆	*n* = 177 (97/80) Overweight/obese High adherence to MDP: *n* = 31 (12/19) 10.2 ± 1.2 years Low adherence to MDP: *n* = 146 (85/61) 10.4 ± 1.2 years Pooled BL data from 2 RCTs: **The EFIGRO and the Active Brains Project** Puberty stages 1–3: *n* = 126 Puberty stages 4–5: *n* = 47	Actigraph GT3X + **Epoch NR** Location: non-dominant wrist Worn for: 7 days (except WB activity) For inclusion: ≥ 10 waking hours and ≥ 4 sleeping hours per day for ≥ 4 days (incl. 1 WE day) Non-wear: diary of wear/non-wear	Age-specific cut-points for ENMO (Hildebrand et al. 2014) PA displayed graphically	DXA (Hologic Discovery QDR series and QDR 4500 W) TB, UL, LL BMC (g) aBMD (g/cm^2^)	Regression analyses were conducted to assess the associations between PA levels and BMC and BMD. Adjusted for age, sex, study centre, height, lean mass and total energy intake. Analyses were conducted in boys and girls together stratified by adherence to the Mediterranean diet pattern (MDP; low and high) Total body aBMD: High adherence to MDP: **MPA**: std *β* = 0.115, *95% CI* = − 0.001; 0.002, *p* = 0.52; **MVPA**: std *β* = 0.084, *95% CI* = − 0.001; 0.001, *p* = 0.63; **VPA**: std *β* = − 0.018, *95% CI* = − 0.004; 0.003, *p* = 0.911 Low adherence to MDP: **MPA**: std *β* = 0.169, *95% CI* = − 0.004; 0.001, *p* = 0.004; **MVPA**: std *β* = 0.185, *95% CI* = 0.000; 0.001, *p* = 0.002; **VPA**: std *β* = 0.183, *95% CI* = 0.001; 0.004, *p* = 0.002 Total body BMC: High adherence to MDP: **MPA**: std *β* = 0.175, *95% CI* = − 0.512; 4.112, *p* = 0.121; **MVPA**: std *β* = 0.146, *95% CI* = − 0.642; 3.088, *p* = 0.188; **VPA**: std *β* = 0.037, *95% CI* = − 6.265; 8.928, *p* = 0.720 Low adherence to MDP: **MPA**: std *β* = 0.100, *95% CI* = 0.322; 1.998, *p* = 0.007; **MVPA**: std *β* = 0.109, *95% CI* = 0.355; 1.754, *p* = 0.003; **VPA**: std *β* = 0.108, *95% CI* = 1.509; 7.637, *p* = 0.004 Upper limbs aBMD: High adherence to MDP: **MPA**: std *β* = 0.087, *95% CI* = − 0.001; 0.001, *p* = 0.651; **MVPA**: std *β* = 0.051, *95% CI* = − 0.001; 0.001, *p* = 0.784; **VPA**: std *β* = − 0.057, *95% CI* = − 0.004; 0.003, *p* = 0.739 Low adherence to MDP: **MPA**: std *β* = 0.203, *95% CI* = 0.000; 0.001, *p* = 0.007; **MVPA**: std *β* = 0.204, *95% CI* = 0.000; 0.001, *p* = 0.007; **VPA**: std *β* = 0.142, *95% CI* = 0.00; 0.003, *p* = 0.062 Upper limbs BMC: High adherence to MDP: **MPA**: std *β* = 0.063, *95% CI* = − 0.215; 0.341, *p* = 0.642; **MVPA**: std *β* = 0.026, *95% CI* = − 0.200; 0.243, *p* = 0.844; **VPA**: std *β* = − 0.82, *95% CI* = − 1.150; 0.576, *p* = 0.498 Low adherence to MDP: **MPA**: std *β* = 0.159, *95% CI* = 0.063; 0.269, *p* = 0.002; **MVPA**: std *β* = 0.159, *95% CI* = 0.05; 0.223, *p* = 0.002; **VPA**: std *β* = 0.098, *95% CI* = − 0.013; 0.760, *p* = 0.058 Lower limbs aBMD: High adherence to MDP: **MPA**: std *β* = − 0.001, *95% CI* = − 0.001; 0.001, *p* = 0.997; **MVPA**: std *β* = − 0.012, *95% CI* = − 0.001; 0.001, *p* = 0.93; **VPA**: std *β* = − 0.040, *95% CI* = − 0.005; 0.003, *p* = 0.768 Low adherence to MDP: **MPA**: std *β* = 0.123, *95% CI* = 0.000; 0.001, *p* = 0.027; **MVPA**: std *β* = 0.142, *95% CI* = 0.000; 0.001, *p* = 0.011; **VPA**: std *β* = 0.167, *95% CI* = 0.001; 0.005, *p* = 0.002 Lower limbs BMC: High adherence to MDP: **MPA**: std *β* = 0.205, *95% CI* = − 0.127; 1.306, *p* = 0.102; **MVPA**: std *β* = 0.176, *95% CI* = − 0.158; 0.995, *p* = 0.146; **VPA**: std *β* = 0.071, *95% CI* = − 1.637; 3.070, *p* = 0.535 Low adherence to MDP: **MPA**: std *β* = 0.102, *95% CI* = 0.083; 0.573, *p* = 0.009; **MVPA**: std *β* = 0.112, *95% CI* = 0.096; 0.504, *p* = 0.004; **VPA**: std *β* = 0.113, *95% CI* = 0.431; 2.220, *p* = 0.004
Ivuškāns et al. 2015 [67] Longitudinal prospective ◆◆◆	*n* = 169 (boys only) BL, 12.06 ± 0.71 years 1-year FU, 13.06 ± 0.72 years Tanner stage 1/2/3/4/5 (*n*): BL, 0/64/89/16/0 FU, 0/20/82/48/19	Actigraph GT1M **60-s epoch** Location: right hip Worn for: 7 days (during waking) For inclusion: ≥ 8 h per day for ≥ 2WK and 1 WE day Non-wear: all night activity (00:00–06:00 h) and sequences of ≥ 10 min consecutive 0 counts	MPA, 2000–3999 cpm MVPA, ≥ 2000 cpm VPA, ≥ 4000 cpm (min/day): Baseline: **MPA**, 46.1 ± 19.4 **MVPA**, 57.6 ± 27 **VPA**, 11.4 ± 11.5 Follow-up: **MPA**, 41.4 ± 18.6* **MVPA**, 55.3 ± 27.2 **VPA**, 13.9 ± 12.4*	DXA (Lunar DPX-IQ) TB, LS, FN BMC (g), BMD (g/cm^2^), BA (cm^2^)	Partial correlation was used to assess the relationships between changes in BMD and BMC values during the 12-month study period with changes in PA variables (adjusted for Δ age, Δ pubertal status, Δ body mass) Δ TB BMD: **ΔMPA**: *r* = 0.080, *p* > 0.05; **ΔMVPA**: *r* = 0.111, *p* > 0.05; **ΔVPA**: *r* = 0.109, *p* > 0.05 Δ TB BMC: **ΔMPA**: *r* = − 0.057, *p* > 0.05; **ΔMVPA**: *r* = − 0.005, *p* > 0.05; **ΔVPA**: *r* = 0.074, *p* > 0.05 Δ TB BA: **ΔMPA**: *r* = − 0.053, *p* > 0.05; **ΔMVPA**: *r* = − 0.147, *p* > 0.05; **ΔVPA**: *r* = − 0.001, *p* > 0.05 Δ LS BMD: **ΔMPA**: *r* = 0.044, *p* > 0.05; **ΔMVPA**: *r* = 0.060, *p* > 0.05; **ΔVPA**: *r* = 0.052, *p* > 0.05 Δ LS BMC: **ΔMPA**: *r* = 0.044, *p* > 0.05; **ΔMVPA**: *r* = 0.072, *p* > 0.05; **ΔVPA**: *r* = 0.078, *p* > 0.05 Δ LS BA: **ΔMPA**: *r* = − 0.010, *p* > 0.05; **ΔMVPA**: *r* = 0.041, *p* > 0.05; **ΔVPA**: *r* = 0.100, *p* > 0.05 Δ FN BMD: **ΔMPA**: *r* = 0.082, *p* > 0.05; **ΔMVPA**: *r* = 0.154, *p* < 0.05; **ΔVPA**: *r* = 0.205, *p* < 0.05 Δ FN BMC: **ΔMPA**: *r* = 0.053, *p* > 0.05; **ΔMVPA**: *r* = 0.115, *p* > 0.05; **ΔVPA**: *r* = 0.163, *p* < 0.05 Δ FN BA: **ΔMPA**: *r* = − 0.016, *p* > 0.05; **ΔMVPA**: *r* = 0.001, *p* > 0.05; **ΔVPA**: *r* = 0.023, *p* > 0.05
Janz et al 2014 [65] Longitudinal ◆◆◆	Age 5: *n* = 369 (172/197) Age 8: *n* = 449 (215/234) Age 11: *n* = 452 (217/235) Age 13: *n* = 410 (205/205) Age 15: *n* = 307 (158/149) **Iowa Bone Development Study** Maturity offset estimated at ages 11, 13 and 15 and dichotomised into 0 premature and 1, mature (*n* NR)	Actigraph 7164 at ages 5, 8, 11 and 13 . Actigraph GT1M at 15 **60-s epoch** (5 s from GT1M, integrated into 60 s) Location: NR Worn for: Age 5: 4 days (1 WE) during waking hours Ages 13 and 15: 5 days (including both WE days) For inclusion: ≥ 3 days with ≥ 8 h for each measurement period Non-wear: NR	MVPA, ≥ 2296 cpm VPA, ≥ 4012 cpm Age 5 (min/day): **MVPA**: m, 59.0 ± 23.7; f, 46.7 ± 19.9*; **VPA**: m, 12.9 ± 9.4; f, 9.9 ± 7.9* Age 8: **MVPA**: m, 64.2 ± 27.3; f, 45.9 ± 20.6* **VPA**: m, 17.9 ± 13.7; f, 11.8 ± 8.8* Age 11: **MVPA**: m, 64.4 ± 28.5; f, 38.6 ± 18.5* **VPA**: m, 22.2 ± 15.6; f, 10.5 ± 8.3* Age 13: **MVPA**: m, 50.5 ± 23.6; f, 32.3 ± 18.0* **VPA**: m, 16.1 ± 11.8; f, 9.3 ± 9.2* Age 15:**MVPA**: m, 37.6 ± 19.4; f, 25.9 ± 16.6* **VPA**: m, 10.4 ± 10.2; f, 6.8 ± 8.2*	DXA (ages 5 and 8: Hologic QDR 2000; ages 11, 13 and 15: Hologic QDR 4500 (Delphi upgrade)) Translational equations for 4500 DXA measures to 2000 DXA used to adjust for differences between machines Hip, LS BMC (g)	Used sex-specific multi-level models (MLM; random and fixed effects) to create BMC growth curves for individual participants and to test the group effect of PA. Time-varying predictors that changed over multiple assessments included height, weight, linear age, non-linear age, maturity and either MVPA or VPA The Akaike information criterion (AIC) determined model fit, with lower AIC indicating better fit Box-Cox transformations were used for MVPA and VPA Spine BMC: Boys: **MVPA** (fixed effect): *β* estimate = 0.16, *SE* = 0.05, *p* = 0.002, *AIC* = 5471.7; **VPA** (fixed effect): *β* estimate = 0.25, *SE* = 0.10, *p* = 0.0178, *AIC* = 5474.0 Girls: **MPA** (fixed effect): *β* estimate = 0.06, *SE* = 0.05, *p* = 0.2319, *AIC* = 5495.8; **VPA** (fixed effect): *β* estimate = 0.22, *SE* = 0.10, *p* = 0.0223, *AIC* = 5490.6 Hip BMC: Boys: **MVPA** (fixed effect): *β* estimate = 0.12, *SE* = 0.03, *p* < 0.0001, *AIC* = 4382.1; **VPA** (fixed effect): *β* estimate = 0.23, *SE* = 0.06, *p* = 0.0001, *AIC* = 4382.2 Girls: **MVPA** (fixed effect): *β* estimate = 0.06, *SE* = 0.02, *p* = 0.0072, *AIC* = 3909.3; **VPA** (fixed effect): *β* estimate = 0.17, *SE* = 0.05, *p* = 0.0004, *AIC* = 3902.3 In females, model fit (*AIC*) was significantly better for models with VPA than MVPA. Similar in boys
Cardadeiro et al. 2014b [64] Longitudinal ◆◆◆	*n* = 177 (81/96) Baseline: m, 10.7 ± 0.3 years; f, 10.7 ± 0.4 years 1-year follow-up: m, 11.8 ± 0.3 years; f, 11.8 ± 0.4 years Maturity offset: BL: m, − 2.87 ± 0.05; f: − 1.26 ± 0.05 FU: m, − 1.88 ± 0.07; f, − 0.03 ± 0.06	Actigraph GT1M **15-s epoch** Location: right hip Worn for: 4 days (2WK, 2WE), except WB activities and sleeping For inclusion: 4 days with ≥ 600 min Non-wear: ≥ 30 min consecutive 0 counts	MPA, 2296–4011 cpm MVPA, ≥ 2296 cpm VPA, ≥ 4012 cpm Baseline (min/day): **MPA**: m, 31.0 ± 10.9; f, 32.5 ± 11.5 **MVPA**: m, 44.3 ± 17.5; f, 46.1 ± 18.3 **VPA**: m, 13.3 ± 7.5; f, 13.7 ± 8.5 Follow-up: **MPA**: m, 39.7 ± 11.2; f, 28.5 ± 11.3* **MVPA**: m, 58.6 ± 19.2; f, 40.1 ± 17.2* **VPA**: m, 18.9 ± 9.7; f, 11.6 ± 7.4*	DXA (QDR Explorer, Hologic) PF and subregions (neck, TR, SL neck, IM neck) BMD (g/cm^2^)	Used a longitudinal data approach to control for unobservable individual effects. Linear regression models were used to examine the effects of explanatory variables (PA) on PF subregional BMDs (response variables). Adjusted for maturity, body height and body lean mass FN BMD: Boys: **MPA**: NS; **MVPA**: NS; **VPA**: NS; girls: **MPA**: NS; **MVPA**: NS; **VPA**: NS Superlateral FN BMD: Boys: **MPA**: NS; **MVPA**: NS; **VPA**: NS; girls: **MPA**: NS; **MVPA**: NS; **VPA**: NS Inferomedial FN BMD: Boys: **MPA**: NS; **MVPA**: NS; **VPA**: NS; girls: **MPA**: NS; **MVPA**: NS; **VPA**: NS Trochanter BMD: Boys: **MPA**: coefficient estimate = 0.0005, robust *SE* = 0.0002, *p* < 0.05; **MVPA**: NS; **VPA**: NS Girls: **MPA**: NS; **MVPA**: NS; **VPA**: NS
Tamme et al. 2019 [66] Longitudinal ◆◆◆	Boys only *n* = 88 T1 (BL): 12.1 years T2 (1 yr FU): 13.1 years T3 (2 yr FU): 14.0 years T4 (6 yr FU): 18.0 years Tanner stages 1/2/3/4/5 (*n*): T1: 1/33/45/9/0 T2: 0/8/43/30/7 T3: 0/2/18/33/35 Skeletal age (years; mean with ± 1 SD): T1: 11.9 (10.73–13.0) T2: 13.0 (11.8–14.2); T3: 13.9 (12.9–15.0)	Actrigraph GT1M at T1, T2 and T3 Actigraph GT3X at T4 **Epoch NR** Location: right hip Worn for: 7 days (during waking) For inclusion: ≥ 10 h per day for ≥ 2 WK and 1 WE day Non-wear: all night activity (24:00–6:00 h) and ≥ 10 min consecutive zero counts	MPA, 2000–3999 cpm MVPA, ≥ 2000 cpm VPA, ≥ 4000 cpm (min/day): Median (25th; 75th percentile) **MPA**: T1, 45.8 (25.7; 59.4) T2, 42.8 (32.4; 51.2) T3, 34.1 (27.1; 45.8) T4, 27.0 (17.8; 33.4) **MVPA**: T1, 64.1 (46.3; 84.4) T2, 59.9 (47.0; 75.4) T3, 52.0 (38.1; 67.7) T4, 50.0 (35.3; 69.7) **VPA**: T1, 15.1 (9.1; 24.2) T2, 16.5 (11.3; 23.8) T3, 13.7 (9.6; 27.8) T4, 25.3 (14.8; 35.1)	DXA (Hologic QDR series) TBLH, LS, FN BMD (g/cm^2^), BMC (g), BA for calculation of BMAD (g/cm^3^), TB BMC/height	Stepwise multiple regression analysis with bone mineral characteristics entered as dependent variables and mean (T1 + T2 + T3/3) body mass, baseline bone age and mean (T1 + T2 + T3/3) PA variables (sedentary, LPA, MPA, VPA, MVPA and total PA) entered as independent variables. Only significant variables included in regression TB BMD: **MPA**: NS; **MVPA**: NS; **VPA**: NS TBLH BMC: **MPA**: NS; **MVPA**: NS; **VPA**: NS TB BMAD: **MPA**: NS; **MVPA**: NS; **VPA**: NS TB BMC/height: **MPA**: NS; **MVPA**: NS; **VPA**: NS LS BMD: **MPA**: NS; **MVPA**: NS; **VPA**: NS LS BMC: **MPA**: NS; **MVPA**: NS; **VPA**: NS LS BMAD: **MPA**: NS; **MVPA**: NS; **VPA**: NS FN BMD: **MPA**: NS; **MVPA**: NS; **VPA**: R^2^ × 100 (% of variability explained by body mass and VPA) = 43.2, VPA estimate = 0.003, *SE* = 0.001, *p* = 0.003 FN BMC: **MPA**: NS; **MVPA**: NS; **VPA**: *R*^2^ × 100 (% of variability explained by body mass, bone age and VPA) = 47.2, VPA estimate = 0.013, *SE* = 0.007, *p* = 0.045
Yamakita et al. 2019 [62] Cross-sectional ◆	*n* = 134 (60/74) m, 10.8 ± 0.4 years f, 10.8 ± 0.4 years Pubertal status classified as dichotomous variables (pre- or post-menarche) because of the low % of post-menarche girls *n* (%) = 9 (12.2)	Lifecorder GS **2-min epoch** Location: right waist Worn for: 2 weeks (during waking, except WB activities or high-risk sport) For inclusion: ≥ 10 h per day for 4 days (3 WK and 1 WE day) Non-wear: ≥ 20 min consecutive zero counts	Categorised by intensity into 11 levels (0, 0.5 and 1.0–9.0) Level 0 = immobility; levels 0.5–9.0 reflect movement by intensity level MPA: levels 4–6 (≥ 3– < 6METs) MVPA: levels 4–9 (≥ 3 METs) VPA: levels 7–9 (≥ 6 METs) (min/day) **MPA**: m, 40.9 ± 11.3; f, 38.1 ± 11.0 **MVPA**: m, 68.3 ± 20.4; f, 58.9 ± 16.4* **VPA**: m, 27.4 ± 12.8; f, 20.8 ± 8.5*	QUS (Achilles A-1000 Insight, GE Healthcare) Right calcaneus Bone stiffness (SI)	Pearson’s correlation coefficient was used to examine the relationship between PA variables (MPA, MVPA, VPA) and SI Calcaneal SI: Boys: **MPA**: *r* = 0.281, *p* = 0.030; **MVPA**: *r* = 0.361, *p* = 0.005; **VPA**: *r* = 0.326, *p* = 0.011 Girls: **MPA**: *r* = 0.155, *p* = 0.188; **MVPA**: *r* = 0.162; *p* = 0.167; **VPA**: *r* = 0.112, *p* = 0.342
Herrmann et al. 2015 [49] Cross-sectional ◆◆	*n* = 4465 Preschool *n* = 1512 (804/708) Primary school *n* = 2953 (1409/1544) pre, 4.4 ± 0.9 years primary, 8.1 ± 1.2 years **IDEFICS study** NR	Actigraph Actitrainer or GT1M **60-s epoch** Location: right hip Worn for: NR For inclusion: ≥ 3 days with ≥ 6 h per day (including 1 WE day) Non-wear: ≥ 20 min consecutive 0 counts	MPA, 2296–4011 cpm MVPA, ≥ 2296 cpm VPA, ≥ 4012 cpm Preschool (min/day): **MPA**: m, 38 ± 18; f, 29 ± 14 **MVPA**: m, 45 ± 23; f, 35 ± 18 **VPA**: m, 7 ± 7; f, 6 ± 5 Primary school: **MPA**: m, 39 ± 19; f, 29 ± 14 **MVPA**: m, 48 ± 25; f, 36 ± 18 **VPA**: m, 9 ± 8; f, 7 ± 6	QUS (Achilles Lunar Insight) Calcaneus Bone stiffness (SI)	Multivariate linear regression was used to assess the association between PA and SI. Analyses were conducted for each PA variable and stratified for preschool and school children Adjusted for age, sex, country, FFM, milk and dairy products, daylight duration, accelerometer-based SED time and valid wear time. Additionally adjusted for jumping distance and handgrip strength in school children Preschool children calcaneal SI: **MPA** (per 10 min/day): *β* = 0.75, *R*^2^ = 19.1%, *p* = 0.003; **MVPA** (per 10 min/day): *β* = 0.58, *R*^2^ = 19.0%, *p* = 0.003; **VPA** (per 10 min/day): *β* = 1.22, *R*^2^ = 18.8%, *p* = 0.05 School children calcaneal SI: **MPA** (per 10 min/day) *β* = 0.78, *R*^2^ = 27.1%, *p* < 0.001; **MVPA** (per 10 min/day): *β* = 0.61, *R*^2^ = 27.2%, *p* < 0.001; **VPA** (per 10 min/day): *β* = 1.43, *R*^2^ = 26.9%, *p* < 0.001
De Smet et al. 2015 [44] Cross-sectional ◆◆	*n* = 234 (119/115) 9.8 ± 1.5 years **ChiBS study** Pre and early pubertal m: Tanner stage 0 = 71.5% Tanner stage 1 = 28.5% f: Tanner stage 0 = 66.0% Tanner stage 1 = 34.0%	Actigraph GT3X, GT1M and Actitrainer **15-s epoch** Location: right hip Worn for: 5 days (WK and WE) during waking hours For inclusion: ≥ 3 days with min 8 h and max 18 h wear Non-wear: ≥ 20 min consecutive 0 counts	MPA, 2296–4011 cpm MVPA, ≥ 2296 cpm VPA, ≥ 4012 cpm (% recorded time) **MPA**: ALL, 4.92 ± 1.82 m, 5.47. ± 1.96, f, 4.35 ± 1.47* **MVPA**: ALL, 7.46 ± 3.24 m, 8.21 ± 3.38, f, 6.69. ± 2.90* **VPA**: ALL, 2.55 ± 1.76 m, 2.74 ± 1.76, f, 2.34 ± 1.75*	QUS (Lunar Achilles Insight, GE healthcare) Heel BUA (dB/MHz), SoS (m/s), SI (%)	Linear regression was used to examine the associations of PA with SoS, BUA and SI. Adjusted for gender, age and fat mass (SoS model also adjusted for International Standard Classification of Education) SoS: **MPA**: *B* = 1.11, Std *β* = 0.09, *R*^2^ = 0.14, *p* = 0.20; **MVPA**: *B* = 0.58, Std *β* = 0.09, *R*^2^ = 0.14, *p* = 0.21; **VPA**: *B* = 0.81, Std *β* = 0.06, *R*^2^ = 0.13, *p* = 0.33 BUA: **MPA**: *B* = 0.72, Std *β* = 0.11, *R*^2^ = 0.30, *p* = 0.08; **MVPA**: *B* = 0.54, Std *β* = 0.15, *R*^2^ = 0.31, *p* = 0.01; **VPA**: *B* = 1.02, Std *β* = 0.15, *R*^2^ = 0.32, *p* = 0.01 SI: **MPA**: *B* = 0.79, Std *β* = 0.12, *R*^2^ = 0.22, *p* = 0.06; **MVPA**: *B* = 0.52, Std *β* = 0.15, *R*^2^ = 0.22, *p* = 0.02; **VPA**: *B* = 0.89, Std *β* = 0.14, *R*^2^ = 0.22, *p* = 0.03
Yao et al. 2011 [63] Cross-sectional ◆	*n* = 75 (girls only) Girls: NW *n* = 21 OW *n* = 19 Adolescents NW: *n* = 13; OW, *n* = 22 Girls: NW, 10.1 ± 1.1 years OW, 10.3 ± 1.1 years Adolescents: NW, 15.3 ± 0.8 years OW, 15.5 ± 0.8 years Tanner stage 1/2/3/4/5 (*n*): NW G: 16/3/2/0/0 OW G: 11/7/1/0/0 NW A: 0/0/0/9/4 OW A: 0/0/0/15/7	Actigraph GT1M **10-s epoch** Location: right hip Worn for: 7 days (except during WB activity) For inclusion: ≥ 4 days (1 WE) with ≥ 10 h Non-wear: diary of device removal	MPA, MVPA, VPA and VVPA calculated according to Trost et al. (2001) age-appropriate thresholds Girls (min/day): **MPA**: NW, 101.5 ± 25.8 OW, 58.8 ± 30.7 **MVPA**: NW, 125.5 ± 33.2 OW, 65.9 ± 37.9 **VPA**: NW, 17.8 ± 7.2 OW, 11.8 ± 7.5 **VVPA**: NW, 4.6 ± 3.9 OW, 2.0 ± 2.3 Adolescents: **MPA**: NW, 87.9 ± 21.3 OW, 47.5 ± 19.1 **MVPA**: NW, 103.0 ± 27.2 OW, 48.5 ± 20.4 **VPA**: NW, 3.4 ± 3.6 OW, 1.5 ± 1.0 **VVPA**: NW, 0.6 ± 0.9 OW, 0.4 ± 0.8	QUS (Sunlight Omnisense model 7000P, Sunlight Medical Ltd.) 1/3 radius, midshaft tibia SOS (m/s)	Pearson product moment correlations were used to determine correlations between SoS and MPA, MVPA, VPA and VVPA (age was partialed out) Dominant tibia SoS: **MPA**: *r* = 0.36, *p* < 0.05; **MVPA**: *r* = 0.38, *p* < 0.05; **VPA**: *r* = 0.37, *p* < 0.05; **VVPA**: NS Non-dominant tibia SoS: **MPA**: *r* = 0.37, *p* < 0.05; **MVPA**: *r* = 0.4, *p* < 0.01; **VPA**: *r* = 0.42, *p* < 0.01; **VVPA**: NS Dominant distal 1/3 radius: **MPA**: NS; **MVPA**: NS; **VPA**: NS; **VVPA**: NS Non-dominant distal 1/3 radius: **MPA**: NS; **MVPA**: NS; **VPA**: NS; **VVPA**: NS
Szmodis et al. 2019 [58] Cross-sectional ◆	*n* = 123 (64/59) m, 11.12 ± 0.68 years f, 10.96 ± 0.75 years All girls pre-menarcheal	Actigraph GT3X + **5-s epoch** Location: NR Worn for: 5 days (1 WE) 24 h/day, except WB activities For inclusion: 5 days with measured daily activity from 6 am to 8 pm (1WE) Non-wear: NR	MPA, 500–3999 cpm MVPA, > 500 cpm VPA, 4000–7599 cpm VVPA, > 7600 cpm (min/day): **MPA**: m, 138.07 ± 30.63 f, 117.7 ± 38.79* ALL, 128.36 ± 37.48 **MVPA**: m, 159.23 ± 36.90; f, 133.44 ± 44.28* ALL, 147.37 ± 44.13 **VPA**: m, 22.13 ± 12.13; f, 16.27 ± 8.24* ALL, 19.43 ± 10.19 **VVPA**: m, 3.13 ± 2.22 f, 4.21 ± 4.07 ALL, 3.63 ± 3.27	QUS (SONOST3000, Seoul, Korea) Calcanues SOS (m/s), BUA (dBMHz), BQI	Pearson’s correlation analysed the relationship between bone and PA parameters (MPA, MVPA, VPA, VVPA) SOS: Whole sample: **MPA**: *r* = 0.05, *p* > 0.05; **MVPA**: *r* = 0.09, *p* > 0.05; **VPA**: *r* = 0.23, *p* < 0.05; **VVPA**: − 0.09, *p* > 0.05 Boys: **MPA**: *r* = 0.09, *p* > 0.05; **MVPA**: *r* = 0.23, *p* > 0.05; **VPA**: *r* = 0.46, *p* < 0.001; **VVPA**: 0.21, *p* > 0.05 Girls: **MPA**: *r* = − 0.10, *p* > 0.05; **MVPA**: *r* = − 0.12, *p* > 0.05; **VPA**: *r* = − 0.10, *p* > 0.05; **VVPA**: − 0.17, *p* > 0.05 BUA: Whole sample: **MPA**: *r* = 0.08, *p* > 0.05; **MVPA**: *r* = 0.12, *p* > 0.05; **VPA**: *r* = 0.25, *p* < 0.05; **VVPA**: − 0.03, *p* > 0.05 Boys: **MPA**: *r* = 0.15, *p* > 0.05; **MVPA**: *r* = 0.15, *p* > 0.05; **VPA**: *r* = 0.33, *p* < 0.01; **VVPA**: 0.14, *p* > 0.05 Girls: **MPA**: *r* = − 0.02, *p* > 0.05; **MVPA**: *r* = − 0.01, *p* > 0.05; **VPA**: *r* = 0.00, *p* > 0.05; **VVPA**: − 0.10, *p* > 0.05 BQI: Whole sample: **MPA**: *r* = 0.12, *p* > 0.05; **MVPA**: *r* = 0.15, *p* > 0.05; **VPA**: *r* = 0.29, *p* < 0.01; **VVPA**: − 0.06, *p* > 0.05 Boys: **MPA**: *r* = 0.12, *p* > 0.05; **MVPA**: *r* = 0.25, *p* > 0.05; **VPA**: *r* = 0.47, *p* < 0.001; **VVPA**: 0.23, *p* > 0.05 Girls: **MPA**: *r* = − 0.06, *p* > 0.05; **MVPA**: *r* = − 0.07, *p* > 0.05; **VPA**: *r* = − 0.08, *p* > 0.05; **VVPA**: − 0.16, *p* > 0.05
Sayers et al. 2011 [24] Cross-sectional ◆◆	*n* = 1748 (778/970) 15.4 ± 0.23 years **ALSPAC** Tanner stages 1–2/3/4–5 (%): m, 2/12/86 f, 1/8/91	MTI 7164 or Actigraph GT1M **60-s epoch** Location: right hip Worn for: 7 days during waking hours (except WB activity) For inclusion: ≥ 3 days with ≥ 10 h Non-wear: NR	MPA, 3600–6199 cpm VPA, > 6200 cpm (min/day) **MPA**: m, 25.6 ± 17.1; f, 15.8 ± 13.2 **VPA**: m, 4.3 ± 5.8 f, 2.2 ± 3.7	pQCT (Stratec XCT2000L) Mid (50%) right tibia BMCc (mg), BMDc (mg/cm^3^), BAc (mm^2^), PC (mm), EC (mm)	Linear regression was used to examine associations between MPA and VPA and pQCT bone outcomes (adjusted for age and height at time of pQCT measurement and duration of accelerometer recording) *β* coefficient represents SD change in pQCT parameter per doubling in MPA or VPA Cortical BMC: Boys: **MPA**: *β* (*95% CI*) = 0.011 (− 0.033, 0.054), *p* = 0.6303; **VPA**: *β* (*95% CI*) = 0.041 (0.004, 0.077), *p* = 0.0280 Girls: **MPA**: *β* (*95% CI*) = 0.033 (− 0.002, 0.068), *p* = 0.0656; **VPA**: *β* (*95% CI*) = 0.070 (0.031, 0.109), *p* = 0.0004 All: **MPA**: *β* (*95% CI*) = 0.024 (− 0.003, 0.051), *p* = 0.0835; **VPA**: *β* (*95% CI*) = 0.055 (0.028, 0.081), *p* = 0.00001 Cortical BA: Boys: **MPA**: *β* (*95% CI*) = 0.022 (− 0.019, 0.062), *p* = 0.2959; **VPA**: *β* (*95% CI*) = 0.050 (0.016, 0.085), *p* = 0.0039 Girls: **MPA**: *β* (*95% CI*) = 0.032 (− 0.001, 0.065), *p* = 0.0611; **VPA**: *β* (*95% CI*) = 0.068 (0.032, 0.105), *p* = 0.0002 All: **MPA**: *β* (*95% CI*) = 0.028 (0.002, 0.053), *p* = 0.0347; **VPA**: *β* (*95% CI*) = 0.059 (0.034, 0.084), *p* = 0.0000 Cortical BMD: Boys: **MPA**: *β* (*95% CI*) = − 0.055 (− 0.095, − 0.015), *p* = 0.0074; **VPA**: *β* (*95% CI*) = − 0.048 (− 0.082, − 0.014), *p* = 0.0062 Girls: **MPA**: *β* (*95% CI*) = − 0.004 (− 0.037, 0.028), *p* = 0.7893; **VPA**: *β* (*95% CI*) = − 0.012 (− 0.048, 0.024), *p* = 0.5206 All: **MPA**: *β* (*95% CI*) = − 0.025 (− 0.050, 0.001), *p* = 0.0574; **VPA**: *β* (*95% CI*) = − 0.031 (− 0.056, − 0.006), *p* = 0.0150 Periosteal circumference: Boys: **MPA**: *β* (*95% CI*) = − 0.009 (− 0.050, 0.031), *p* = 0.6486; **VPA**: *β* (*95% CI*) = 0.020 (− 0.015, 0.054), *p* = 0.2671 Girls: **MPA**: *β* (*95% CI*) = 0.032 (− 0.001, 0.065), *p* = 0.0606; **VPA**: *β* (*95% CI*) = 0.057 (0.021, 0.094), *p* = 0.0022 All: **MPA**: *β* (*95% CI*) = 0.015 (− 0.010, 0.041), *p* = 0.2439; **VPA:** *β* (*95% CI*) = 0.037 (0.012, 0.063), *p* = 0.0037 Endosteal circumference (adjusted periosteal circumference): Boys: **MPA**: *β* (*95% CI*) = − 0.029 (− 0.064, 0.005), *p* = 0.0946; **VPA**: *β* (*95% CI*) = − 0.044 (− 0.073, − 0.015), *p* = 0.0033 Girls: **MPA**: *β* (*95% CI*) = − 0.012 (− 0.041, 0.016), *p* = 0.3901; **VPA**: *β* (*95% CI*) = − 0.038 (− 0.069, − 0.007), *p* = 0.0167 All: **MPA**: *β* (*95% CI*) = − 0.019 (− 0.041, 0.003), *p* = 0.0848; **VPA**: *β* (*95% CI*) = − 0.041 (− 0.062, − 0.020), *p* = 0.00002
Specker et al., 2001^†^ [57] Cross-sectional ◆	*n* = 239 (124/115) m, 4.0 ± 0.6 years f, 3.9 ± 0.6 years NR	Actiwatch motion sensor **Epoch NR** (PA described in cpm in methods, **60 s** assumed) Location: lumbar spine Worn for: 2 days For inclusion: 2 days Non-wear: NR	MVPA: > 500 cpm VPA: > 1000 cpm Average % time: **MVPA**: m, 13.5 ± 3.9; f, 12.1 ± 4.2* **VPA**: m, 5.2 ± 2.2; f, 4.5 ± 2.2*	pQCT (Norland/Stratec XCT2000) 20% distal tibia BAc (mm^2^), PC (mm), EC (mm)	Forward–backward stepwise regression was performed including variables that were significant (*p* ≤ 0.05). After obtaining final models, PA measures were added to assess whether they explained a significant amount of remaining error Periosteal circumference: **MVPA**: NS; **VPA**: NS Endosteal circumference: **MVPA**: NS; **VPA**: NS Cortical BA: **MVPA**: NS; **VPA**: NS
Meiring et al. 2014^†^ [39] Cross-sectional ◆◆	*n* = 38 (12/26) 9.9 ± 1.3 years Tanner stages 1/2 (*n*): 22/16	Actical accelerometer **15-s epoch** Location: right hip Worn for: 7 days (except WB activity) For inclusion: ≥ 4 days with ≥ 10 h consecutive activity counts Non-wear: only full days of non-wear removed (full days consecutive 0 counts or self-reported removal)	MPA, 1500–6500 cpm MVPA, > 1500 cpm VPA, > 6501 cpm (min/day): **MPA**, 55.6 ± 23.5 **MVPA**, NR **VPA**, 2.1 ± 3.0	pQCT (Stratec XCT 2000) 4% and 65% radius 65% tibia ToA (mm^2^), ToD (mg/cm^3^), TrabD (mg/cm^3^), BSI (mg^2^/cm^4^) BAc (mm^2^) BMDc (mg/cm^3^) SSI (mm^3^), PC (mm), CT (mm), EC (mm)	Pearson’s correlation was performed between MPA, MVPA and VPA and pQCT outcomes. pQCT outcomes adjusted for limb length, body mass and sex Radius 4% site: ToA: **MPA**: *r* = 0.23, *p* > 0.05; **MVPA**: *r* = 0.19, *p* > 0.05; **VPA**: *r* = 0.26, *p* > 0.05; ToD: **MPA**: *r* = 0.41, *p* < 0.05; **MVPA**: *r* = 0.40, *p* < 0.05; **VPA**: *r* = 0.15, *p* > 0.05; TrabD: **MPA**: *r* = 0.24, *p* > 0.05; **MVPA**: *r* = 0.24, *p* > 0.05; **VPA**: *r* = 0.18, *p* > 0.05; BSI: **MPA**: *r* = 0.39, *p* < 0.05; **MVPA**: *r* = 0.33, *p* > 0.05; **VPA**: *r* = 0.33, *p* > 0.05 Radius 65% site: ToA: **MPA**: *r* = 0.17, *p* > 0.05; **MVPA**: *r* = 0.16, *p* > 0.05; **VPA**: *r* = 0.20, *p* > 0.05; BAc: **MPA**: *r* = 0.03, *p* > 0.05; **MVPA**: *r* = 0.06, *p* > 0.05; **VPA**: *r* = 0.37, *p* < 0.05; BMDc: **MPA**: *r* = 0.06, *p* > 0.05; **MVPA**: *r* = 0.06, *p* > 0.05; **VPA**: *r* = 0.28, *p* > 0.05; SSI: **MPA**: *r* = − 0.03, *p* > 0.05; **MVPA**: *r* = − 0.03, *p* > 0.05; **VPA**: *r* = 0.15, *p* > 0.05; PC: **MPA**: *r* = 0.34, *p* > 0.05; **MVPA**: *r* = 0.35, *p* > 0.05; **VPA**: *r* = 0.22, *p* > 0.05; CT: **MPA**: *r* = 0.10, *p* > 0.05; **MVPA**: *r* = 0.10, *p* > 0.05; **VPA**: *r* = 0.29, *p* > 0.05; EC: **MPA**: *r* = 0.27, *p* > 0.05; **MVPA**: *r* = 0.27, *p* > 0.05; **VPA**: *r* = 0.06, *p* > 0.05 Tibia 65% site: ToA: **MPA**: *r* = 0.25, *p* > 0.05; **MVPA**: *r* = 0.24, *p* > 0.05; **VPA**: *r* = 0.30, *p* > 0.05; BAc: **MPA**: *r* = 0.15, *p* > 0.05; **MVPA**: *r* = 0.14, *p* > 0.05; **VPA**: *r* = 0.30, *p* > 0.05; BMDc: **MPA**: *r* = 0.01, *p* > 0.05; **MVPA**: *r* = 0.03, *p* > 0.05; **VPA**: *r* = 0.16, *p* > 0.05; SSI: **MPA**: *r* = 0.21, *p* > 0.05; **MVPA**: *r* = 0.19, *p* > 0.05; **VPA**: *r* = 0.32, *p* > 0.05; PC: **MPA**: *r* = 0.23, *p* > 0.05; **MVPA**: *r* = 0.21, *p* > 0.05; **VPA**: *r* = 0.28, *p* > 0.05; CT: **MPA**: *r* = 0.02, *p* > 0.05; **MVPA**: *r* = 0.003, *p* > 0.05; **VPA**: *r* = 0.19, *p* > 0.05; EC: **MPA**: *r* = 0.25, *p* > 0.05; **MVPA**: *r* = 0.24, *p* > 0.05; **VPA**: *r* = 0.22, *p* > 0.05
Tan et al. 2018 [59] Cross-sectional ◆◆	*n* = 101 (39/62) 15.4 ± 0.04 years **Health Promoting Secondary Schools study** Years post-APHV, 2.58 ± 0.76 years Girls’ age at menarche, 12.3 ± 1.2 years	Actigraph GT1M **15-s epoch** Location: right iliac crest Worn for: 7 days (except WB activities) For inclusion: ≥ 3 days with ≥ 10 h Non-wear: log sheet recording wear 60 min consecutive 0 counts	MVPA, ≥ 2296 cpm VPA, ≥ 4012 cpm (min/day): **MVPA**, 49.6 ± 21.8 **VPA**, 19.5 ± 13.0	pQCT (Stratec XCT3000) **8% tibia**: BSI (mg^2^/mm^4^), ToA (mm^2^), ToD (mg/cm^3^) **50% tibia**: polar SSI (mm^3^), ToA (mm^2^), BAc (mm^2^), medullary area (mm^2^), BMDc (mg/cm^3^)	Used multiple variable regression models to assess the relations between PA and bone outcomes (fully adjusted model controlled for sex, ethnicity, maturity offset, tibial length, lean mass and sedentary time) 8% site: Bone strength index: **MVPA**: *B* (*95% CI*) = 13.9 (− 3.8, 31.7), *β* = 0.15, *R*^2^ of total model = 0.51, *p* > 0.05; **VPA**: *B* (*95% CI*) = 34.0 (6.9, 61.1), *β* = 0.22, *R*^2^ of total model = 0.52, *p* < 0.05—explained 7% variance, *p* = 0.01 Total bone CSA: **MVPA**: *B* (*95% CI*) = 0.61 (− 0.55, 1.78), *β* = 0.10, *R*^2^ of total model = 0.55 *p* > 0.05; **VPA**: *B* (*95% CI*) = 0.55 (− 1.28, 2.37), *β* = 0.05, *R*^2^ of total model = 0.55, *p* > 0.05 Total bone density: **MVPA**: *B* (*95% CI*) = 0.09 (− 0.42, 0.60), *β* = 0.04, *R*^2^ of total model = 0.29 *p* > 0.05; **VPA**: *B* (*95% CI*) = 0.45 (− 0.34, 1.24), *β* = 0.12, *R*^2^ of total model = 0.30, *p* > 0.05 50% site: Polar SSI: **MVPA:** *B* (*95% CI*) = 2.8 (0.3, 5.4), *β* = 0.15, *R*^2^ of total model = 0.76, *p* < 0.05—explained 3% of the variance, *p* = 0.01; **VPA:** *B* (*95% CI*) = 3.6 (− 0.4, 7.6), *β* = 0.11, *R*^2^ of total model = 0.75, *p* > 0.05 Total bone CSA: **MVPA:** *B* (*95% CI*) = 0.39 (− 0.05, 0.82), *β* = 0.79, *R*^2^ of total model = 0.11, *p* > 0.05; **VPA**: *B* (*95% CI*) = 0.42 (− 0.26, 1.11), *β* = 0.07, *R*^2^ of total model = 0.78, *p* > 0.05 Cortical area: **MVPA:** *B* (*95% CI*) = 0.24 (− 0.04, 0.52), *β* = 0.10, *R*^2^ of total model = 0.79, *p* > 0.05; **VPA:** *B* (*95% CI*) = 0.36 (− 0.08, 0.80), *β* = 0.09, *R*^2^ of total model = 0.79, *p* > 0.05 Medullary area: **MVPA**: *B* (*95% CI*) = 0.15 (− 0.17, 0.46), *β* = 0.09, *R*^2^ of total model = 0.47, *p* > 0.05; **VPA**: *B* (*95% CI*) = 0.06 (− 0.43, 0.56), *β* = 0.02, *R*^2^ of total model = 0.46, *p* > 0.05 Cortical density: **MVPA**: *B* (*95% CI*) = 0.01 (− 0.22, 0.24), *β* = 0.005, *R*^2^ of total model = 0.76, *p* > 0.05 **VPA**: *B* (*95% CI*) = 0.09 (− 0.26, 0.45), *β* = 0.03, *R*^2^ of total model = 0.76, *p* > 0.05
Kehrig et al. 2018 [51] Cross-sectional ◆◆	*n* = 49 (26/23) m, 11.4 ± 1.7 years f, 10.7 ± 1.6 years ALL, 11.0 ± 1.7 years Estimated years from age at PHV: m, − 1.8 ± 1.4 years f, − 1.1 ± 1.4 years ALL, − 1.4 ± 1.4 years	Actigraph wGT3X-BT **10-s epoch** Location: right hip (midaxillary line) Worn for: 7 days For inclusion: ≥ 8 h per day for ≥ 2 days (min of 1 WE day) Non-wear: ≥ 20 min consecutive 0 counts	MVPA, ≥ 2296 cpm VPA, ≥ 4012 cpm (min/day) **MVPA**: m, 54.7 ± 25.8; f, 45.0 ± 17.4; ALL, 50.2 ± 22.6 **VPA**: m, 20.3 ± 12.2; f, 16.8 ± 8.2; ALL, 18.6 ± 10.6	pQcT (XCT2000, Stratec) Distal sites: 4% radius, 4% tibia Shaft sites: 65% radius, 66% tibia BSIc (mg^2^/mm^4^) at distal sites SSIp (mm^3^) at shaft sites	A two-step hierarchical linear regression model was used. The first step was a base model containing sex, body mass, and site-specific muscle cross-sectional area. The second included MVPA, VPA and daily impact counts in each base model to assess whether they independently predicted variance in bone strength at the distal and shaft sites of the radius and tibia. Present Δ*R*^2^ after inclusion of PA outcomes and standardised *β* coefficient Distal radius BSIc: **MVPA**: Δ*R*^2^ = 0.000, Std. *β* = 0.002, *p* = 0.990; **VPA**: Δ*R*^2^ = 0.001, Std. *β* = 0.027, *p* = 0.883; Radius shaft SSIp: **MVPA**: Δ*R*^2^ = 0.005, Std. *β* = 0.070, *p* = 0.395; **VPA**: Δ*R*^2^ = 0.009, Std. *β* = 0.097, *p* = 0.232; Distal tibia BSIc: **MVPA**: Δ*R*^2^ = 0.043, Std. *β* = 0.213, *p* = 0.056; **VPA**: Δ*R*^2^ = 0.055, Std. *β* = 0.239, *p* = 0.029; Tibia shaft SSIp: **MVPA**: Δ*R*^2^ = 0.037, Std. *β* = 0.200, *p* = 0.020; **VPA**: Δ*R*^2^ = 0.053, Std. *β* = 0.236, *p* = 0.005
Deere et al. 2012b [46] Cross-sectional ◆◆	*n* = 675 (272/403) 17.7 ± 0.3 years **ALSPAC** NR	Newtest monitor **Raw data** Location: NR Worn for: 7 days (during waking hours, except WB activity) For inclusion: ≥ 2 days with ≥ 8 h recording Non-wear: kept diary of monitor wear	3 impact bands: 0.5–2.1 g (low) 2.1–4.2 g (medium) > 4.2 g (high impacts) Median counts per day by quartile of *g* band: Low impact: Q1: m, 1687; f, 1649; all, 1656 Q2: m, 3359; f, 3199; all, 3278 Q3: m, 5603; f, 4764; all, 4954 Q4: m, 9388; f, 8115; all, 9037 Medium impact: Q1: m, 48; f, 33; all, 37 Q2: m, 105; f, 82; all, 91 Q3: m, 198; f, 154; all, 176 Q4: m, 499; f, 366; all, 417 High impact: Q1: m, 8; f, 5; all, 6 Q2: m, 18; f, 12; all, 14 Q3: m, 35; f,: 25; all, 28 Q4: m, 95; f, 69; all, 81	pQCT (Stratec XCT2000L; XCT custom software version 6.00B) Mid-tibia BMCc (mg), BMDc (mg/cm^3^), BAc (mm^2^), cortical thickness (mm), periosteal circumference (mm), buckling ratio, CSMI (cm^4^), SSI	Used regression analysis to examine relationships between the number of counts per person per day within the different impact bands and bone outcomes. PA data was log transformed and *β* coefficients were standardised, so *β* represents an SD change in pQCT outcome per doubling in number of impacts. Fully adjusted model included age, gender, height, fat mass and lean mass Cortical BMC and BMD: no relationships observed. After adjustment for fat and lean mass, 95% confidence limits overlapped zero Cortical BA: Boys: **low impact:** *R*^2^ = 0.29, *β* = − 0.071, *95% CI* = − 0.154, 0.013, *p* = 0.098; **medium impact:** *R*^2^ = 0.28, *β* = 0.035, *95% CI* = − 0.026, 0.096, *p* = 0.260; **high impact**: *R*^2^ = 0.29, *β* = 0.050, *95% CI* = − 0.005, 0.106, *p* = 0.076 Girls: **low impact:** *R*^2^ = 0.31, *β* = 0.008, *95% CI* = − 0.054, 0.071, *p* = 0.793; **medium impact:** *R*^2^ = 0.31, *β* = − 0.004, *95% CI* = − 0.045, 0.038, *p* = 0.860; **high impact**: *R*^2^ = 0.31, *β* = 0.020, *95% CI* = − 0.015, 0.055, *p* = 0.260 All: **low impact**: *R*^2^ = 0.56, *β* = − 0.035, *95% CI* = − 0.085, 0.016, *p* = 0.180; **medium impact**: *R*^2^ = 0.56, *β* = 0.008, *95% CI* = − 0.027, 0.042, *p* = 0.663; **high impact**: *R*^2^ = 0.57, *β* = 0.028, *95% CI* = − 0.002, 0.059, *p* = 0.070 Cortical thickness: Boys: **low impact**: *R*^2^ = 0.11, *β* = − 0.097, *95% CI* = − 0.200, 0.007, *p* = 0.066; **medium impact**: *R*^2^ = 0.10, *β* = 0.022, *95% CI* = − 0.054, 0.098, *p* = 0.563; **high impact**: *R*^2^ = 0.11, *β* = 0.032, *95% CI* = − 0.037, 0.101, *p* = 0.368 Girls: **low impact**: *R*^2^ = 0.07, *β* = 0.026, *95% CI* = − 0.064, 0.115, *p* = 0.572; **medium impact**: *R*^2^ = 0.07, *β* = 0.023, *95% CI* = − 0.036, 0.082, *p* = 0.450; **high impact**: *R*^2^ = 0.07, *β* = 0.034, *95% CI* = − 0.016, 0.084, *p* = 0.181 All: **low impact**: *R*^2^ = 0.24, *β* = − 0.037, *95% CI* = − 0.103, 0.030, *p* = 0.283; **medium impact**: *R*^2^ = 0.23, *β* = 0.018, *95% CI* = − 0.028, 0.064, *p* = 0.433; **high impact**: *R*^2^ = 0.24, *β* = 0.030, *95% CI* = − 0.010, 0.070, *p* = 0.144 Periosteal circumference: Boys: **low impact**: *R*^2^ = 0.36, *β* = − 0.026, *95% CI* = − 0.097, 0.044, *p* = 0.462; **medium impact**: *R*^2^ = 0.37, *β* = 0.039, *95% CI* = − 0.013, 0.090, *p* = 0.140; **high impact**: *R*^2^ = 0.37, *β* = 0.054, *95% CI* = 0.007, 0.100, *p* = 0.024 Girls: **low impact**: *R*^2^ = 0.41, *β* = − 0.005, *95% CI* = − 0.066, 0.056, *p* = 0.873; **medium impact:** *R*^2^ = 0.41, *β* = − 0.023, *95% CI* = − 0.064, 0.017, *p* = 0.260; **high impact**: *R*^2^ = 0.41, *β* = 0.007, *95% CI* = − 0.028, 0.041, *p* = 0.707 All: **low impact**: *R*^2^ = 0.64, *β* = − 0.020, *95% CI* = − 0.066, 0.026, *p* = 0.390; **medium impact**: *R*^2^ = 0.63, *β* = 0.000, *95% CI* = − 0.032, 0.031, *p* = 0.982 (sig. gender interaction *p* = 0.033); **high impact**: *R*^2^ = 0.64, *β* = 0.022, *95% CI* = − 0.005, 0.050, *p* = 0.113 Buckling ratio: Boys: **low impact**: *R*^2^ = 0.06, *β* = 0.081, *95% CI* = − 0.027, 0.189, *p* = 0.141; **medium impact**: *R*^2^ = 0.05, *β* = 0.003, *95% CI* = − 0.076, 0.082, *p* = 0.944; **high impact**: *R*^2^ = 0.05, *β* = 0.004, *95% CI* = − 0.068, 0.076, *p* = 0.905 Girls: **low impact**: *R*^2^ = 0.06, *β* = − 0.032, *95% CI* = − 0.136, 0.072, *p* = 0.542; **medium impact**: *R*^2^ = 0.06, *β* = − 0.045, *95% CI* = − 0.114, 0.024, *p* = 0.200; **high impact**: *R*^2^ = 0.06, *β* = − 0.034, *95% CI* = − 0.093, 0.024, *p* = 0.250 All: **low impact**: *R*^2^ = 0.06, *β* = 0.022, *95% CI* = − 0.052, 0.096, *p* = 0.561; **medium impact**: *R*^2^ = 0.06, *β* = − 0.023, *95% CI* = − 0.074, 0.028, *p* = 0.380; **high impact**: *R*^2^ = 0.06, *β* = − 0.018, *95% CI* = − 0.063, 0.027, *p* = 0.433 CSMI: Boys: **low impact**: *R*^2^ = 0.37, *β* = − 0.045, *95% CI* = − 0.126, 0.037, *p* = 0.281; **medium impact**: *R*^2^ = 0.37, *β* = 0.030, *95% CI* = − 0.021, 0.098, *p* = 0.202; **high impact**: *R*^2^ = 0.38, *β* = 0.054, *95% CI* = 0.000, 0.108, *p* = 0.049 Girls: **low impact**: *R*^2^ = 0.41, *β* = 0.002, *95% CI* = − 0.050, 0.054, *p* = 0.940; **medium impact**: *R*^2^ = 0.41, *β* = − 0.015, *95% CI* = − 0.049, 0.019, *p* = 0.386; **high impact:** *R*^2^ = 0.41, *β* = 0.010, *95% CI* = − 0.019, 0.039, *p* = 0.509 All: **low impact**: *R*^2^ = 0.64, *β* = − 0.026, *95% CI* = − 0.071, 0.020, *p* = 0.268; **medium impact:** *R*^2^ = 0.64, *β* = 0.002, *95% CI* = − 0.029, 0.034, *p* = 0.893; **high impact**: *R*^2^ = 0.64, *β* = 0.023, *95% CI* = − 0.004, 0.051, *p* = 0.099 SSI: Boys: **low impact**: *R*^2^ = 0.36, *β* = − 0.054, *95% CI* = − 0.134, 0.026, *p* = 0.187; **medium impact**: *R*^2^ = 0.36, *β* = 0.039, *95% CI* = − 0.020, 0.097, *p* = 0.192; **high impact**: *R*^2^ = 0.37, *β* = 0.053, *95% CI* = 0.000, 0.106, *p* = 0.049 Girls: **low impact**: *R*^2^ = 0.38, *β* = − 0.002, *95% CI* = − 0.060, 0.056, *p* = 0.936; **medium impact**: *R*^2^ = 0.38, *β* = − 0.013, *95% CI* = − 0.052, 0.025, *p* = 0.491; **high impact**: *R*^2^ = 0.38, *β* = 0.015, *95% CI* = − 0.018, 0.047, *p* = 0.377 All: **low impact**: *R*^2^ = 0.61, *β* = − 0.033, *95% CI* = − 0.081, 0.015, *p* = 0.173; **medium impact**: *R*^2^ = 0.61, *β* = 0.003, *95% CI* = − 0.030, 0.035, *p* = 0.877 (sig gender interaction *p* = 0.047); **high impact**: *R*^2^ = 0.61, *β* = 0.025, *95% CI* = − 0.003, 0.054, *p* = 0.085

*Abbreviations*: *n*, sample size; *m*, male; *f*, female; *WB*, water-based;* NR*, not reported; *MPA*, moderate physical activity; *MVPA*, moderate-vigorous physical activity; *VPA*, vigorous physical activity; *VVPA*, very vigorous physical activity; *cpm*, counts per minute; *WK*, weekday; *WE*, weekend day; *CI*, confidence intervals; *g*, gravitational force; *MDP*, Mediterranean diet pattern; *BL*, baseline; *FU*, follow-up; *AIC*, Akaike information criterion; *NS*, non-significant; *METs*, metabolic equivalents; *FFM*, fat-free mass;* NW*, normal weight; *OW*, overweight; *PHV*, peak height velocity. *Bone*: *DXA*, dual-energy x-ray absorptiometry; *PF*, proximal femur; *FN*, femoral neck; *TR*, trochanter; *IT*, intertrocanter; *TB*, total body; *LS*, lumbar spine; *UL*, upper limbs; *LL*, lower limbs; *SL*, superlateral; *IM*, inferomedial; *TBLH*, total body less head; *BMC*, bone mineral content; *aBMD*, areal bone mineral density; *BA*, bone area; *aBMC*, area-adjusted BMC; *CSA*, cross-sectional area; *Z*, section modulus; *CSMI*, cross-sectional moment of inertia; *BMAD*, bone mineral apparent density; *QUS*, quantitative ultrasound; *SI*, stiffness index; *BUA*, Broadband Ultrasound Attenuation; *SOS*, speed of sound; *BQI*, bone quality index; *pQCT*, peripheral quantitative computed tomography; *PC*, periosteal circumference; *EC*, endosteal circumference; *BMCc*, cortical BMC; *BMDc*, cortical BMD; *BAc*, cortical BA; *ToA*, total area; *ToD*, total density; *TrabD*, trabecular density; *BSI*, bone strength index; *SSI*, strength-strain index; *CT*, cortical thickness. *Study abbreviations*: *ALSPAC*, Avon Longitudinal Study of Parents and Children; *HELENA*, Healthy Lifestyle in Europe by Nutrition in Adolescence; *ChiBS*, Children’s body composition and stress; *CoSCIS*, The Copenhagen School Child Intervention Study; *IDEFICS*, Identification and prevention of Dietary- and lifestyle-induced health EFfects In Children and infantS. *p < 0.05; ^†^appears twice in the table as it used both DXA and pQCT (results for each presented separately in the table with other studies using the same imaging techniques); ^††^longitudinal study, however only 18-year time point had accelerometer PA data, so treated as a cross-sectional analysis

Study quality: ◆ low; ◆◆ fair; ◆◆◆ good. Based on the National Institute of Health ‘Quality Assessment Tool for Observational Cohort and Cross-Sectional Studies’

## Results

**Fig. 1:**
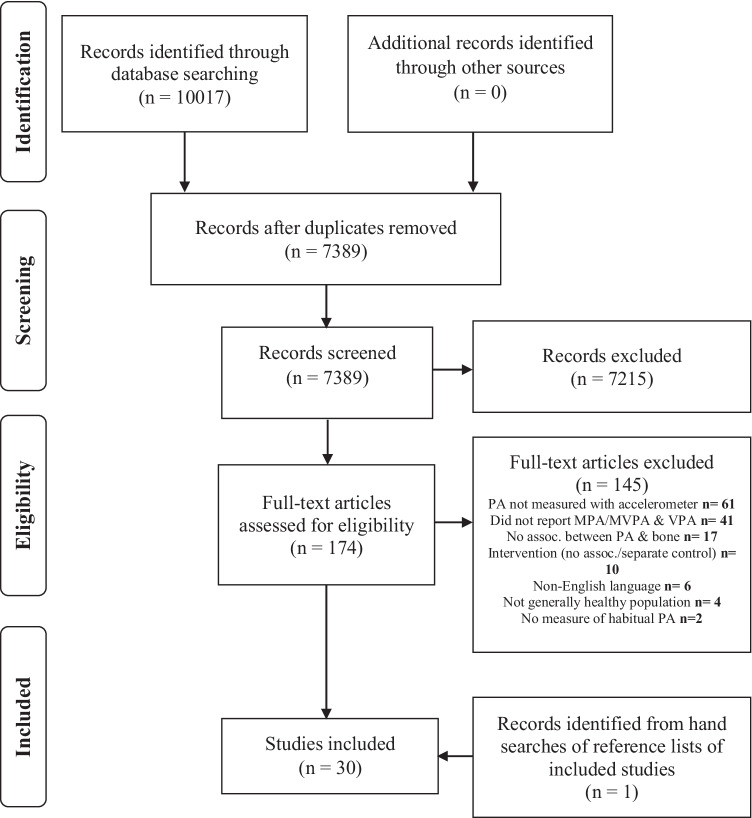
Preferred Reporting Items for Systematic reviews and Meta-Analyses (PRISMA) flow diagram of the study selection process. PA physical activity, MPA moderate physical activity, MVPA moderate-to-vigorous physical activity, VPA vigorous physical activity

The initial search strategy identified 10,017 potentially relevant articles. Following the removal of duplicates, 7389 titles and abstracts were screened and 7215 of these were not deemed to be eligible, leaving 174 articles for full-text review. Of these studies, 33 satisfied the pre-defined inclusion criteria. Four of the studies [35–38] were further excluded as multiple studies had reported on the same/similar outcomes using participants from the same cohort. The study with the most complete descriptive information on the sample, activity intensities and their respective associations with bone outcomes was kept for inclusion. An additional study [39] was obtained through the hand searching of reference lists of included studies and relevant reviews, making a total of 30 studies included in the review. A PRISMA flow diagram detailing the stages of study selection and reasons for exclusion of full texts can be seen in Fig. 1.

### Study characteristics and quality assessment

Study characteristics are presented in Table 1. Of the 30 included studies, 26 were cross-sectional [24, 39–63], three were longitudinal [64–66] and one was prospective in design [67]. Four studies included participants from the Avon Longitudinal Study of Parents and Children (ALSPAC) [24, 45, 46, 60], two from the Iowa Bone Development Study [50, 65], two from the European Youth Heart Study [42, 55] and two from the Children’s body composition and stress (CHiBS) study [44, 56]. Other studies also included participants from the Healthy Lifestyle in Europe by Nutrition in Adolescence (HELENA) study [47], the Copenhagen School Child Intervention Study (CoSCIS) [48] and the Identification and prevention of Dietary- and lifestyle-induced health EFfects In Children and infantS (IDEFICS) study [49]. The mean age of participants ranged from 4 to 18 years of age, and sample sizes ranged between 38 and 4465, with a mean sample size of 687 participants. The majority of studies (*N* = 26/30) included both boys and girls in their sample, with one study including only girls [63] and three studies including only boys [52, 66, 67]. Eleven studies assessed maturity using Tanner staging (self-reported in [24, 39, 42, 60, 63, 66, 67] and assessed by a physician in [44, 47, 55, 56]), five studies estimated it from maturity offset prediction equations [41, 51, 59, 64, 65] and three studies used skeletal age [43, 52, 66]. Two studies also assessed the presence of menarche via self-report [59, 62]. More detail regarding the characteristics of participants in included studies can be found in Table 1.

The bone imaging methods used in the included studies and anatomical sites assessed are summarised in Table 2. The majority of studies (*n* = 21/30) measured bone outcomes using dual-energy x-ray absorptiometry (DXA), with six studies using peripheral quantitative computed tomography (pQCT) and five studies using quantitative ultrasound (QUS). Two studies used both DXA and pQCT to measure bone outcomes [39, 57]. The respective associations between the PA intensities and all reported bone outcomes can be found in Table 1.

**Table 2:** A summary of the bone imaging methods used and anatomical sites assessed in all studies included in the review (*N* = 30)

Method	Anatomical site	Frequency (%)	References
DXA		21 (70)	[39*], [40–43, 45, 47, 48, 50, 52–57*], [60, 61, 64–67]
	Whole body	12 (40)	[39*], [47, 52–57*], [60, 61, 66, 67]
	Femoral neck	11 (37)	[39*], [40–42, 45, 47, 52, 55, 64, 66, 67]
	Lumbar spine	8 (27)	[39*], [40, 47, 52, 55, 65–67]
	Hip	5 (17)	[39*], [45, 47, 50, 65]
	Trochanter	4 (13)	[41, 42, 47, 64]
	Intertrochanter	3 (10)	[41, 42, 47]
	Upper limbs	2 (7)	[54, 60]
	Lower limbs	2 (7)	[54, 60]
	Forearm	2 (7)	[39*], [48]
	Calcaneus	1 (3)	[48]
	Ward’s area	1 (3)	[43]
QUS		5 (17)	[44, 49, 58, 62, 63]
	Calcaneus	4 (13)	[44, 49, 58, 62]
	Mid-tibia	1 (3)	[63]
	Distal forearm	1 (3)	[63]
pQCT		6 (20)	[24, 39*], [46, 51, 57*], [59]
	50% tibia	3 (10)	[24, 46, 59]
	65% tibia	1 (3)	[39*]
	66% tibia	1 (3)	[51]
	4% tibia	1 (3)	[51]
	8% tibia	1 (3)	[59]
	20% tibia	1 (3)	[57*]
	4% radius	2 (7)	[39*], [51]
	65% radius	2 (7)	[39*], [51]

*DXA*, dual-energy x-ray absorptiometry; *QUS*, quantitative ultrasound; *pQCT*, peripheral quantitative computed tomography; *studies that used both DXA and pQCT to assess bone outcomes

Studies included in this review were required to have monitored habitual PA objectively using an accelerometer and to have reported both moderate and high intensities of activity. Accelerometer-derived methods used to collect and process this data are presented in Table 3. Fourteen of the included studies reported activity associations with MPA, MVPA and VPA; six reported MVPA and VPA; five reported MPA and VPA; and two studies reported MPA, MVPA, VPA and very vigorous PA (VVPA) (Table 3). Two studies [45, 46] did not report activity as MPA and/or MVPA *and* VPA, but instead used raw data and calculated the average number of acceleration peaks per day across different ‘impact bands’. Since the impact bands related to activities of various intensities (e.g. walking, brisk walking, running and jumping), these studies were included as it allowed an intensity comparison to be made between activities that are frequently classified elsewhere as MPA or VPA. One other study [48] reported various thresholds of activity intensity that were described as vigorous; however, since the lower thresholds were similar in magnitude to moderate intensity thresholds used in other studies already included in the review, this study was also included and an intensity comparison was made. Activity was reported as minutes per day in most (*n* = 24/30) studies [24, 39–43, 47, 49–55, 58–67], or as the proportion of recording time [44, 48, 56, 57], or number of peaks per day across various impact bands [45, 46].

**Table 3:** A summary of the accelerometer data collection and processing methods used in all studies included in the review (*N* = 30)

Method		Frequency (%)	References
Intensities reported	MPA, MVPA, VPA	14 (47)	[39, 41–44, 49, 53, 54, 56, 60, 62, 64, 66, 67]
	MVPA and VPA	6 (20)	[47, 51, 57, 59, 61, 65]
	MPA and VPA	5 (17)	[24, 40, 50, 52, 55]
	MPA, MVPA, VPA and VVPA	2 (7)	[58, 63]
	6 ‘VPA’ intervals	1 (3)	[48]
	3 impact bands (low, medium and high)	1 (3)	[46]
	6 impact bands (from brisk walking to jumping)	1 (3)	[45]
Accelerometer make and model†	Actigraph GT1M	14 (47)	[24, 41, 43, 44, 47, 49, 52, 56, 59, 63–67]
	MTI 7164	6 (20)	[24, 48, 50, 60, 61, 65]
	Actigraph GT3X	3 (10)	[44, 56, 66]
	Actigraph GT3X +	3 (10)	[53, 54, 58]
	Actigraph Actitrainer	3 (10)	[44, 49, 56]
	WAM 6471	2 (7)	[42, 55]
	Newtest monitor	2 (7)	[45, 46]
	Actiwatch motion sensor	1 (3)	[57]
	Actigraph wGT3X-BT	1 (3)	[51]
	Actical	1 (3)	[39]
	Lifecorder GS	1 (3)	[62]
	GENEActiv	1 (3)	[40]
Wear location	Right hip	18 (60)	[24, 39, 41–44, 49, 51–53, 55, 56, 60, 61, 63, 64, 66, 67]
	Lumbar spine/lower back	3 (10)	[47, 48, 57]
	Non-dominant wrist	2 (7)	[40, 54]
	Right waist	1 (3)	[62]
	Right iliac crest	1 (3)	[59]
	Waist midaxillary line	1 (3)	[50]
	NR	4 (13)	[45, 46, 58, 65]
Epoch length	60 s	11 (37)	[24, 42, 43, 49, 50, 55, 57, 60, 61, 65, 67]
	15 s	8 (27)	[39, 41, 44, 47, 52, 56, 59, 64]
	10 s	4 (13)	[48, 51, 53*], [63]
	5 s	2 (7)	[40*], [58]
	Raw data (no. of peaks)	2 (7)	[45, 46]
	2 min	1 (3)	[62]
	NR	2 (7)	[54, 66]
Definition of PA intensities	**MPA**, 2296–4011; **MVPA**, ≥ 2296; **VPA**, ≥ 4012 cpm	11 (37)	[41, 44, 49, 51^a^], [52^b^], [53^c^], [56, 59^a^], [61^a^], [64, 65^a^]
	**MPA**, 2000–3999; **MVPA**, ≥ 2000; **VPA**, ≥ 4000 cpm	4 (13)	[47^a^], [55^b^], [66, 67]
	**MPA**, 3600–6199; **MVPA**, ≥ 3600; **VPA**, ≥ 6200 cpm	2 (7)	[24^b^], [60]
	**MPA**, 1952–5724; **MVPA**, ≥ 1952; **VPA**, ≥ 5724 cpm	1 (3)	[43]
	**MPA**, 500–3999; **MVPA**, > 500; **VPA**,4000–7599 cpm	1 (3)	[58]
	**MPA**, 2000–2999; **MVPA**, ≥ 2000; **VPA**, ≥ 2999 cpm	1 (3)	[42]
	**MPA**, 1500–6500; **MVPA**, > 1500; **VPA**, > 6501 cpm	1 (3)	[39]
	Activity data categorised into 11 levels: **MPA**, levels 4–6; **MVPA**, levels 4–9; **VPA**, levels 7–9	1 (3)	[62]
	**MVPA**, > 500; **VPA**, > 1000 cpm	1 (3)	[57]
	**MPA**, 527–2818; **VPA**, ≥ 2818 counts	1 (3)	[50]
	**MPA**, 100 mg; **VPA**, 400 mg	1 (3)	[40]
	Threshold counts for different intervals: ≥ 3000, ≥ 4000, ≥ 5200, ≥ 6500, ≥ 7000, ≥ 8200 cpm	1 (3)	[48]
	6 impact bands: (1) 0.5–1.1 g; (2) 1.1–2.1 g; (3) 2.1–3.1 g; (4) 3.1–4.2 g; (5) 4.2–5.1 g; (6) > 5.1 g	1 (3)	[45]
	3 impact bands: 0.5–2.1 g; 2.1–4.2 g; > 4.2 g	1 (3)	[46]
	Age-specific cut-points for ENMO	1 (3)	[54]
	Age-appropriate thresholds derived from EE prediction equations	1 (3)	[63]
No. of days wear††	7 days	16 (53)	[24, 39, 43, 45–47, 51–54, 59–61, 63, 66, 67]
	4 days (2 WK and 2 WE)	5 (17)	[41, 42, 48, 55, 64]
	5 days (WK and WE days)	3 (10)	[44, 56, 65]
	4 days (1 WE)	2 (7)	[50, 65]
	5 days (1 WE)	1 (3)	[58]
	4–7 days (1 WE)	1 (3)	[40]
	2 days	1 (3)	[57]
	14 days	1 (3)	[62]
	NR	1 (3)	[49]
Definition of a valid day	≥ 10 h	15 (50)	[24, 39, 41–43, 52, 53, 55, 59–64, 66]
	≥ 8 h	8 (27)	[45–48, 50, 51, 65, 67]
	≥ 8 but ≤ 18 h	2 (7)	[44, 56]
	≥ 10 waking and ≥ 4 sleeping h	1 (3)	[54]
	≥ 6 h	1 (3)	[49]
	Daily activity from 6am to 8 pm	1 (3)	[58]
	NR	2 (7)	[40, 57]
Non-wear time	≥ 20 min consecutive 0 count	6 (20)	[44, 49, 51, 52, 56, 62]
	Diary of wear/non-wear	4 (13)	[45, 46, 54, 63]
	≥ 60 min consecutive 0 counts	2 (7)	[53, 59]
	≥ 10 min consecutive 0 counts	3 (10)	[42, 48, 61]
	All night activity (00:00–06:00) and ≥ 10 min consecutive 0 counts	2 (7)	[66, 67]
	≥ 30 min consecutive 0 counts	1 (3)	[64]
	All-day consecutive 0 counts	1 (3)	[39]
	NR	11 (37)	[24, 40, 41, 43, 47, 50, 55, 57, 58, 60, 65]
Minimum no. of valid days for inclusion	≥ 3 days	16 (53)	[24], [42^d^], [43, 44, 47, 48^d^], [49^d^], [50, 52^d^], [55, 56, 59, 60, 65, 66^d^], [67^d^]
	≥ 4 days	9 (30)	[39, 40^d^], [41^e^], [53^d^], [54^d^], [61^d^], [62^d^], [63^d^], [64^e^]
	≥ 2 days	4 (13)	[45, 46, 51^d^], [57]
	5 days	1 (3)	[58^d^]

*NR*, not reported; *MPA*, moderate physical activity; *MVPA*, moderate to vigorous physical activity; *VPA*, vigorous physical activity; *VVPA*, very vigorous physical activity; *EE*, energy expenditure; *mg*, milli-gravitational units; *WK*, weekday; *WE*, weekend day.

^†^NB: total *n* is greater than 30 as some studies used more than one model of the accelerometer to collect activity data. ^††^NB: total *n* is greater than 30 as some studies required participants to wear the accelerometer for a different number of days when collecting data at different time points; *from raw acceleration data; ^a^MVPA and VPA only; ^b^MPA and VPA only; ^c^linearly scaled to accommodate 10-s epochs; ^d^including ≥ 1 weekend day; ^e^including ≥ 2 weekend days.

#### Quality of included studies

The majority of included studies (*n* = 20/30) were awarded a ‘fair’ quality rating [24, 39–42, 44–56, 59, 60], with four studies being deemed to be of ‘good’ quality [64–67] and six studies deemed to be of ‘poor’ quality [43, 57, 58, 61–63]. Only 9/30 studies [39, 41, 53, 54, 58, 61–64] had a ‘yes’ response to item 9, where participants were required to have ≥ 4 days with ≥ 10 h of accelerometer data. Despite satisfying this requirement, four of these studies [58, 61–63] were still deemed to be of poor quality as they had not controlled for important covariates (e.g. age, sex, ethnicity, maturational stage, skeletal/body size) in the analyses when investigating independent associations between bone outcomes and each activity intensity (MPA and/or MVPA *and* VPA). Others [57] also failed to adjust for appropriate covariates, and one study [43] was deemed to be of poor quality as the outcome measure used (proximal femur shape variation) was reported as having not been used in the area of bone health and therefore was not considered a valid and reliable outcome. Information on individual study ratings can be found in Online Resource 2.

### Accelerometer data collection and processing methods used in all included studies

The accelerometer data collection and processing methods used in the included studies (*n* = 30) are detailed in Table 3. There was considerable variability between studies for all aspects reviewed.

Most studies (*n* = 24/30) used one model of an accelerometer to collect PA data; however, six studies [24, 44, 49, 56, 65, 66] used two or three different models (participants only wore one of the two/three models at a time) to obtain data and appear multiple times in this section of Table 3. The most commonly used accelerometer was the Actigraph GT1M (14/30 studies), followed by the MTI 7164 (6/30 studies). Other models included the Actigraph GT3X (*n* = 3/30), GT3X + (*n* = 3/30), Actitrainer (*n* = 3/30), wGT3X-BT (*n* = 1/30), the WAM 6471 (*n* = 2/30), Newtest monitor (*n* = 2/30), Actiwatch motion sensor (*n* = 1/30), Actical (*n* = 1/30), Lifecorder GS (*n* = 1/30) and GENEActiv (*n* = 1/30). Monitors were most commonly worn on the right hip, with 18/30 studies using this wear location. Other studies required participants to wear the accelerometer on the lumbar spine/lower back (*n* = 3/30), right waist (*n* = 1/30), waist midaxillary line (*n* = 1/30), right iliac crest (*n* = 1/30) or non-dominant wrist (*n* = 2/30). Four studies did not report the accelerometer wear location.

Epoch length ranged from 5 to 120 s, with 60 s being used most commonly in 11/30 studies, with the next most common being 15 s used in 8/30 studies. Three studies [42, 57, 60] did not report epoch length; however, activity was referred to in terms of counts per minute in the methods, so it was assumed that a 60-s epoch had been used. McCormack and colleagues [53] and Bielemann and colleagues [40] collected raw data that was then integrated into 10-s and 5-s epochs. Two studies [45, 46] did not use epochs and instead collected raw data and calculated the number of peaks that occurred each day within certain bands of acceleration that corresponded with peaks in impact typically encountered when exposed to different types of activity.

There was large variability in the ways in which studies defined the PA intensities of interest (Table 3). Eleven studies defined MPA and/or MVPA and VPA using the Evenson [68] cut-points of 2296–4011 cpm, ≥ 2296 cpm and ≥ 4012 cpm, respectively. Cut-points for MVPA ranged from > 500 to ≥ 3600 cpm and for VPA > 1000 to > 6500 cpm (Table 3). Not all cut-points were defined in terms of counts per minute. For example, Deere et al. [45, 46] separated raw acceleration data into different impacts using ‘*g*’ bands, where 1* g* is equivalent to gravitational force. Six impact bands relating to normal walking, brisk walking, jogging/running and jumping were used in [45] and activity was defined as low (0.5–2.1* g*), medium (2.1–4.2* g*) and high (> 4.2* g*) impact in [46]. One study [48] reported thresholds of ≥ 3000, ≥ 4000, ≥ 5200, ≥ 6500, ≥ 7000 and ≥ 8200 cpm. The ≥ 3000 cpm was comparable to thresholds used to define MVPA in other studies, so was used as MVPA, with all others treated as VPA.

The number of days participants were required to wear the accelerometer ranged from 2 days to  2 weeks, with most studies requesting participants to wear the monitor for 7 days (16/30 studies). Four days (including 2 week and 2 weekend days) were required in 5/30 studies and with one weekend day in 2/30 studies. Five days with both weekend days (3/30 studies) or one weekend day (1/30 studies) were also used.

Valid day definitions varied from 6 to 14 h (daily activity from 6am to 8 pm; Table 3). Most commonly, a valid day was defined as having at least 10 h wear (15/30 studies), followed by at least 8 h of wear (8/30 studies). Non-wear was defined as periods of at least 10 min of consecutive zero counts in 5/30 studies (two of these studies also removed night activity), with periods of 20 (1/30 studies), 30 (1/30 studies) and 60 (1/30 studies) min of consecutive zero counts, and all-day consecutive zero counts (1/30 studies) also being used. Participants were asked to complete a diary of when the monitor was worn/removed in 4/30 studies. A large proportion of studies (*n* = 11/30) did not report how non-wear was defined.

The minimum number of days required for including participants’ accelerometer data in analyses ranged from at least 2 (4/30 studies) to 5 days (1/30 studies), with most requiring a minimum of 3 valid days (16/30 studies). Nine studies required participants to have at least 4 valid days to be included in the final sample. Many studies did not specify whether included days were required to be week and/or weekend days, whilst others required participants to have at least one or both weekend days in order to satisfy the inclusion criteria (Table 3).

### Associations of MPA and/or MVPA and VPA with bone outcomes

**Fig. 2:**
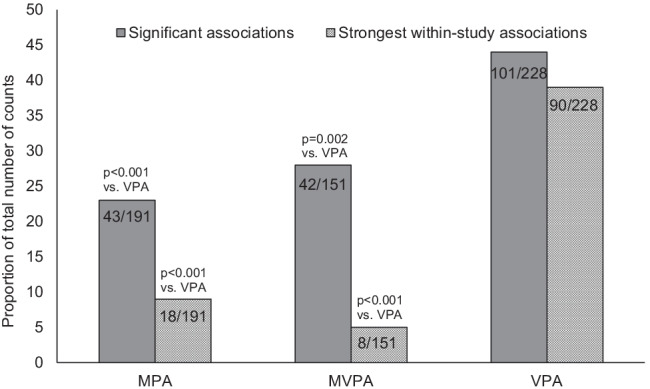
Overall significant counts (stage 1) and ‘strongest within-study association’ counts (stage 2) expressed as a proportion of the total number of counts available for each intensity (total counts are the number of counts available, regardless of statistical significance; MPA, moderate physical activity; MVPA, moderate-to-vigorous physical activity; VPA, vigorous physical activity). In stage 1, votes were counted based on whether each intensity was statistically significant (*p* < 0.05; 1 = yes). In stage 2, out of the significant intensities within a study, only the intensity with the largest effect size (association) received a vote (only 1 count available out of the 2/3 intensities). Votes were counted for all analyses for each outcome included in a study (e.g. for the whole sample, boys and girls). When the value of association was the same for two intensities, votes were counted if significant in stage 1, but a stage 2 vote was not cast. When negative associations were observed, their significance was noted but again, no stage 2 vote was cast. *p*-values (vs VPA) represent the Bonferroni-adjusted *p*-values from the 2 × 2 chi-square tests for ‘MPA vs VPA’ and ‘MVPA vs VPA’ when the omnibus 3 × 2 chi-square test indicated that there was a significant difference between at least two of the three intensities. Significance was set at the 5% level

The results for the vote count, conducted as a semi-quantitative alternative to a meta-analysis, are presented in Table 4. A more detailed reporting of each outcome at each site is given in Online Resource 3. Overall, there were 570 association analyses performed between a PA intensity and a bone outcome, (all bone measurement methods: all anatomical sites) of which 33% (186/570) were statistically significant (*p* < 0.05). The chi-square tests provided very strong evidence that this proportion of significant associations differed depending on the PA intensity (3 × 2 *χ*^2^ = 24.6, *p* < 0.001) and that it was significantly higher for VPA (44%: 101/228) than for MVPA (28%: 42/151, 2 × 2 *χ*^2^ = 10.5, *p* = 0.002) and MPA (23%: 43/191, 2 × 2 *χ*^2^ = 21.9, *p* < 0.001). From the within-study comparisons (where the PA intensity with the strongest association with the bone outcome received a count), the chi-square tests provided very strong evidence that the proportion of ‘strongest association’ counts differed by PA intensity (3 × 2 *χ*^2^ = 86.6, *p* < 0.001) and that it was higher for VPA (39%: 90/228) than for MVPA (5%: 8/151, 2 × 2 *χ*^2^ = 55.3, *p* < 0.001) and MPA (9%: 18/191, 2 × 2 *χ*^2^ = 49.1, *p* < 0.001) The overall ‘all bone methods: all sites’ proportions of ‘statistically significant association’ total counts and within-study ‘strongest association’ counts for each intensity are displayed in Fig. 2. Repeating the vote count after the removal of poor-quality studies (a sensitivity analysis) did not influence the overall findings. The proportion of significant and most strongly associated counts for each intensity remained very similar, with VPA still having a higher proportion of ‘strongest association’ counts compared to the other intensities. Repeating the analyses using only studies that had reported all three intensities (MPA, MVPA and VPA) also obtained the same pattern of results.

**Table 4:** Results from the vote count for all included studies by imaging method (DXA, pQCT and QUS) and each anatomical site assessed. In stage 1, votes were counted based on whether each intensity was statistically significant (*p* < 0.05; 1 = yes). In stage 2, out of the significant intensities, only the intensity with the largest effect size (association) received a vote (only 1 count available out of the 2/3 intensities). Votes were counted for all analyses for each outcome included in a study (e.g. for the whole sample, boys and girls). When the value of association was the same for two intensities, votes were counted if significant in stage 1, but a stage 2 vote was not cast. When negative associations were observed, their significance was noted but again, no stage 2 vote was cast. Results are presented as the proportion of significant/most strongly associated counts out of the total number of counts available for each intensity (total counts are regardless of statistical significance), followed by the number of significant/most strongly associated counts and the number of total counts available for each intensity (% (*n*/*N*))

	**Stage 1:** PA associations that were statistically significant (p < 0.05)			**Stage 2:** PA associations that were the strongest within a study		
	MPA sig. % (*n*/*N*)	MVPA sig. % (*n*/*N*)	VPA sig. % (*n*/*N*)	MPA strongest assoc. % (*n*/*N*)	MVPA strongest assoc. % (*n*/*N*)	VPA strongest assoc. % (*n*/*N*)
**All bone methods: all sites**	**23% (43/191)**	**28% (42/151)**	**44% (101/228)**	**9% (18/191)**	**5% (8/151)**	**39% (90/228)**
***p*-value (vs VPA)**	***p* < 0.001**	***p* = 0.002**	**-**	***p* < 0.001**	***p* < 0.001**	**-**
**DXA: all sites**	**30% (34/114)**	**33% (32/98)**	**50% (68/136)**	**14% (16/114)**	**5% (5/98)**	**43% (59/136)**
***p*-value (vs VPA)**	***p* = 0.002**	***p* = 0.016**	**-**	***p* < 0.001**	***p* < 0.001**	**-**
Whole body	30% (7/23)	25% (6/24)	32% (9/28)	22% (5/23)	0% (0/24)	11% (3/28)
Lumbar spine	8% (1/13)	9% (1/11)	24% (4/17)	8% (1/13)	0% (0/11)	24% (4/17)
Hip	54% (7/13)	33% (2/6)	100% (18/18)	0% (0/13)	0% (0/6)	94% (17/18)
Femoral neck^a^	24% (8/33)	32% (6/19)	63% (22/35)	9% (3/33)	11% (2/19)	60% (21/35)
Trochanter	14% (1/7)	11% (1/9)	44% (4/9)	14% (1/7)	11% (1/9)	44% (4/9)
Intertrochanter	20% (1/5)	14% (1/7)	57% (4/7)	20% (1/5)	14% (1/7)	57% (4/7)
Ward’s area	50% (1/2)	50% (1/2)	0% (0/2)	50% (1/2)	0% (0/2)	0% (0/2)
Upper limbs	25% (2/8)	75% (6/8)	38% (3/8)	0% (0/8)	13% (1/8)	38% (3/8)
Lower limbs	75% (6/8)	75% (6/8)	25% (2/8)	50% (4/8)	0% (0/8)	13% (1/8)
Calcaneus	-	100% (1/1)	100% (1/1)	-	0% (0/1)	100% (1/1)
Distal forearm	0% (0/2)	33% (1/3)	33% (1/3)	0% (0/2)	0% (0/3)	33% (1/3)
**pQCT: all sites**	**7% (4/57)**	**9% (3/33)**	**28% (20/72)**	**4% (2/57)**	**3% (1/33)**	**28% (20/72)**
***p*-value (vs VPA)**	***p* = 0.005**	*p* = 0.063	**-**	***p* < 0.001**	***p* = 0.007**	**-**
Tibial shaft^b^	4% (2/46)	15% (2/13)	33% (17/52)	0% (0/46)	8% (1/13)	33% (17/52)
Distal tibia^c^	**-**	0% (0/7)	29% (2/7)	**-**	0% (0/7)	29% (2/7)
Radius shaft^d^	0% (0/7)	0% (0/8)	13% (1/8)	0% (0/7)	0% (0/8)	13% (1/8)
Distal radius^e^	50% (2/4)	20% (1/5)	0% (0/5)	50% (2/4)	0% (0/5)	0% (0/5)
**QUS: all sites**	**25% (5/20)**	**35% (7/20)**	**65% (13/20)**	**0% (0/20)**	**10% (2/20)**	**55% (11/20)**
***p*-value (vs VPA)**	***p* = 0.022**	*p* = 0.116	**-**	*p < 0.001	*p = 0.011	**-**
Calcaneus	19% (3/16)	31% (5/16)	69% (11/16)	0% (0/16)	6% (1/16)	63% (10/16)
Midshaft tibia	100% (2/2)	100% (2/2)	100% (2/2)	0% (0/2)	50% (1/2)	50% (1/2)
Distal 1/3 radius	0% (0/2)	0% (0/2)	0% (0/2)	0% (0/2)	0% (0/2)	0% (0/2)

*n* number of significant/most strongly associated counts for each intensity; *N* total number of counts available for each intensity (regardless of significance). ^a^Femoral neck consists of counts for the whole femoral neck, as well as the femoral neck subregions (superlateral FN and inferomedial FN); ^b^tibial shaft includes the 66%, 65% and 50% sites; ^c^distal tibia includes the 4%, 8% and 20% sites; ^d^radius shaft includes the 65% site; ^e^distal radius includes the 4% site.

*p*-value (vs VPA) = these are the Bonferroni-adjusted *p*-values from the 2 × 2 chi-square tests (*Fisher’s exact test) for ‘MPA vs VPA’ and ‘MVPA vs VPA’ when the omnibus 3 × 2 chi-square test (*Fisher’s exact test) indicates that there is a significant difference between at least two of the three intensities. The *p*-values in bold font indicate significance at the 5% level.

#### DXA: all sites

Overall, there were 348 association analyses of DXA-derived bone outcomes (DXA: all sites), 39% (134/348) of which were statistically significant. The chi-square tests provided strong evidence that this proportion of statistically significant associations differed depending on the PA intensity (3 × 2 *χ*^2^ = 12.6, *p* = 0.002) and that it was significantly higher for VPA (50%: 68/136) than for MVPA (33%: 32/98, 2 × 2 *χ*^2^ = 7.0, *p* = 0.016) and MPA (30%: 34/114, 2 × 2 *χ*^2^ = 10.5, *p* = 0.002). From the within-study comparisons, the chi-square tests provided very strong evidence that the proportion of ‘strongest association’ counts differed by PA intensity (3 × 2 *χ*^2^ = 54.8, *p* < 0.001) and that it was higher for VPA (43%: 59/136) than for MVPA (5%: 5/98, 2 × 2 *χ*^2^ = 42.0, *p* < 0.001) and MPA (14%: 16/114, 2 × 2 *χ*^2^ = 25.4, *p* < 0.001).

At the whole body, 30% (7/23), 25% (6/24) and 32% (9/28) of counts were significant for MPA, MVPA and VPA, respectively. Of the 12 studies reporting bone outcomes at this site, six reported no significant associations with PA [39, 47, 56, 57, 66, 67]. Significant, positive associations were reported between MPA and BMC [53] and VPA and BMC [52, 55], with a significant, negative correlation also reported between VPA and BMC (*r* =  − 0.21, *p* < 0.05) [61]. All activity intensities (MPA, MVPA and VPA) were significantly associated with BMC and BMD in overweight/obese 8–12-year-olds with low adherence to the Mediterranean diet pattern (MDP) [54]. The MVPA and VPA *β* coefficients were very similar (BMC: *β* = 0.109 and 0.108 for MVPA and VPA; BMD: *β* = 0.185 and 0.183), so stage 2 counts were not determined. Tobias and colleagues [60] also found MPA, MVPA and VPA to be significantly associated with BMC, BMD and BA in 4457, 11-year-olds. In all instances, the regression coefficient was largest for MPA. The thresholds used to define activity intensities were much higher (MPA 3600–6199 cpm, MVPA ≥ 3600 cpm, VPA ≥ 6200 cpm) than other studies reporting strongest associations with VPA (MPA 2000–3999 cpm, VPA ≥ 4000 cpm in [55]; MVPA ≥ 2296 cpm, VPA ≥ 4012 cpm in [52]) and only 3–4 min/day of VPA were reported in comparison to 20–30 min/day [52, 55]. At the whole body, the proportion of counts most strongly associated with bone outcomes was 22%, 0% and 11% for MPA, MVPA and VPA, respectively (Table 4).

At the lumbar spine, 8% (1/13), 9% (1/11) and 24% (4/17) of MPA, MVPA and VPA counts were significant. Of the eight studies assessing bone outcomes at this site, five did not report any significant associations with PA [39, 47, 55, 66, 67]. One study [52] reported a significant, positive correlation between BMC and VPA, with another reporting significant associations between BMD and VPA, and BMD and MPA in 18-year-old boys and girls, respectively [40]. A longitudinal study found both MVPA and VPA from age 5 to 15 years significantly predicted lumbar spine BMC in boys (*β* estimate largest for VPA), but only VPA was significant in girls [65]. At the lumbar spine, the proportion of counts most strongly associated with bone outcomes was 8%, 0% and 24% for MPA, MVPA and VPA, respectively.

In comparison to the whole body and lumbar spine, a higher proportion of counts were significant for MPA (54% (7/13)), MVPA (33% (2/6)) and VPA (100% (18/18)) at the hip. Of the five studies reporting bone outcomes at this site, significant, positive associations were reported between BMD and VPA in 15-year-olds [47], and with BMD and impacts of 4.2–5.1 g and > 5.1 g (equivalent to running and jumping, counted as VPA) in 17.7-year-old adolescents [45]. Moderate-to-vigorous PA [65] and VPA [39, 47, 65] were also significantly associated with BMC, with VPA most strongly associated compared to MVPA for both boys and girls in the study assessing PA and BMC at ages 5, 8, 11, 13 and 15 [65]. In a cross-sectional study conducted in the same cohort [50], geometric indices obtained from hip structural analysis at age 5 were significantly correlated with MVPA in girls and VPA in both boys and girls. None of the significant MPA or MVPA counts was most strongly associated with outcomes at the hip whereas 94% of VPA counts were most strongly associated at this site. The amount of VPA reported in these studies ranged from around 2 to 40 min per day. Studies also assessed outcomes at the trochanter, intertrochanter and Ward’s area regions of the hip (counts for each in Table 4). Significant, positive associations were observed between BMD and MPA in boys [64], MVPA in boys and VPA in girls [41] and VPA in both boys and girls [42] at the trochanter, and between BMD and VPA [42, 47] and BMD and MPA in girls and MVPA in boys [41] at the intertrochanter. Shape variation in Ward’s area was significantly associated with MPA and MVPA (most strongly with MPA) in 9–10-year-old boys, but not girls [43].

At the femoral neck, 24% (8/33), 32% (6/19) and 63% (22/35) of total MPA, MVPA and VPA counts, respectively, were significant. Of the eleven studies assessing outcomes at this site, one did not report significant associations (including at the superlateral and inferomedial subregions) [64]. Significant, positive associations were reported between composite strength indices and BMC with VPA in 9–10-year-old boys and girls [55]. Significant associations were also reported for BMC and MPA and VPA (strongest for VPA) in 11–13-year-old boys [52]. A longitudinal study using boys from the same cohort (11–13 years at baseline) found VPA significantly predicted BMC over a 12-month period [67]. One study reported significant associations between BMC and MPA and MVPA (strongest for MPA), but not VPA in 10-year-olds [39]. A higher VPA cut-point of 6500 cpm was used compared to ~ 4000 cpm in the studies reporting significant, strongest associations with VPA and only 2 min/day compared to ~ 10–30 min/day at respective intensities were reported. Significant associations were also reported between BMD and MPA [52], MVPA [41, 47, 67] and VPA [42, 47, 52, 67], with VPA being most strongly associated in the studies where MPA [52] and MVPA [67] were also significant. One study [47] used receiver operating characteristic curve analysis to assess the relationship between MVPA, VPA and BMD. Since more than 32 min/day of VPA was associated with increased BMD, compared to 78 min/day of MVPA, votes were counted in favour of VPA. A longitudinal study conducted in boys found that VPA (not MPA or MVPA) during the pubertal years significantly predicted BMC and BMD at 18 years [66]. Significant associations between BMD and MPA in both boys and girls, and VPA in boys [40] and impacts of 4.2–5.1 g and > 5.1 g (equivalent to running and jumping, counted as VPA) [45] were also reported in older adolescents aged ~ 18 years. Impacts of 4.2–5.1 g and > 5.1 g were also significantly associated with geometric and strength indices [45]. At the femoral neck, the proportion of counts most strongly associated with bone outcomes was 9%, 11% and 60% for MPA, MVPA and VPA, respectively.

Other sites assessed using DXA included the upper limbs, lower limbs calcaneus and forearm (counts for each in Table 4). Both MVPA and VPA were significantly associated with BMC, BMD and BA (all *β*’s largest for VPA) in the upper limbs of 12-year-olds from the ALSPAC cohort. In the lower limbs, BMC, BMD and BA were significantly associated with MPA and MVPA, but not VPA (all *β*’s largest for MPA). Munoz-Hernandez et al. [54] reported significant associations between BMC and BMD with MPA and MVPA (same β, no stage 2 vote), but not VPA at the upper limbs and with MPA, MVPA and VPA at the lower limbs (BMC no stage 2 vote, BMD β largest for VPA), but only in those with low adherence to the MDP. Hasselstrom [48] assessed BMD of the calcaneus and distal forearm and reported significant associations with BMD for all intensities of activity (> 3000, > 4000, > 5200, > 6500, > 7000 and > 8200 cpm). The beta was largest for the > 6500 and > 7000 cpm thresholds at the calcaneus and at the forearm, and the beta was similar from the > 5200 cpm threshold onwards; therefore, VPA was deemed most strongly associated. One study [39] did not report any significant associations between forearm BMC and MPA, MVPA or VPA.

#### pQCT: all sites

Overall, there were 162 association analyses of pQCT derived bone outcomes (pQCT: all sites), 16% (27/162) of which were statistically significant. The chi-square tests provided strong evidence that this proportion of significant associations differed depending on the PA intensity (3 × 2 *χ*^2^ = 11.6, *p* = 0.003) and that it was significantly higher for VPA (28%: 20/72) than for MPA (7%: 4/57, 2 × 2 *χ*^2^ = 9.1, *p* = 0.005) though only borderline significantly higher than for MVPA (9%: 3/33, 2 × 2 *χ*^2^ = 4.6, *p* = 0.063). From the within-study comparisons, the chi-square tests provided very strong evidence that the proportion of ‘strongest association’ counts differed by PA intensity (3 × 2 *χ*^2^ = 19.6, *p* < 0.001) and that it was higher for VPA (28%: 20/72) than for MVPA (3%: 1/33, 2 × 2 *χ*^2^ = 8.7, *p* = 0.007) and MPA (4%: 2/57, 2 × 2 *χ*^2^ = 13.2, *p* < 0.001). Several outcomes were reported at each site and are detailed in Online Resource 3.

At the distal tibia, only bone strength index (BSI) was significantly associated with VPA [51, 59]. At the tibial shaft, polar strength-strain index (SSIp) was significantly associated with MVPA in 15-year-olds [59] and both MVPA and VPA (strongest for VPA) in 11-year-olds [51]. Significant associations between VPA and cortical BMC, BA and periosteal circumference were observed at the mid-tibia in 1748 participants from the ALSPAC 15.5 year clinic [24]. In this study, significant negative associations were also observed between VPA and cortical BMC and endosteal circumference. At the 17-year ALSPAC clinic [46], high-impact activity > 4.2 g (equivalent to fast running, treated as VPA) was significantly associated with periosteal circumference, SSI and cross-sectional moment of inertia in boys. Although not significant in girls, there was a trend for a high-impact activity to have a larger beta coefficient. At the radius, one study did not report any significant associations between MVPA or VPA and outcomes at the 4% or 65% sites [51]. Another reported significant correlations between MPA and total bone density and BSI at the 4% site and VPA and cortical area at the 65% site [39].

#### QUS: all sites

Overall, there were 60 association analyses of QUS derived bone outcomes (QUS: all sites), 42% (25/60) of which were statistically significant. The chi-square tests provided moderate evidence that this proportion of significant associations differed depending on the PA intensity (3 × 2 *χ*^2^ = 7.1, *p* = 0.028) and that it was significantly higher for VPA (65%: 13/20) than for MPA (25%: 5/20, 2 × 2 *χ*^2^ = 6.5, *p* = 0.022) though not MVPA (35%: 7/20, 2 × 2 *χ*^2^ = 3.6, *p* = 0.116). Fisher’s exact tests were used for the within-study comparisons as the data violated one of the assumptions for chi-square tests (one cell count = 0). The Fisher’s exact tests provided very strong evidence that the proportion of ‘strongest association’ counts differed by PA intensity (3 × 2, *p* < 0.001) and that it was higher for VPA (55%: 11/20) than for MVPA (10%: 2/20, 2 × 2, *p* = 0.011) and MPA (0%: 0/20, 2 × 2, *p* < 0.001).

No significant associations were observed at the distal 1/3 radius [63]. At the calcaneus, MPA, MVPA and VPA were significantly associated with stiffness index (SI) in pre- and primary-school-aged children (both *β*’s largest for VPA) [44, 49], and in 10.8-year-old boys (*β* largest for MVPA), but not girls [62]. One study found all activity intensities (MPA, MVPA, VPA) were significantly associated with calcaneal broadband ultrasound attenuation (BUA; strongest for VPA) [44], with another [58] reporting significant associations between VPA and BUA, speed of sound (SOS) and bone quality index (BQI) in 10–12-year-old boys, but not girls. At the midshaft tibia, SOS was significantly correlated with MPA, MVPA and VPA in normal weight and overweight girls (10 years) and adolescents (15 years) combined (*r* largest for VPA at the non-dominant limb) [63]. Studies where VPA was most strongly associated with bone outcomes reported around 2–20 min/day of VPA.

#### Additional vote count by epoch length

The epoch length applied to accelerometer data has been shown to dramatically alter the PA data obtained. This is particularly prominent for VPA, where in children, it has been shown that around four times more VPA is identified when activity is assessed using a 5-s epoch compared to a 60-s epoch [69]. To investigate whether the intensity with the highest proportion of counts most strongly associated with bone outcomes differed depending on epoch length, additional vote counting was conducted separating studies into those who had used a ≥ 60-s epoch, and those using 15 s or less. Regardless of whether ≥ 60-s or ≤ 15-s epochs were used, a higher proportion of counts were significant for VPA compared to MPA or MVPA (Online Resource 3) and a higher proportion of the total counts for VPA were identified as being most strongly associated with bone outcomes compared to the other activity intensities.

## Discussion

This systematic review summarises the range of accelerometry data collection and processing methods used to estimate habitual PA in relation to bone outcomes in children and adolescents and, irrespective of the range of methods used, identifies whether a particular intensity of habitual PA (moderate (MPA/MVPA) or vigorous (VPA)) is more strongly associated and beneficial to bone health in this population. Considerable heterogeneity in the accelerometry methods of reviewed studies was observed. Studies varied in terms of the monitor make and model used, wear criteria applied (definition of a valid day, non-wear time within a day, number of valid days required for inclusion), accelerometer output (raw or proprietary count-based), whether the output was averaged over an epoch, and if so, the length of epoch, and in the cut-points used to determine the activity intensity classifications. Regardless of the accelerometry methods employed, results were still indicative of a greater benefit of VPA over MPA/MVPA; however, the variability in accelerometer methods meant it was not possible to identify the precise amount of VPA (a key component of PA dose) required to benefit bone.

Habitually performed VPA was significantly and positively associated with several bone outcomes (bone mineral content and density, geometric and strength indices) at important load bearing sites such as the hip and femoral neck. Associations between VPA and bone outcomes were often larger in comparison to MPA/MVPA, such that, for the same increase in the amount of time spent in each intensity, VPA would lead to greater gains in bone outcomes. Studies that conducted regression analyses where activity intensities were entered simultaneously into the model also demonstrated evidence of a threshold effect whereby lower intensities of activity no longer had any explanatory power once VPA/high-impact activity was included in the models [42, 45, 46]. However, variability in sample characteristics, the imaging method used to obtain bone outcome data, the anatomical sites assessed and range of bone outcomes reported at these sites, as well as differences in the ways in which accelerometer-derived habitual PA data was collected and processed, make it difficult to fully understand the relationship between VPA and bone. It is therefore not possible to identify the precise amount of VPA required to benefit bone health in this population.

In studies that observed significant, positive associations between habitual VPA and bone, the mean amount of time reportedly spent in VPA varied between around 2 and 40 min per day (Table 1). Large variability in the amount of reported VPA leads to a high level of uncertainty surrounding the recommended dose of bone-relevant PA in children and adolescents and prevents clear bone-specific activity recommendations from being made. Since the samples in these studies ranged from 5 to 18 years in age, differences in the amount of VPA reported could also be a reflection of the precipitous decline in habitual PA that occurs throughout adolescence [70]. However, even in studies with comparable sample characteristics, there was still considerable variability in the amount of VPA reported. For example, Sayers et al. [24], Janz et al. [65] and Gracia-Marco et al. [47] all described the VPA of 15-year-old boys and girls and reported around 3, 10 and 30 min per day. Differences were also observed in studies that both analysed 5-year-old participants from the Iowa Bone Development Study. One reported 38 and 28 min of VPA per day in boys and girls [50] compared to only 13 and 10 min per day in the other [65]. Differences in VPA prevalence are likely contributed to by large variations in accelerometer-derived measures of bone-specific habitual activity including (but not limited to) the processing methods such as choice of cut-point, epoch length and wear/non-wear criteria.

The studies included in the present review employed a diverse range of intensity thresholds to define MPA and/or MVPA and VPA. Vigorous PA was defined as being as low as > 1000 cpm to as high as > 6500 cpm, with many studies using a cut-point of ≥ 4000 or ≥ 4012 cpm (16/30 studies). Despite being designed and validated to reflect the same physiological intensity of activity (cardiovascular demand), the use of different cut-points to classify accelerometer outputs inevitably produces large differences in the estimates of activity behaviour [71, 72]. In addition to influencing the amount of time spent in VPA, differences in the cut-points used may have also influenced the intensity of activity identified as being most beneficial to bone outcomes. For example, Tobias et al. [60] found MPA, but not VPA to be significantly and most strongly associated with total body and lower limb BMC, BA, BMD and aBMC in 12-year-old children from the ALSPAC cohort. However, the cut-points used to define MPA and VPA were substantially larger (MPA, 3600–6199 cpm; VPA, ≥ 6200 cpm) than those used in other studies, and the MPA cut-point was similar in magnitude to how most other studies had defined VPA (≥ 4000 or ≥ 4012 cpm). In addition to the very high VPA threshold, the accelerometer output in this study was averaged over a 60-s epoch. As very high-intensity PA occurs in brief, sporadic episodes [73, 74], the averaging of PA data over a 60-s timeframe causes this activity to be misclassified as lower intensity activity and is likely the reason why only a small amount of VPA ≥ 6200 cpm was reported (~ 3 min/day).

With the exception of two studies that reported the number of peaks occurring each day within different impact bands from raw accelerometry data, the majority of reviewed studies reported accelerometer output (raw or count-based) over a range of epoch lengths. Epochs ranged from 5 to 120 s in duration, with 60 s being used most commonly in 11/30 studies (8/30 used 15 s, 4/30 10 s, 2/30 used 5 s). As epoch length increases, less time spent in VPA is reported due to the increased dilution of high-intensity activity amongst longer periods of lower intensity activity [71]. Significant differences in activity prevalence exist between shorter (5 s, 10 s, 15 s) and longer 60-s epochs [69, 72, 75] meaning it is not possible to compare the amount of activity accumulated between studies when such a large range of epoch lengths are used [75]. Variability in the length of epoch used therefore also contributes to the inability to recommend precisely how much time spent in VPA is required to be of benefit to bone. Whilst the shorter epochs of 5–15 s mean that activity data is averaged over a much smaller timeframe compared to 60 s and therefore less dilution of high-intensity activity is likely to occur, significant differences in the time spent in VPA have still been detected between epochs of 1, 5 and 15 s [71]. This is likely due to the fact that the vast majority (93%) of VPA bouts in children last for less than 10 s [74], with the mean duration of VPA bouts being only 3 s [73, 74]. Therefore, even when shorter epochs are used, over-smoothing of the short, sporadic, high-impact events most relevant to bone will still occur as the epoch length remains longer than the bout of activity being measured. Consequently, important characteristics of habitual VPA will almost certainly be misclassified as lower intensity activity even if studies use 15-s or 10-s epochs.

The number of studies using accelerometers to measure PA in children and adolescents has greatly increased; however, a lack of standardisation means the methods employed are very diverse, reducing the comparability of findings [23]. In addition to cut-points and epoch length, comparability between findings of reviewed studies is further hindered by the fact that the majority used monitors that output data in proprietary counts. These are device and manufacturer-specific, and since no standard exists for producing these units of measure across device manufacturers, it continues to be unclear as to what a ‘count’ means, both physically and physiologically [76]. Studies also varied greatly in terms of wear criteria and only a small number (9/30) required participants to have a minimum of 4 valid days, with at least 10 h of recording per day to be included. Since the number of days needed to achieve a reliability of 80% ranges from 4 to 9 in children and adolescents [34] and at least 10 h of recording per day is required to satisfy minimum wear time criteria to monitor daily exposure to PA [22], it raises questions as to whether the PA reported in included studies is representative of children’s habitual PA. Furthermore, not all studies specified whether the days sampled or included were week and/or weekend days, and since activity behaviour varies between these, both should be included [34]. The majority of studies used hip-worn accelerometers. Whilst these are thought to provide the most accurate estimation of activity intensity [77], wear compliance is significantly less in comparison to wrist-worn monitors [78]. Only two studies used a wrist-worn accelerometer; however, they should be considered in future studies to ensure greater wear and more representative data is obtained [78].

The variability of accelerometer data collection and processing methods used in the reviewed studies make it likely that the VPA reported is not reflective of levels habitually performed by children and adolescents. However, the lack of a gold standard comparison makes it impossible to know which methods provide the most valid estimates of activity [71]. Due to the intermittent, transient nature of children’s PA patterns [73, 74] and the fact that short, dynamic bursts of high-intensity activity are required to initiate osteogenesis [27], the use of shorter epochs (such 1 s) should be explored when investigating bone-relevant PA in free-living situations. Since jumping activities that are of benefit to bone generally last less than 1 s in duration, others have also suggested the use of the raw acceleration signal [79]. Two of the included studies [45, 46] conducted in the ALSPAC cohort used raw acceleration and computed the number of peaks that occurred within various impact bands using custom-designed code. High-impact PA > 4.2 g (jumping and running > 10 km/h) was identified as being most beneficial to hip BMD and structure in adolescents [45]. Raw acceleration is more reflective of the ground reaction forces experienced in everyday life and is better able to capture brief, sporadic PA episodes than epoch data [80]. Alternative accelerometer outputs that count the peaks within impact categories [45, 46] or that quantify daily loading into a score based on the osteogenic index [81] from raw accelerometry data are therefore likely to be more suited to evaluating PA in relation to bone health than currently used methods. However, limited information is currently available to interpret and infer activity type from raw metrics—an important characteristic for prescribing doses of activity and there are also several analytic and logistic challenges regarding the transmission and storage of large volumes of data and appropriate modelling methods, with raw-data-based analytical methods still in the process of being developed and optimised [82].

Several previous reviews that have included observational studies investigating PA in relation to bone have mostly included studies that used self-reported methods to assess habitual activity behaviour. A strength of the present review is that all included studies had objectively assessed habitual PA using accelerometry, which overcomes several limitations of self-report methods particularly when the population of interest is children. A more recent systematic review that focused on accelerometer-derived PA in relation to bone reported that MPA and VPA were the PA intensities that positively influenced bone outcomes in children and adolescents [25]. However, they did not compare the magnitude of associations and whether there was evidence of a greater benefit of one intensity over the other. The present review included studies that had performed analyses with activity data stratified by intensity (MPA/MVPA and VPA) and examined the magnitude of associations within each study, which allowed the contributions of higher intensity activity (which is reported as being most relevant to bone) to be assessed in more detail. Including all types of bone outcomes from several imaging methods meant that information on important indices of bone health that are not obtained through DXA could also be reported. However, studies that used three-dimensional techniques such as pQCT that distinguishes between trabecular and cortical bone and is able to assess important geometric indices of bone were few in number in comparison to DXA. The variability in the methods used to image bone, along with the anatomical sites assessed and range of outcomes reported at these sites, as well as the distinct heterogeneity in the accelerometer data collection and processing methods used meant it was not appropriate to conduct a meta-analysis. The vote-counting procedure that was conducted as a semi-quantitative alternative is, however, limited by the fact that it does not provide an estimate of the magnitude of an effect across the studies reviewed and is only able to identify whether or not there is evidence of an effect [83, 84]. The procedure also does not take into account study size, with larger studies that have greater statistical power being treated the same as smaller studies with less power, which may have introduced some bias to the results [83]. However, this approach provides an interpretable way of summarising study findings when a meta-analysis is not possible [83, 84]. The inclusion of studies that had conducted analyses between both MPA and/or MVPA and VPA may have also meant that any studies only assessing VPA in relation to bone outcomes will not have been included. However, inclusion of both intensities allows the potential benefit of one over the other to be observed.

Since short, dynamic bursts of activity are particularly important for bone health and are likely to have been misrepresented in studies assessing the associations between habitual PA and bone, there is a need for studies to be conducted that investigate the use of shorter 1-s epochs that are better able to identify more sporadically performed, high-intensity activity [85] or that use raw acceleration (where no epoch is applied) that has the resolution to capture impact peaks within the data [80] to ensure that this type of activity is captured more in its entirety. Improved bone-specific approaches to PA measurement will allow for a better understanding of important components (amount and intensity) of the dose–response relationship between PA and bone outcomes, which in turn will inform the design of PA interventions that aim to improve bone health in this population. Whilst stratifying for VPA in the present review allowed the independent contributions of this intensity to be explored, it is still a relatively broad category that includes both running and jumping activities, which actually differ in terms of impact magnitude. Running is classified as moderate-impact activity and jumping is a high-impact activity that has the potential to initiate a greater osteogenic response [27]. Whilst the magnitude of the accelerometry output is frequently classified in research according to the cardiovascular demands of activity, when osteogenic characteristics of PA are of interest, classifying accelerometer output in terms of loading (i.e. impact), which more closely reflects the physiological mechanisms underpinning bone adaptation [27], is likely to be more informative and would allow the osteogenic response of activities that differ in terms of impact magnitude to be more precisely examined. There is also a need to increase the comparability of findings between studies. Since methods for analysing raw acceleration data are still being developed, validated and optimised [82], averaging of raw data over shorter 1-s epochs using free to access open source software and investigating associations with a number of PA intensities (instead of MPA/MVPA and VPA which were designed to reflect steady-state aerobic intensities of activity) may present a more viable, readily accessible method for improving the monitoring and identification of bone-specific PA intensities and comparability of findings between studies.

In conclusion, whilst there is evidence to suggest a greater benefit of VPA over MPA/MVPA to bone outcomes in children and adolescents, at present, it is not possible to discern the precise amount of VPA required to be of benefit in this population. This is due to the considerable variation in the methods used to obtain accelerometer data, which greatly impact on the amount of VPA reported. Since there is currently no consensus for accelerometer methodology, it is unclear which methods most accurately reflect bone-specific activity habitually performed by children and adolescents. Future research needs to investigate whether the use of shorter epochs allows for more of the sporadic, high-impact activity performed by children and adolescents to be identified and whether more specific, bone-relevant intensities of activity that focus on impact and loading characteristics from raw accelerometry data should be explored and recommended over traditionally used classifications. A data-driven approach that identifies the intensities of free-living activity that are most strongly associated with bone health outcomes may be more informative than relying on and investigating the associations with pre-defined intensity classifications that have been calibrated against measures of energy expenditure and are more relevant to cardiovascular and metabolic health outcomes, as opposed to bone.

## Supplementary Information

Below is the link to the electronic supplementary material.

Supplementary file1 (DOCX 15 KB)

Supplementary file2 (DOCX 18 KB)

Supplementary file3 (DOCX 39 KB)

## Data Availability

The research data supporting this publication are provided within this paper.
