# Correction: A comparison of the associations between bone health and three different intensities of accelerometer-derived habitual physical activity in children and adolescents: a systematic review

**DOI:** 10.1007/s00198-022-06362-6

**Published:** 2022-03-29

**Authors:** Gemma Brailey, Brad Metcalf, Rebecca Lear, Lisa Price, Sean Cumming, Victoria Stiles

**Affiliations:** 1 Sport and Health Sciences, College of Life and Environmental Sciences, University of Exeter, Exeter, UK; 2 Department for Health, University of Bath, Bath, UK

**Osteoporosis International**

10.1007/s00198-021-06218-5

This article was originally published under a CC BY-NC 4.0 license, but has now been made available under a Creative Commons Attribution 4.0 International License (https://creativecommons.org/licenses/by/4.0/), which permits use, sharing, adaptation, distribution and reproduction in any medium or format, as long as you give appropriate credit to the original author(s) and the source, provide a link to the Creative Commons licence, and indicate if changes were made. license. The PDF and HTML versions of the paper have been modified accordingly.

