# Supplementary material for: A comparison of the associations between bone health and three different intensities of accelerometer-derived habitual physical activity in children and adolescents: a systematic review
Source: Osteoporos Int. 2022 Jan 28;33(6):1191–222. doi: 10.1007/s00198-021-06218-5 (PMC9106641; doi:10.1007/s00198-021-06218-5)
Supplement: Supplementary file 1 — Supplementary file1 (DOCX 15 KB) [file 198_2021_6218_MOESM1_ESM.docx]

**Title:** A comparison of the associations between bone health and three different intensities of accelerometer-derived habitual physical activity in children and adolescents: a systematic review

**Journal:** Osteoporosis International

**Author names:** Gemma Brailey, Dr Brad Metcalf, Rebecca Lear, Dr Lisa Price, Dr Sean Cumming, Dr Victoria Stiles

**Corresponding Author:** Gemma Brailey, Sport and Health Sciences, College of Life and Environmental Sciences, University of Exeter, Exeter, UK.

gb422@exeter.ac.uk

Example of a full search conducted in the Medline database:

**Ovid Search (Medline)**

| 1 | ((physical* or habitual*) adj2 activ*).ab,ti. |
| --- | --- |
| 2 | (sedentary adj2 (activ* or time or behaviour* or behavior*)).ab,ti. |
| 3 | ((moderate* or vigorous*) adj2 activ*).ab,ti. |
| 4 | MVPA.ab,ti. |
| 5 | (leisure adj time adj2 activ*).ab,ti. |
| 6 | (daily adj2 activ*).ab,ti. |
| 7 | (everyday adj2 activ*).ab,ti. |
| 8 | "acceleromet*".ab,ti. |
| 9 | "activity monitor*".ab,ti. |
| 10 | motion sensor?.ab,ti. |
| 11 | *Accelerometry/ |
| 12 | *Monitoring, Ambulatory/ |
| 13 | *Actigraphy/ |
| 14 | *Physical Exertion/ |
| 15 | *Motor Activity/ |
| 16 | 1 or 2 or 3 or 4 or 5 or 6 or 7 or 8 or 9 or 10 or 11 or 12 or 13 or 14 or 15 |
| 17 | (bone? adj2 health*).ab,ti. |
| 18 | (bone? adj2 densit*).ab,ti. |
| 19 | (bone? adj2 content*).ab,ti. |
| 20 | (bone? adj2 mass*).ab,ti. |
| 21 | (bone? adj2 area).ab,ti. |
| 22 | (bone? adj2 strength*).ab,ti. |
| 23 | (bone? adj2 geometr*).ab,ti. |
| 24 | (bone? adj2 (architecture or microarchitecture or micro-architecture)).ab,ti. |
| 25 | (bone? adj2 structur*).ab,ti. |
| 26 | (bone? adj2 microstructure).ab,ti. |
| 27 | *Bone Density/ |
| 28 | *Bone Development/ |
| 29 | *Osteogenesis/ |
| 30 | *"Bone and Bones"/ |
| 31 | 17 or 18 or 19 or 20 or 21 or 22 or 23 or 24 or 25 or 26 or 27 or 28 or 29 or 30 |
| 32 | (dual* adj3 absorptiometry).ab,ti. |
| 33 | (DXA or DEXA).ab,ti. |
| 34 | (single* adj2 absorptiometry).ab,ti. |
| 35 | "quantitative ultraso*".ab,ti. |
| 36 | (quantitative adj2 tomography).ab,ti. |
| 37 | (HRpQCT or HR pQCT or HR-pQCT).ab,ti. |
| 38 | (magnetic adj resonance adj imaging).ab,ti. |
| 39 | MRI.ab,ti. |
| 40 | (hip adj structural adj analys*).ab,ti. |
| 41 | (finite adj element adj analys*).ab,ti. |
| 42 | "densitometr*".ab,ti. |
| 43 | *Tomography, X-Ray Computed/ |
| 44 | *Absorptiometry, Photon/ |
| 45 | *Magnetic Resonance Imaging/ |
| 46 | 32 or 33 or 34 or 35 or 36 or 37 or 38 or 39 or 40 or 41 or 42 or 43 or 44 or 45 |
| 47 | "child*".ab,ti. |
| 48 | "adolescen*".ab,ti. |
| 49 | (schoolchild* or school-child*).ab,ti. |
| 50 | (school adj child*).ab,ti. |
| 51 | (paediatric* or pediatric*).ab,ti. |
| 52 | (young or youth?).ab,ti. |
| 53 | "teen*".ab,ti. |
| 54 | (girl? or boy?).ab,ti. |
| 55 | (pubert* or peripubert* or pre-pubert* or post-pubert*).ab,ti. |
| 56 | (pubescent or pre-pubescent or post-pubescent).ab,ti. |
| 57 | exp Puberty/ |
| 58 | exp Child/ |
| 59 | exp Adolescent/ |
| 60 | 47 or 48 or 49 or 50 or 51 or 52 or 53 or 54 or 55 or 56 or 57 or 58 or 59 |
| 61 | 16 and (31 or 46) and 60 |
