# Supplementary material for: A comparison of the associations between bone health and three different intensities of accelerometer-derived habitual physical activity in children and adolescents: a systematic review
Source: Osteoporos Int. 2022 Jan 28;33(6):1191–222. doi: 10.1007/s00198-021-06218-5 (PMC9106641; doi:10.1007/s00198-021-06218-5)
Supplement: Supplementary file 2 — Supplementary file2 (DOCX 18 KB) [file 198_2021_6218_MOESM2_ESM.docx]

**Title:** A comparison of the associations between bone health and three different intensities of accelerometer-derived habitual physical activity in children and adolescents: a systematic review

**Journal:** Osteoporosis International

**Author names:** Gemma Brailey, Dr Brad Metcalf, Rebecca Lear, Dr Lisa Price, Dr Sean Cumming, Dr Victoria Stiles

**Corresponding Author:** Gemma Brailey, Sport and Health Sciences, College of Life and Environmental Sciences, University of Exeter, Exeter, UK.

gb422@exeter.ac.uk

Study quality assessment using the National Institute for Health Quality Assessment Tool for Observational Cohort and Cross-Sectional Studies

| First Author (ref) | Q1 | Q2 | Q3 | Q4 | Q5 | Q6 | Q7 | Q8 | Q9 | Q10 | Q11 | Q12 | Q13 | Q14 | Rating |
| --- | --- | --- | --- | --- | --- | --- | --- | --- | --- | --- | --- | --- | --- | --- | --- |
| Bielemann 2019 (40) | Y | Y | Y | Y | N | N | N | Y | N | N | Y | NR | NA | Y | Fair |
| Cardadeiro 2010 (43) | Y | Y | CD | Y | N | N | N | Y | N | N | N | NR | NA | N | Poor |
| Cardadeiro 2012 (42) | Y | Y | CD | Y | N | N | N | Y | N | N | Y | NR | NA | Y | Fair |
| Cardadeiro 2014a (41) | Y | Y | CD | CD | N | N | N | Y | Y | N | Y | NR | NA | Y | Fair |
| Cardadeiro 2014b (64) | Y | Y | CD | Y | N | N | Y | Y | Y | Y | Y | NR | CD | Y | Good |
| De Smet 2015 (44) | Y | Y | CD | Y | N | N | N | Y | N | N | Y | NR | NA | N | Fair |
| Deere 2012a (45) | Y | Y | N | Y | N | N | N | Y | N | N | Y | NR | NA | Y | Fair |
| Deere 2012b (46) | Y | Y | N | Y | N | N | N | Y | N | N | Y | NR | NA | Y | Fair |
| Gracia-Marco 2011 (47) | Y | Y | CD | Y | N | N | N | Y | N | N | Y | NR | NA | Y | Fair |
| Hasselstrom 2007 (48) | Y | Y | Y | Y | N | N | N | Y | N | N | Y | NR | NA | N | Fair |
| Herrmann 2015 (49) | Y | Y | N | Y | N | N | N | Y | N | N | Y | NR | NA | Y | Fair |
| Ivuškāns 2015 (67) | Y | Y | CD | Y | N | N | Y | Y | N | Y | Y | NR | Y | Y | Good |
| Janz 2004 (50) | Y | Y | CD | Y | N | N | N | Y | N | N | Y | NR | NA | Y | Fair |
| Janz 2014 (65) | Y | Y | CD | Y | N | N | Y | Y | N | Y | Y | NR | Y | Y | Good |
| Kehrig 2018 (51) | Y | Y | N | Y | N | N | N | Y | N | N | Y | NR | NA | Y | Fair |
| Marin-Puyalto 2019 (52) | Y | Y | Y | Y | N | N | N | Y | N | N | Y | NR | NA | Y | Fair |
| McCormack 2016 (53) | Y | Y | CD | Y | N | N | N | Y | Y | N | Y | NR | NA | N | Fair |
| Meiring 2014 (39) | Y | Y | CD | Y | Y | N | N | Y | Y | N | Y | NR | NA | Y | Fair |
| Muñoz-Hernandez 2018 (54) | Y | Y | Y | N | N | N | N | Y | Y | N | Y | NR | NA | Y | Fair |
| Sardinha 2008 (55) | Y | Y | Y | Y | N | N | N | Y | N | N | Y | NR | NA | Y | Fair |
| Sayers 2011 (24) | Y | Y | N | Y | N | N | N | Y | N | N | Y | NR | NA | Y | Fair |
| Sioen 2015 (56) | Y | Y | N | Y | N | N | N | Y | N | N | Y | NR | NA | Y | Fair |
| Specker 2001 (57) | Y | Y | CD | Y | N | N | N | Y | N | N | Y | NR | NA | N | Poor |
| Szmodis 2019 (58) | Y | Y | N | Y | N | N | N | Y | Y | N | Y | NR | NA | N | Poor |
| Tamme 2019 (66) | Y | Y | N | CD | N | Y | Y | Y | N | Y | Y | NR | Y | Y | Good |
| Tan 2018 (59) | Y | Y | N | Y | N | N | N | Y | N | N | Y | NR | NA | Y | Fair |
| Tobias 2007 (60) | Y | Y | Y | Y | N | N | N | Y | N | N | Y | NR | NA | Y | Fair |
| Torres-Costoso 2015 (61) | Y | Y | CD | Y | N | N | N | Y | Y | N | Y | NR | NA | N | Poor |
| Yamakita 2019 (62) | Y | Y | N | Y | N | N | N | Y | Y | N | Y | NR | NA | N | Poor |
| Yao 2011 (63) | Y | Y | CD | Y | N | N | N | Y | Y | N | Y | NR | NA | N | Poor |

Y= yes; N= no; CD= cannot determine; NR= not reported; NA= not applicable; **Q1:** Was the research question or objective in this paper clearly stated? **Q2:** Was the study population clearly defined? **Q3:** Was the participation rate of eligible persons at least 50%? **Q4:** Were all the subjects selected or recruited from the same or similar populations (including the same time period)? Were inclusion and exclusion criteria for being in the study prespecified and applied uniformly to all participants? **Q5:** Was a sample size justification, power description, or variance and effect estimates provided? **Q6:** For the analyses in this paper, were the exposure(s) of interest measured prior to the outcomes(s) being measured? **Q7:** Was the timeframe sufficient so that one could reasonably expect to see an association between exposure and outcome if it existed? **Q8:** For exposures that can vary in amount or level, did the study examine different levels of the exposure as related to the outcome (e.g., categories of exposure, or exposure measured as continuous variable)? **Q9:** Were the exposure measures (independent variables) clearly defined, valid, reliable, and implemented consistently across all study participants? NB: This item was adapted so that only studies with ≥4 days with ≥10 hours of accelerometer data for inclusion were able to obtain a ‘yes’ response, as this is deemed representative of habitual PA in children. **Q10:** Was the exposure(s) assessed more than once over time? **Q11:** Were the outcome measures (dependent variables) clearly defined, valid, reliable, and implemented consistently across all study participants? **Q12:** Were the outcome assessors blinded to the exposure status of participants? **Q13:** Was loss to follow-up after baseline 20% or less? **Q14:** Were key potential confounding variables measured and adjusted statistically for their impact on the relationship between exposure(s) and outcome(s)? NB: Q14 was based on the analyses that reported/compared all activity intensities of interest (MPA and/or MVPA **and** VPA).
