# Supplementary material for: A comparison of the associations between bone health and three different intensities of accelerometer-derived habitual physical activity in children and adolescents: a systematic review
Source: Osteoporos Int. 2022 Jan 28;33(6):1191–222. doi: 10.1007/s00198-021-06218-5 (PMC9106641; doi:10.1007/s00198-021-06218-5)
Supplement: Supplementary file 3 — Supplementary file3 (DOCX 39 KB) [file 198_2021_6218_MOESM3_ESM.docx]

**Title:** A comparison of the associations between bone health and three different intensities of accelerometer-derived habitual physical activity in children and adolescents: a systematic review

**Journal:** Osteoporosis International

**Author names:** Gemma Brailey, Dr Brad Metcalf, Rebecca Lear, Dr Lisa Price, Dr Sean Cumming, Dr Victoria Stiles

**Corresponding Author:** Gemma Brailey, Sport and Health Sciences, College of Life and Environmental Sciences, University of Exeter, Exeter, UK.

gb422@exeter.ac.uk

Vote count tables for each anatomical site and each outcome assessed for DXA, pQCT and QUS.

Results from the vote count for each anatomical site and each outcome reported at that site for studies using DXA to measure bone outcomes. In stage 1, votes were counted based on whether each intensity was statistically significant (p<0.05; 1= yes). In stage 2, out of the significant intensities only the intensity with the largest effect size (association) received a vote (only 1 count available out of the 2/3 intensities). Votes were counted for all analyses for each outcome included in a study (e.g for whole sample, boys and girls). When the value of association was the same for two intensities, votes were counted if significant in stage 1, but a stage 2 vote was not cast. When negative associations were observed, their significance was noted but again, no stage 2 vote was cast. Results are presented as the proportion of significant/most strongly associated counts out of the total number of counts available for each intensity (total counts are regardless of statistical significance), followed by the number of significant/most strongly associated counts and the number of total counts available for each intensity (% (n/N))

|  | **Stage 1:** PA associations that were statistically significant (p<0.05) | | | **Stage 2:** PA associations that were the strongest within a study | | |
| --- | --- | --- | --- | --- | --- | --- |
|  | MPA sig.  % (n/N) | MVPA sig.  % (n/N) | VPA sig.  % (n/N) | MPA  strongest assoc.  % (n/N) | MVPA strongest assoc.  % (n/N) | VPA  strongest assoc.  % (n/N) |
| **Whole Body** |  |  |  |  |  |  |
| BMC (g) | 27% (3/11) | 18% (2/11) | 43% (6/14) | 18% (2/11) | 0% (0/11) | 21% (3/14) |
| BMD (g/cm²) | 29% (2/7) | 29% (2/7) | 25% (2/8) | 14% (1/7) | 0% (0/7) | 0% (0/8) |
| BMAD (g/cm³) | 0% (0/1) | 0% (0/1) | 0% (0/1) | 0% (0/1) | 0% (0/1) | 0% (0/1) |
| BA (cm²) | 50% (1/2) | 33% (1/3) | 33% (1/3) | 50% (1/2) | 0% (0/3) | 0% (0/3) |
| aBMC (g) | 100% (1/1) | 100% (1/1) | 0% (0/1) | 100% (1/1) | 0% (0/1) | 0% (0/1) |
| BMC/height | 0% (0/1) | 0% (0/1) | 0% (0/1) | 0% (0/1) | 0% (0/1) | 0% (0/1) |
| **Total** | **30% (7/23)** | **25% (6/24)** | **32% (9/28)** | **22% (5/23)** | **0% (0/24)** | **11% (3/28)** |
| **Lumbar Spine** |  |  |  |  |  |  |
| BMC (g) | 0% (0/6) | 17% (1/6) | 33% (3/9) | 0% (0/6) | 0% (0/6) | 33% (3/9) |
| BMD (g/cm²) | 20% (1/5) | 0% (0/3) | 17% (1/6) | 20% (1/5) | 0% (0/3) | 17% (1/6) |
| BA (cm²) | 0% (0/1) | 0% (0/1) | 0% (0/1) | 0% (0/1) | 0% (0/1) | 0% (0/1) |
| BMAD (g/cm³) | 0% (0/1) | 0% (0/1) | 0% (0/1) | 0% (0/1) | 0% (0/1) | 0% (0/1) |
| **Total** | **8% (1/13)** | **9% (1/11)** | **24% (4/17)** | **8% (1/13)** | **0% (0/11)** | **24% (4/17)** |
| **Hip** |  |  |  |  |  |  |
| BMC (g) | 0% (0/1) | 50% (2/4) | 100% (4/4) | 0% (0/1) | 0% (0/4) | 100% (4/4) |
| BMD (g/cm²) | - | 0% (0/2) | 100% (2/2) | - | 0% (0/2) | 100% (2/2) |
| CSA (cm²) | 67% (4/6) | - | 100% (6/6) | 0% (0/6) | - | 100% (6/6) |
| Z (cm³) | 50% (3/6) | - | 100% (6/6) | 0% (0/6) | - | 83% (5/6) |
| **Total** | **54% (7/13)** | **33% (2/6)** | **100% (18/18)** | **0% (0/13)** | **0% (0/6)** | **94% (17/18)** |
| **Femoral Neck^a^** |  |  |  |  |  |  |
| BMC (g) | 33% (2/6) | 59% (2/4) | 86% (6/7) | 17% (1/6) | 0% (0/4) | 86% (6/7) |
| BMD (g/cm²) | 18% (3/17) | 29% (4/14) | 44% (8/18) | 6% (1/17) | 20% (2/14) | 44% (8/18) |
| BA (cm²) | 0% (0/1) | 0% (0/1) | 0% (0/1) | 0% (0/1) | 0% (0/1) | 0% (0/1) |
| Strength^b^ (g/kg) | 50% (3/6) | - | 100% (6/6) | 17% (1/6) | - | 83% (5/6) |
| Width (mm) | 0% (0/1) | - | 100% (1/1) | 0% (0/1) | - | 100% (1/1) |
| Cortical thickness (mm) | 0% (0/1) | - | 0% (0/1) | 0% (0/1) | - | 0% (0/1) |
| CSMI (cm⁴) | 0% (0/1) | - | 100% (1/1) | 0% (0/1) | - | 100% (1/1) |
| **Total** | **24% (8/33)** | **32% (6/19)** | **63% (22/35)** | **9% (3/33)** | **11% (2/19)** | **60% (21/35)** |
| **Trochanter** |  |  |  |  |  |  |
| BMC (g) | - | 0% (0/1) | 0% (0/1) | - | 0% (0/1) | 0% (0/1) |
| BMD (g/cm²) | 14% (1/7) | 13% (1/8) | 50% (4/8) | 14% (1/7) | 13% (1/8) | 50% (4/8) |
| **Total** | **14% (1/7)** | **11% (1/9)** | **44% (4/9)** | **14% (1/7)** | **11% (1/9)** | **44% (4/9)** |
| **Intertrochanter** |  |  |  |  |  |  |
| BMC (g) | - | 0% (0/1) | 0% (0/1) | - | 0% (0/1) | 0% (0/1) |
| BMD (g/cm²) | 20% (1/5) | 17% (1/6) | 67% (4/6) | 20% (1/5) | 17% (1/6) | 67% (4/6) |
| **Total** | **20% (1/5)** | **14% (1/7)** | **57% (4/7)** | **20% (1/5)** | **14% (1/7)** | **57% (4/7)** |
| **Ward’s Area** |  |  |  |  |  |  |
| **Proximal shape variation** | **50% (1/2)** | **50% (1/2)** | **0% (0/2)** | **50% (1/2)** | **0% (0/2)** | **0% (0/2)** |
| **Upper limbs** |  |  |  |  |  |  |
| BMC (g) | 33% (1/3) | 67% (2/3) | 33% (1/3) | 0% (0/3) | 0% (0/3) | 33% (1/3) |
| BMD (g/cm²) | 33% (1/3) | 67% (2/3) | 33% (1/3) | 0% (0/3) | 0% (0/3) | 33% (1/3) |
| BA (cm²) | 0% (0/1) | 100% (1/1) | 100% (1/1) | 0% (0/1) | 0% (0/1) | 100% (1/1) |
| aBMC (g) | 0% (0/1) | 100% (1/1) | 0% (0/1) | 0% (0/1) | 100% (1/1) | 0% (0/1) |
| **Total** | **25% (2/8)** | **75% (6/8)** | **38% (3/8)** | **0% (0/8)** | **13% (1/8)** | **38% (3/8)** |
| **Lower limbs** |  |  |  |  |  |  |
| BMC (g) | 67% (2/3) | 67% (2/3) | 33% (1/3) | 33% (1/3) | 0% (0/3) | 0% (0/3) |
| BMD (g/cm²) | 67% (2/3) | 67% (2/3) | 33% (1/3) | 33% (1/3) | 0% (0/3) | 33% (1/3) |
| BA (cm²) | 100% (1/1) | 100% (1/1) | 0% (0/1) | 100% (1/1) | 0% (0/1) | 0% (0/1) |
| aBMC (g) | 100% (1/1) | 100% (1/1) | 0% (0/1) | 100% (1/1) | 0% (0/1) | 0% (0/1) |
| **Total** | **75% (6/8)** | **75% (6/8)** | **25% (2/8)** | **50% (4/8)** | **0% (0/8)** | **13% (1/8)** |
| **Calcaneus** |  |  |  |  |  |  |
| **BMD (g/cm²)** | **-** | **100% (1/1)** | **100% (1/1)** | **-** | **0% (0/1)** | **100% (1/1)** |
| **Distal forearm** |  |  |  |  |  |  |
| BMD (g/cm²) | - | 100% (1/1) | 100% (1/1) | - | 0% (0/1) | 100% (1/1) |
| BMC (g) | 0% (0/2) | 0% (0/2) | 0% (0/2) | 0% (0/2) | 0% (0/2) | 0% (0/2) |
| **Total** | **0% (0/2)** | **33% (1/3)** | **33% (1/3)** | **0% (0/2)** | **0% (0/3)** | **33% (1/3)** |
|  |  |  |  |  |  |  |
| **DXA all sites and outcomes:** | **30% (34/114)** | **33% (32/98)** | **50% (68/136)** | **14% (16/114)** | **5% (5/98)** | **43% (59/136)** |
| **p-value (vs VPA)** | **p=0.002** | **p=0.016** | **-** | **p<0.001** | **p<0.001** | **-** |

DXA= dual-energy x-ray absorptiometry; BMC= bone mineral content; BMD= bone mineral density; BMAD= bone mineral apparent density; BA= bone area; aBMC= area-adjusted BMC; CSMI= cross-sectional moment of inertia; CSA= cross-sectional area; Z= section modulus ^a^ Femoral neck consists of counts for the whole femoral neck, as well as the femoral neck subregions (superlateral FN and inferomedial FN); ^b^ Strength refers to measures of compressive, bending and impact strength.

p-value (vs VPA) = these are the Bonferroni adjusted p-values from the 2x2 Chi-square tests for ‘MPA vs VPA’ and ‘MVPA vs VPA’ when the omnibus 3x2 Chi-square test indicates that there is a significant difference between at least two of the three intensities. The p-values in bold font indicate significance at the 5% level.

Results from the vote count for each anatomical site and each outcome reported at that site for studies using pQCT to measure bone outcomes. In stage 1, votes were counted based on whether each intensity was statistically significant (p<0.05; 1= yes). In stage 2, out of the significant intensities only the intensity with the largest effect size (association) received a vote (only 1 count available out of the 2/3 intensities). Votes were counted for all analyses for each outcome included in a study (e.g for whole sample, boys and girls). When the value of association was the same for two intensities, votes were counted if significant in stage 1, but a stage 2 vote was not cast. When negative associations were observed, their significance was noted but again, no stage 2 vote was cast. Results are presented as the proportion of significant/most strongly associated counts out of the total number of counts available for each intensity (total counts are regardless of statistical significance), followed by the number of significant/most strongly associated counts and the number of total counts available for each intensity (% (n/N))

|  | **Stage 1:** PA associations that were statistically significant (p<0.05) | | | **Stage 2:** PA associations that were the strongest within a study | | | |
| --- | --- | --- | --- | --- | --- | --- | --- |
|  | MPA sig.  % (n/N) | MVPA sig.  % (n/N) | VPA sig.  % (n/N) | MPA  strongest assoc.  % (n/N) | MVPA  strongest assoc.  % (n/N) | | VPA  strongest assoc.  % (n/N) |
| **66% tibia** |  |  |  |  |  |  | |
| **Polar SSI (mm³)** | **-** | **100% (1/1)** | **100% (1/1)** | **-** | **0% (0/1)** | **100% (1/1)** | |
| **65% tibia** |  |  |  |  |  |  | |
| Total area (mm^2^) | 0% (0/1) | 0% (0/1) | 0% (0/1) | 0% (0/1) | 0% (0/1) | 0% (0/1) | |
| Cortical BA (mm^2^) | 0% (0/1) | 0% (0/1) | 0% (0/1) | 0% (0/1) | 0% (0/1) | 0% (0/1) | |
| Cortical BMD (mg/cm³) | 0% (0/1) | 0% (0/1) | 0% (0/1) | 0% (0/1) | 0% (0/1) | 0% (0/1) | |
| SSI (mm^3^) | 0% (0/1) | 0% (0/1) | 0% (0/1) | 0% (0/1) | 0% (0/1) | 0% (0/1) | |
| Periosteal circumference (mm) | 0% (0/1) | 0% (0/1) | 0% (0/1) | 0% (0/1) | 0% (0/1) | 0% (0/1) | |
| Endosteal circumference | 0% (0/1) | 0% (0/1) | 0% (0/1) | 0% (0/1) | 0% (0/1) | 0% (0/1) | |
| Cortical thickness (mm) | 0% (0/1) | 0% (0/1) | 0% (0/1) | 0% (0/1) | 0% (0/1) | 0% (0/1) | |
| **Total** | **0% (0/7)** | **0% (0/7)** | **0% (0/7)** | **0% (0/7)** | **0% (0/7)** | **0% (0/7)** | |
| **50% (midshaft) tibia** |  |  |  |  |  |  | |
| Cortical BMC (mg) | 0% (0/6) | - | 50% (3/6) | 0% (0/6) | - | 50% (3/6) | |
| Cortical BA (mm²) | 17% (1/6) | 0% (0/1) | 43% (3/7) | 0% (0/6) | 0% (0/1) | 43% (3/7) | |
| Cortical BMD (mg/cm³) | 17% (1/6) | 0% (0/1) | 29% (2/7) | 0% (0/6) | 0% (0/1) | 29% (2/7) | |
| Periosteal circumference (mm) | 0% (0/6) | - | 50% (3/6) | 0% (0/6) | - | 50% (3/6) | |
| Endosteal circumference (mm) | 0% (0/3) | - | 100% (3/3) | 0% (0/3) | - | 100% (3/3) | |
| Total bone CSA (mm²) | - | 0% (0/1) | 0% (0/1) | - | 0% (0/1) | 0% (0/1) | |
| Medullary area (mm²) | - | 0% (0/1) | 0% (0/1) | - | 0% (0/1) | 0% (0/1) | |
| Polar SSI (mm³) | - | 100% (1/1) | 0% (0/1) | - | 100% (1/1) | 0% (0/1) | |
| SSI | 0% (0/3) | - | 33% (1/3) | 0% (0/3) | - | 33% (1/3) | |
| CSMI (cm⁴) | 0% (0/3) | - | 33% (1/3) | 0% (0/3) | - | 33% (1/3) | |
| Buckling Ratio | 0% (0/3) | - | 0% (0/3) | 0% (0/3) | - | 0% (0/3) | |
| Cortical thickness (mm) | 0% (0/3) | - | 0% (0/3) | 0% (0/3) | - | 0% (0/3) | |
| **Total** | **5% (2/39)** | **20% (1/5)** | **36% (16/44)** | **0% (0/39)** | **20% (1/5)** | **36% (16/44)** | |
| **20% tibia** |  |  |  |  |  |  | |
| Cortical BA (mm²) | - | 0% (0/1) | 0% (0/1) | - | 0% (0/1) | 0% (0/1) | |
| Periosteal circumference (mm) | - | 0% (0/1) | 0% (0/1) | - | 0% (0/1) | 0% (0/1) | |
| Endosteal circumference (mm) | - | 0% (0/1) | 0% (0/1) | - | 0% (0/1) | 0% (0/1) | |
| **Total** | **-** | **0% (0/3)** | **0% (0/3)** | **-** | **0% (0/3)** | **0% (0/3)** | |
|  |  |  |  |  |  |  | |
| **8% tibia** |  |  |  |  |  |  | |
| Total bone CSA (mm²) | - | 0% (0/1) | 0% (0/1) | - | 0% (0/1) | 0% (0/1) | |
| BSI (mg²/mm⁴) | - | 0% (0/1) | 100% (1/1) | - | 0% (0/1) | 100% (1/1) | |
| Total bone density (mg/cm³) | - | 0% (0/1) | 0% (0/1) | - | 0% (0/1) | 0% (0/1) | |
| **Total** | **-** | **0% (0/3)** | **33% (1/3)** | **-** | **0% (0/3)** | **33% (1/3)** | |
|  |  |  |  |  |  |  | |
| **4% tibia** |  |  |  |  |  |  | |
| **BSI (mg²/mm⁴)** | **-** | **0% (0/1)** | **100% (1/1)** | **-** | **0% (0/1)** | **100% (1/1)** | |
|  |  |  |  |  |  |  | |
| **Radius shaft (65%)** |  |  |  |  |  |  | |
| Total area (mm^2^) | 0% (0/1) | 0% (0/1) | 0% (0/1) | 0% (0/1) | 0% (0/1) | 0% (0/1) | |
| Cortical BA (mm^2^) | 0% (0/1) | 0% (0/1) | 100% (1/1) | 0% (0/1) | 0% (0/1) | 100% (1/1) | |
| Cortical BMD (mg/cm³) | 0% (0/1) | 0% (0/1) | 0% (0/1) | 0% (0/1) | 0% (0/1) | 0% (0/1) | |
| SSI (mm^3^) | 0% (0/1) | 0% (0/1) | 0% (0/1) | 0% (0/1) | 0% (0/1) | 0% (0/1) | |
| Periosteal circumference (mm) | 0% (0/1) | 0% (0/1) | 0% (0/1) | 0% (0/1) | 0% (0/1) | 0% (0/1) | |
| Endosteal circumference | 0% (0/1) | 0% (0/1) | 0% (0/1) | 0% (0/1) | 0% (0/1) | 0% (0/1) | |
| Cortical thickness (mm) | 0% (0/1) | 0% (0/1) | 0% (0/1) | 0% (0/1) | 0% (0/1) | 0% (0/1) | |
| Polar SSI (mm³) | - | 0% (0/1) | 0% (0/1) | - | 0% (0/1) | 0% (0/1) | |
| **Total** | **0% (0/7)** | **0% (0/8)** | **13% (1/8)** | **0% (0/7)** | **0% (0/8)** | **13% (1/8)** | |
| **Distal Radius (4%)** |  |  |  |  |  |  | |
| Total area (mm^2^) | 0% (0/1) | 0% (0/1) | 0% (0/1) | 0% (0/1) | 0% (0/1) | 0% (0/1) | |
| Total density (mg/cm^3^) | 100% (1/1) | 100% (1/1) | 0% (0/1) | 100% (1/1) | 0% (0/1) | 0% (0/1) | |
| Trabecular density (mg/cm^3^) | 0% (0/1) | 0% (0/1) | 0% (0/1) | 0% (0/1) | 0% (0/1) | 0% (0/1) | |
| BSI (mg²/mm⁴) | 100% (1/1) | 0% (0/2) | 0% (0/2) | 100% (1/1) | 0% (0/2) | 0% (0/2) | |
| **Total** | **50% (2/4)** | **20% (1/5)** | **0% (0/5)** | **50% (2/4)** | **0% (0/5)** | **0% (0/5)** | |
|  |  |  |  |  |  |  | |
| **pQCT all sites & outcomes** | **7% (4/57)** | **9% (3/33)** | **28% (20/72)** | **4% (2/57)** | **3% (1/33)** | **28% (20/72)** | |
| **p-value (vs VPA)** | **p=0.005** | p=0.063 | **-** | **p<0.001** | **p=0.007** | **-** | |

pQCT= peripheral quantitative computed tomography; BMC= bone mineral content; BA= bone area; BMD= bone mineral density; CSA= cross-sectional area; SSI= strength-strain index; CSMI= cross-sectional moment of inertia; BSI= bone strength index

p-value (vs VPA) = these are the Bonferroni adjusted p-values from the 2x2 Chi-square tests for ‘MPA vs VPA’ and ‘MVPA vs VPA’ when the omnibus 3x2 Chi-square test indicates that there is a significant difference between at least two of the three intensities. The p-values in bold font indicate significance at the 5% level.

Results from the vote count for each anatomical site and each outcome reported at that site for studies using QUS to measure bone outcomes. In stage 1, votes were counted based on whether each intensity was statistically significant (p<0.05; 1= yes). In stage 2, out of the significant intensities only the intensity with the largest effect size (association) received a vote (only 1 count available out of the 2/3 intensities). Votes were counted for all analyses for each outcome included in a study (e.g for whole sample, boys and girls). When the value of association was the same for two intensities, votes were counted if significant in stage 1, but a stage 2 vote was not cast. When negative associations were observed, their significance was noted but again, no stage 2 vote was cast. Results are presented as the proportion of significant/most strongly associated counts out of the total number of counts available for each intensity (total counts are regardless of statistical significance), followed by the number of significant/most strongly associated counts and the number of total counts available for each intensity (% (n/N))

|  | **Stage 1:** PA associations that were statistically significant (p<0.05) | | | **Stage 2:** PA associations that were the strongest within a study | | |
| --- | --- | --- | --- | --- | --- | --- |
|  | MPA sig.  % (n/N) | MVPA sig.  % (n/N) | VPA sig.  % (n/N) | MPA  strongest assoc.  % (n/N) | MVPA strongest assoc.  % (n/N) | VPA  strongest assoc.  % (n/N) |
| **Calcaneus** |  |  |  |  |  |  |
| SI | 60% (3/5) | 80% (4/5) | 80% (4/5) | 0% (0/5) | 20% (1/5) | 60% (3/5) |
| BUA (dB/MHz) | 0% (0/4) | 25% (1/4) | 75% (3/4) | 0% (0/4) | 0% (0/4) | 75% (3/4) |
| SOS (m/s) | 0% (0/4) | 0% (0/4) | 50% (2/4) | 0% (0/4) | 0% (0/4) | 50% (2/4) |
| BQI | 0% (0/3) | 0% (0/3) | 67% (2/3) | 0% (0/3) | 0% (0/3) | 67% (2/3) |
| **Total** | **19% (3/16)** | **31% (5/16)** | **69% (11/16)** | **0% (0/16)** | **6% (1/16)** | **63% (10/16)** |
|  |  |  |  |  |  |  |
| **Midshaft tibia** |  |  |  |  |  |  |
| **SOS (m/s)** | **100% (2/2)** | **100% (2/2)** | **100% (2/2)** | **0% (0/2)** | **50% (1/2)** | **50% (1/2)** |
|  |  |  |  |  |  |  |
| **Distal radius** |  |  |  |  |  |  |
| **SOS (m/s)** | **0% (0/2)** | **0% (0/2)** | **0% (0/2)** | **0% (0/2)** | **0% (0/2)** | **0% (0/2)** |
|  |  |  |  |  |  |  |
| **QUS all sites & outcomes** | **25% (5/20)** | **35% (7/20)** | **65% (13/20)** | **0% (0/20)** | **10% (2/20)** | **55% (11/20)** |
| **p-value (vs VPA)** | **p=0.022** | p=0.116 | **-** | ***p<0.001** | ***p=0.011** | **-** |

QUS= quantitative ultrasound; SI= stiffness index; BUA= broadband ultrasound attenuation; SOS= speed of sound; BQI= bone quality index.

p-value (vs VPA) = these are the Bonferroni adjusted p-values from the 2x2 Chi-square tests (* = Fishers exact test) for ‘MPA vs VPA’ and ‘MVPA vs VPA’ when the omnibus 3x2 Chi-square test (* = Fisher’s exact test) indicates that there is a significant difference between at least two of the three intensities. The p-values in bold font indicate significance at the 5% level.

Results from the vote count by epoch length (≥60 s or ≤ 15 s) for all included studies. In stage 1, votes were counted based on whether each intensity was statistically significant (p<0.05; 1= yes). In stage 2, out of the significant intensities only the intensity with the largest effect size (association) received a vote (only 1 count available out of the 2/3 intensities). Votes were counted for all analyses for each outcome included in a study (e.g for whole sample, boys and girls). When the value of association was the same for two intensities, votes were counted if significant in stage 1, but a stage 2 vote was not cast. When negative associations were observed, their significance was noted but again, no stage 2 vote was counted. Results are presented as the proportion of significant/most strongly associated counts out of the total number of counts (total counts are regardless of statistical significance) available for each intensity, followed by the number of significant/most strongly associated counts and the number of total counts available for each intensity (% (n/N)).

|  | **Stage 1:** PA associations that were statistically significant (p<0.05) | | | **Stage 2:** PA associations that were the strongest within a study | | |
| --- | --- | --- | --- | --- | --- | --- |
| Epoch length | MPA sig.  % (n/N) | MVPA sig.  % (n/N) | VPA sig.  % (n/N) | MPA  strongest assoc.  % (n/N) | MVPA strongest assoc.  % (n/N) | VPA  strongest assoc.  % (n/N) |
| ≥60 s | 32% (24/75) | 48% (23/48) | 72% (63/87) | 13% (10/75) | 4% (2/48) | 63% (55/87) |
| ≤ 15 s | 14% (13/95) | 16% (13/82) | 27% (32/120) | 8% (8/95) | 7% (6/82) | 27% (32/120) |

NB: total N is less as two studies did not report the length of epoch used and were therefore not included in the analysis
